# *N*-Benzylethanolammonium Ionic Liquids and Molten Salts in the Synthesis of ^68^Ga- and Al^18^F-Labeled Radiopharmaceuticals

**DOI:** 10.3390/pharmaceutics15020694

**Published:** 2023-02-18

**Authors:** Yulia A. Kondratenko, Julia S. Shilova, Vladislav A. Gavrilov, Andrey A. Zolotarev, Michail A. Nadporojskii, Tatyana A. Kochina, Dmitrii O. Antuganov

**Affiliations:** 1 Grebenshchikov Institute of Silicate Chemistry RAS, Nab. Makarova, 2, 199034 Saint-Petersburg, Russia; 2 St. Petersburg State Technological Institute, Technical University, 26 Moskovsky Pr., 190013 Saint-Petersburg, Russia; 3 Granov Russian Research Center of Radiology & Surgical Technologies, Leningradskaya Str. 70, Pesochny, 197758 Saint-Petersburg, Russia; 4 Institute of Earth Sciences, St. Petersburg State University, University Emb. 7/9, 199034 Saint-Petersburg, Russia

**Keywords:** Good’s buffer ionic liquids, *N*-benzylethanolamine, alkanolammonium salts, radiopharmaceuticals, nuclear medicine, PET

## Abstract

Ionic liquids (ILs), due to their structural features, have unique physical and chemical properties and are environmentally friendly. Every year, the number of studies devoted to the use of ILs in medicine and pharmaceutics is growing. In nuclear medicine, the use of ILs with self-buffering capacity in the synthesis of radiopharmaceuticals is extremely important. This research is devoted to obtaining new ionic buffer agents containing *N*-benzylethanolammonium (BEA) cations and anions of carboxylic acids. A series of new BEA salts was synthesized and identified by NMR (^1^H, ^13^C), IR spectroscopy and elemental and thermal analysis. The crystal structures of BEA hydrogen succinate, hydrogen oxalate and oxalate were determined by x-ray diffraction. Newly synthesized compounds were tested as buffer solutions in ^68^Ga- and Al^18^F-radiolabeling reactions with a series of bifunctional chelating agents and clinically relevant peptides used for visualization of malignancies by positron emission tomography. The results obtained confirm the promise of using new buffers in the synthesis of ^68^Ga- and Al^18^F-labeled radiopharmaceuticals.

## 1. Introduction

Ionic liquids (ILs) are commonly defined as a broad class of ionic compounds containing unsymmetrical organic cations and organic/inorganic anions with melting points below 100 °C [1,2]. ILs have been a topic of interest in many scientific fields since the mid-1990s [3]. In recent years, the use of “green” ILs in pharmaceutics and medicine has increased dramatically. The green solvent and designer properties of ILs make them highly valuable for drug delivery systems [3]. Currently, the field of application of biocompatible ILs has expanded significantly and includes protein stabilization, the development of active pharmaceutical ingredients, the delivery of macromolecules, antimicrobial agents, the modification of nanocarriers and biosensing [4,5]. ILs readily form intermolecular contacts with a range of biomolecules such as proteins and DNA. Their ability to stabilize or destabilize the three-dimensional structure of a protein or the double helix structure of DNA is superior to water and volatile organic solvents [6]. ILs have been studied as solvents, reagents, or catalysts in the synthesis of active pharmaceutical ingredients and have been used in drug crystallization [7].

ILs containing choline cations are widely used in biomedicine. The toxicity of choline-based ILs is strongly dependent on the structural modifications of the cholinium cation (number of hydroxyethyl groups, alkyl chain length, etc.) [8]. Choline-based ILs have a broad spectrum of antimicrobial activity [9,10,11]. It has been shown [1,12] that choline-based ILs with geranate anions are highly effective against a wide range of viral, fungal and bacterial species and are harmless to human cells.

ILs have the potential to be used in the development of novel approaches to cancer treatment because they have much less cytotoxicity in healthy cells compared to cancer cells [13]. The IL-based paclitaxel preparation exhibited comparable antitumor activity against HeLa cells with significantly lower toxicity compared to Taxol [14,15].

Another approach to the development of biocompatible ILs is based on the use of components (cations, anions) of biological buffers (Good’s buffers) [13,16]. Buffers used in biochemical and biological studies must meet a number of requirements, including chemical inertness, high water solubility, low toxicity, availability, etc. [13,17]. ILs having self-buffering properties in the physiological pH range and obtained from Good’s buffers are known as Good’s buffer ionic liquids (GB-ILs) [18,19,20]. GB-ILs were found to exhibit a pronounced stabilizing effect on the structure of proteins [16,19]. GB-ILs can also be used to extract biomolecules (antibody, protein, etc.) [16,18,19,21].

We have recently developed GB-ILs based on hydroxyalkylammonium cations (triethanolammonium (TEA)) [22], tris(hydroxymethyl)methyl ammonium (TRIS) [22], diethanolammonium (DEA) [23], tris(2-hydroxypropyl)ammonium (TPA) [24], bis(2-hydroxyethyl)-tris(hydroxymethyl)methyl ammonium (BIS-TRIS) [25] and *N*,*N*,*N*′,*N*′-tetrakis(2-hydroxyethyl)ethylene diammonium (THEED) [26]) and biologically active carboxylic acids, which proved to be effective buffer agents in ^68^Ga-radiolabeling of bifunctional chelating agents (BCAs) and peptides. Today, ^68^Ga-based radiopharmaceuticals with receptor-targeted peptides have a great clinical impact, especially in PET diagnosis of prostate cancer and neuroendocrine tumors [27,28,29,30]. As is known, the formation of the ^68^Ga-chelator complex is possible only in a certain pH range [31]. In this regard, a buffer solution is used to obtain ^68^Ga-labeled radiopharmaceuticals with high radiochemical conversion (RCC). The 2-[4-(2-hydroxyethyl)piperazin-1-yl]ethanesulfonic acid (or HEPES) is a zwitterionic organic buffering agent widely used in the synthesis of ^68^Ga-radiopharmaceuticals [32,33,34]. Despite the excellent buffering properties of HEPES, its low limit (200 μg for parenteral administration for ^68^Ga-DOTA-TOC or 500 μg for ^68^Ga-PSMA-11) prescribed by the European Pharmacopoeia [35] precludes the use of HEPES in gallium-68 radiopharmaceuticals. This leads to the necessary additional step of purification of the ^68^Ga-labeled radiopharmaceutical from HEPES impurities prior to in vivo administration.

Alkanolammonium ILs are a promising alternative to zwitterionic buffer HEPES in ^68^Ga-radiolabeling reactions. We have previously shown that alkanolammonium ILs are significantly more efficient under the low-temperature ^68^Ga-radiolabeling protocol (37 °C) compared to the HEPES buffer [36]. This is a significant advantage, since some biomolecules (such as proteins) are not resistant to heating up to 90 °C. In addition, alkanolammonium ILs, in contrast to HEPES, are effective as buffers in Al^18^F-radiolabeling reactions [37].

It should be noted that some alkanolammonium ILs are drugs with immunomodulatory and adaptogenic properties and are widely used in medicine, pharmaceuticals, biotechnology and agriculture [38,39,40,41,42,43,44,45].

The aim of this work was to obtain new potential alkanolammonium buffer agents for ^68^Ga- and Al^18^F-radiolabeling reactions based on *N*-benzylethanolammonium cations and carboxylic acid anions. *N*-benzylethanolamine, C_6_H_5_CH_2_NHCH_2_CH_2_OH (BEA), is a hydroxyalkylamine containing one hydroxyethyl and one benzyl group. We have previously investigated the crystal structure and buffering properties of DEA-based GB-ILs [23]. Here we investigate the effect of substitution of one hydroxyethyl group for a benzyl group in the DEA cation on the structural features and buffering activity of BEA salts in radiolabeling reactions.

## 2. Materials and Methods

### 2.1. Materials

Reactants were purchased from “Vekton” (Russia) (succinic, malonic, oxalic, salicylic, cinnamic and benzoic acids (all ≥ 98%)); Sigma-Aldrich (*N*-benzylethanolamine, 95%; 1,2,4,5-benzenetetracarboxylic (pyromellitic acid), 96%; phthalic, 98%; 2-methylphenoxyacetic 99%; and 4-chloro-2-methylphenoxyacetic, ≥95% acids); Merck (Germany) (acetone, ethanol, acetonitrile, dimethylsulfoxide (DMSO), isopropyl alcohol); Macrocyclycs (USA) (*p*-SCN-Bn-DOTA, *p*-SCN-Bn-NOTA, *p*-SCN-Bn-DTPA, *p*-SCN-Bn-DFO, maleimodo-mono-amide-NOTA chelators); Chematech (France) (*p*-NCS-Bz-DOTA-GA, *p*-NCS-Bz-NODA-GA, NCS-MP-NODA, NOTA-NHS, NH_2_-MPPA-NODA chelators); Pharmsintez.Lab (Russia) (HBED-CC chelator, DOTA-TATE and DOTA-NOC peptides); Bachem (Switzerland) (DOTA-JR11 peptide); ABX (Germany) (PSMA-617, DOTA-AMBA, NODAGA-AMBA, NOTA-AMBA, NOTA-Octreotide peptides) and used as supplied.

### 2.2. Synthesis

BEA salt **1**. Benzoic acid (3.68 mmol, 0.450 g) dissolved in MeOH (20 mL) was added dropwise to a MEOH (5 mL) solution of BEA (3.68 mmol, 0.557 g). The mixture was heated at reflux for 2 h. After completion of the reaction, the solvent was evaporated under reduced pressure. The reaction product was isolated as a powder, washed with diethyl ether, and dried under reduced pressure. Yield: 87% (0.872 g).

**Scheme 1:**
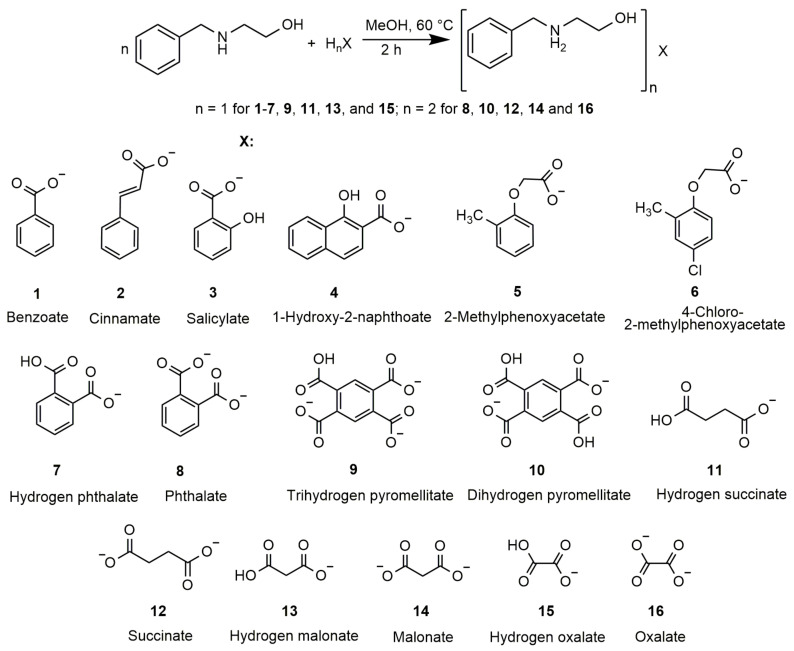
Synthesis of BEA salts indicating compound numbering **1–16**.

The BEA salts **2**–**16** (Scheme 1) were synthesized similarly to compound **1** with a molar ratio of BEA to carboxylic acid equal to 1:1 (salts **2**–**7**, **9**, **11**, **13**, and **15**) and 2:1 (salts **8**, **10**, **12**, **14**, and **16**). Some of the experimental data for BEA salts **1**–**16** are presented in more detail in Table 1. Crystals of BEA salts **11**, **15** and **16** for X-ray diffraction were isolated from a water/methanol mixture by slow evaporation at room temperature.

### 2.3. Methods

The ATR-FTIR spectra (Figures S1–S16) of BEA salts **1**–**16** were recorded on the FTIR spectrometer InfraLUM FT-08 in the spectral region of 4000–500 cm^−1^. NMR (^1^H and ^13^C) spectra (Figures S17–S31) of BEA compounds in D_2_O were registered on a BrukerBioSpin AG Avance III HD 400. C, H and N elemental analyses were carried out on a Euro EA3028-NT analyzer. Thermogravimetric curves of solid BEA salts **1**, **2**, **7**–**12**, **15** and **16** were recorded on a Shimadzu DTG-60 derivatograph using aluminum crucibles. The samples were heated at a rate of 10 °C/min under an air in the temperature range from 40 to 300 °C.

### 2.4. X-ray Structure Determination

Data for BEA salts **11**, **15**, and **16** were collected on a Rigaku Oxford Diffraction «Rigaku «XtaLAB Synergy» diffractometer using monochromated CuKα radiation. Experimental details are shown in Table 2. Structures were solved by direct methods and refined by full-matrix least-squares methods with the SHELXL program [46] as incorporated into the OLEX2 program package [47]. The carbon- and nitrogen-bound H atoms were placed in calculated positions and were included in the refinement in the riding model approximation with U_iso_(H) set to 1.2U_eq_(C) and C–H 0.95 Å for the CH groups, with U_iso_(H) set to 1.2U_eq_(C) and C–H 0.99 Å for the CH_2_ groups and with U_iso_(H) set to 1.2U_eq_(N) and N–H 0.91 Å for the NH_2_ groups. The oxygen-bound H atoms were located in the difference Fourier map. Empirical absorption correction was applied in the CrysAlisPro program complex (CrysAlisPro, Agilent Technologies, Yarnton, UK. Version 1.171.36.32). Supplementary crystallographic data have been deposited at Cambridge Crystallographic Data Centre (2,236,100 for **11**, 2,236,099 for **15** and 2,236,098 for **16**) and can be obtained free of charge via www.ccdc.cam.ac.uk/data_request/cif (accessed on 13 January 2023).

### 2.5. Hirshfeld Surfaces Analysis

Hirshfeld surface analyses and their associated fingerprint plots for BEA salts **11**, **15** and **16** were generated based on a CIF file using the CrystalExplorer 17.5 program(University of Western Australia) [48] to visualize intermolecular interactions. The d_i_ and d_e_ represent the interior atom to the Hirshfeld surface and the exterior atom to the Hirshfeld surface. The colors on the Hirschfeld surface correspond to the contact distances (red—close, white—medium, blue—long) between atoms on both sides of the surface.

### 2.6. ADME Analysis

The SwissADME web tool provided by the Swiss Institute of Bioinformatics, Lausanne, Switzerland [49] was used to predict the physicochemical and pharmacological properties of BEA salts **1–16**.

### 2.7. Buffer Activity

#### 2.7.1. Preparation of ^68^Ga

The isotope gallium-68 in the form of [^68^Ga]Ga^3+^ was obtained from the 75 mCi ^68^Ge/^68^Ga generator (Cyclotron Ltd., Obninsk, Russia). The elution of activity from the generator was performed with 5 mL of 0.1 M HCl and 0.3–0.5 mL fractions were collected. Fractions containing highest amount of activity were combined and diluted with high-purity 0.1 M HCl to reach volume activity 30–50 MBq/mL, and the resulting solution was used as stock solution.

#### 2.7.2. Preparation of ^18^F

The fluorine-18 isotope in the form of [^18^F]F^−^ was obtained by the nuclear reaction ^18^O(p,n)^18^F and realized by irradiation of ^18^O-enriched water (97% enrichment by ^18^O, CJSC “Global Scientific Technology”, Sosnovy Bor, Russia) with 16.4 MeV protons in an aqueous target (volume 2.4 mL, Nb target) of the PETtrace 4 cyclotron (GE Healthcare, Uppsala, Sweden). After the irradiation was complete, the target material was collected in the glass vial and used as stock solution.

#### 2.7.3. General Method of High-Temperature Radiolabeling of BCAs and Peptides with ^68^Ga (HT Radiolabeling)

1.8–14.4. nmol of BCAs or peptide was sequentially loaded into a 0.5 mL Eppendorf-type PP tube. Next, 25 µL of 0.1–1 M aqueous solution of BEA salts **1–16**, 37.5 µL of EtOH and 125 µL of ^68^GaCl_3_ solution in 0.1 M HCl were sequentially added to the test tube. The reaction was carried out by stirring in a thermo-shaker (TS-100C, Biosan, Latvia) for 10 min and heating to 95 °C. After cooling RCCs were measured by radio-TLC (iTLC-SG strips; eluent—50% acetonitrile in water; R_f_ 0.6–0.8 for radiolabeled peptides and 0.0 for [^68^Ga]Ga^3+^ and ^68^Ga in colloidal form).

#### 2.7.4. General Method of Low-Temperature Radiolabeling of BCAs and Peptides with ^68^Ga (LT Radiolabeling)

Briefly, 1.8–14.4. nmol of BCAs or peptide was sequentially loaded into a 0.5 mL Eppendorf-type PP tube. Next, 25 µL of 0.1–1 M aqueous solution of BEA salts **1–16**, 37.5 µL of acetone and 125 µL of ^68^GaCl_3_ solution in 0.1 M HCl were sequentially added to the test tube. The reaction was carried out by stirring in a thermo-shaker (TS-100C, Biosan, Latvia) for 10–30 min and heating to 37 °C. After cooling, RCCs were measured by radio-TLC (iTLC-SG strips; eluent—50% acetonitrile in water; R_f_ 0.6–0.8 for radiolabeled peptides and 0.0 for [^68^Ga]Ga^3+^ and ^68^Ga in colloidal form).

#### 2.7.5. General Method of Radiolabeling of BCAs and Peptides with ^18^F

Briefly, 3µ. L of 2 mM AlCl_3_ solution (pH 4), 100 µL of 0.05 M aqueous solution of BEA salts 1–16, 100 µL of DMSO and 125 µL of ^68^GaCl_3_ solution in 0.1 M HCl were sequentially added to the 0.5 mL Eppendorf-type PP tube. After 3 min, 23.5 nmol of BCAs or peptide was added to the test tube. The reaction was carried out by stirring in a thermo-shaker (TS-100C, Biosan, Latvia) for 20 min and heating to 50–100 °C. After cooling, RCCs were measured by radio-TLC. (Sorbfil HPTLC-AF-UV strips; eluent—1M NH_4_Ac/MeOH = 1:1; R_f_ 0.6–0.8 for radiolabeled peptides and 0.0 for [^18^F]F^−^ and [Al^18^F]^2+^).

#### 2.7.6. Statistical Analysis

All experiments were carried out at least in triplicate. All data are expressed as the mean of values ± standard deviation (mean ± SD). Prism 5 software (GraphPad Software, San Diego, CA, USA) was used to determine statistical significance at the 95% confidence level, with a *p* value less than 0.05 being considered significant.

#### 2.7.7. pH Profiles

The pH titration profiles of was determined in a glass vessel using a pH meter (SevenCompactS210, Mettler Toledo, Columbus, OH, USA) equipped with a pH glass combined electrode (InLab Ultra-Micro-ISM, Mettler Toledo, Columbus, OH, USA) for pH measurements in aqueous solutions. The pH electrode was calibrated in aqueous solution with three standard buffers of pH 4.01, 7.00 and 9.21. The temperature of the titration vessel was controlled at 20 °C using a thermostatic water bath. For measuring the pH profile 5 mL of 0.01 M solution was freshly prepared in water and titrated with 0.01 M NaOH/HCl under continuous magnetic stirring. At least two repeated measurements were performed for the determination of pH profile.

## 3. Results and Discussion

We describe the synthesis of sixteen new *N*-benzylethanolamine compounds derived from the proton transfer from a carboxylic acid to a hydroxyalkylamine. All synthesized BEA salts were characterized by spectroscopic methods (FTIR, NMR) and elemental and thermal analysis. The conformation of BEA cations and intermolecular interactions in three BEA salts with hydrogen succinate, hydrogen oxalate and oxalate anions were investigated using X-ray diffraction and Hirshfeld surfaces analysis. In silico ADME analysis was used to predict the drug-likeness features of new BEA-based ionic compounds. Furthermore, newly synthesized BEA carboxylates were tested as buffer agents in radiolabeling reactions with isotopes ^68^Ga and ^18^F (via [Al^18^F]^2+^ motif).

### 3.1. Synthesis and Characterization

*N*-benzylethanolammonium salts **1**–**16** were synthesized by reacting BEA with biologically active carboxylic acids according to Scheme 1 and isolated as powders or viscous oily liquids (Table 1). The formation of new compounds was confirmed by IR spectroscopy, NMR (^1^H, ^13^C) and elemental analysis.

All FTIR spectra of BEA salts **1**–**16** (Figures S1–S16) exhibited a broad intense bands with maxima in the region of 3445–3115 cm^−1^ assigned to O-H vibrational stretching of BEA cations. The spectral region of 3060–2720 cm^−1^ is characterized notably by the stretching vibrations ν(C–H) groups attributed to the aliphatic symmetric and asymmetric stretching C–H modes (3000–2720 cm^−1^), while the aromatic C_Ar_–H groups oscillate at a higher frequency (≥3000 cm^−1^) [50]. Stretching vibrations of the ammonium group ν(N^+^H_2_) appear as a set of bands in the region of 2680–2350 cm^−1^. Carboxylate anions display two characteristic strong and medium bands due to antisymmetric ν_as_(COO^−^) and symmetric ν_s_(COO^−^) stretching vibrations in the spectral region of 1624–1530 cm^−1^ and 1420–1323 cm^−1^, respectively. Noticeable bands in the region 1685–1410 cm^−1^ belong to carbon-carbon stretching vibrations in the aromatic ring from BEA cations and some anions (salts **1**–**10**). The formation of acid salts in the case of compounds **7**, **9**–**11**, **13** and **15** was confirmed by the presence of ν(COOH) bands in the spectral region of 1710–1700 cm^−1^. Some FTIR spectroscopy data for BEA salts **1**–**16** are summarized in Table 3.

In the ^1^H NMR spectra (Figures S17a–S31a) of BEA salts **1**–**16**, the proton signal of the hydroxyethyl branch NCH_2_CH_2_OH appears as two multiplets in the region of 3.03–3.28 and 3.68–3.92 ppm. Singlet signals of CH_2_ group from benzyl fragment of the BEA cations are observed at 4.19–4.28 ppm. Aromatic protons from BEA cations and anions **1**–**10** show multiplet signals in the region of 6.60–8.04 ppm. ^13^C NMR spectra of **1**–**16** (Figures S17b–S31b) are characterized by signals in the region 48.4–56.8 ppm, assigned to CH_2_ carbons of the hydroxyethyl and benzyl groups of BEA cations. A set of peaks at 111.5–159.5 ppm belong to aromatic carbon region. The carbons of the COO^−^ (and COOH) groups appear at 165.5–179.6 ppm.

### 3.2. Thermogravimetric Analysis

**Figure 1:**
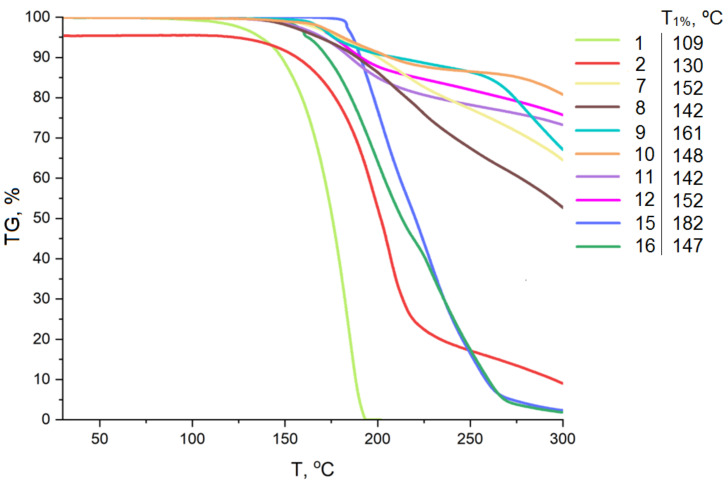
Thermogravimetric curves of BEA salts **1**, **2**, **7**–**12**, **15** and **16**.

Figure 1 shows the thermogravimetric (TG) curves of BEA salts **1**, **2**, **7**–**12**, **15** and **16** isolated in solid form. The temperature corresponding to 1% weight loss was taken as the start of thermal decomposition. According to the results obtained, the thermal decomposition of BEA salts starts in the temperature range of 109–182 °C. Compounds **2**, **7**, **8**, **11** and **12** melt in the temperature range of 91–113 °C before the onset of weight loss. For other BEA salts, the melting process occurs either simultaneously with decomposition (salts **1**, **15**) or after it (salts **9**, **10** and **16**). The thermal decomposition of BEA salts **1**, **2**, **15** and **16** is quite similar and is a one-step process with a high mass loss rate. Weight loss at 300 °C was almost 100%. For other BEA salts, the decomposition process turned out to be slower. On the TG curves of BEA salts of pyromellitic acid **9** and **10**, several noticeable stages of weight loss can be seen. In general, the thermal decomposition temperatures of the BEA salts are in good agreement with the data for the hydroxyalkylammonium salts with analogous anions [23,24,25,26]. Compared with DEA salts [23], the replacement of one hydroxyethyl group in the cation with a more rigid benzyl fragment led to a significant increase in the melting point and, as a result, to the transition from room-temperature ILs to solids at room temperature (with the exception of liquid salts **3**–**6** and **11**).

Thus, in the BEA salts series, protic ILs with melting points <100 °C (compounds **7**, **8**, **11** and **12**), room-temperature ILs (compounds **3**–**6**, **13** and **14**) and protic molten salts with melting points <200 °C (compounds **1**, **2**, **9**,**10**, **15** and **16**) were identified.

### 3.3. X-ray Crystal Structure

**Figure 2:**
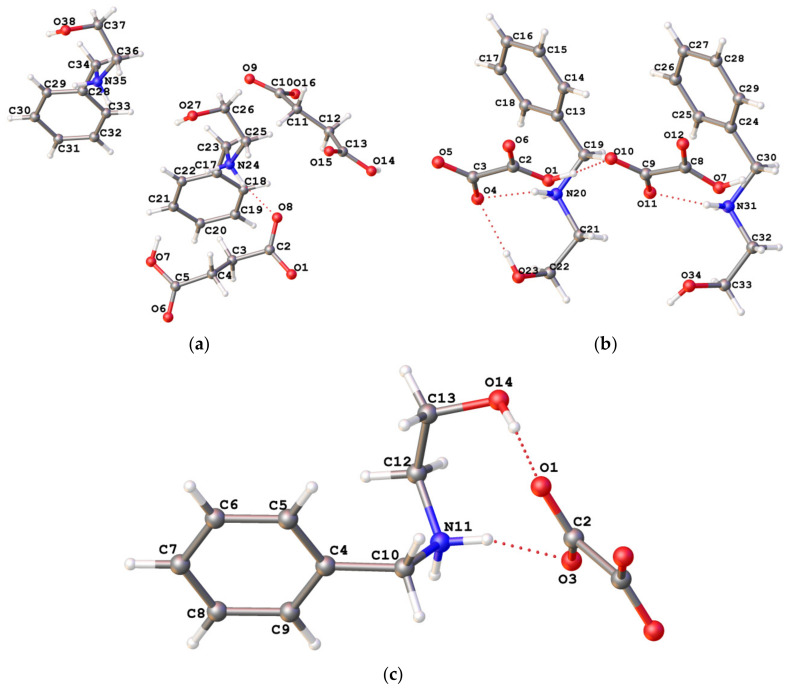
X-Ray structure of BEA salts (**a**) **11**; (**b**) **15** and (**c**) **16**.

**Figure 3:**
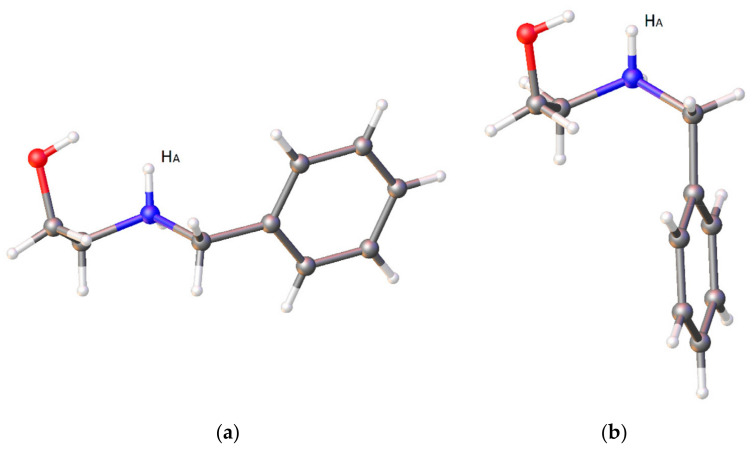
Two different cation conformations in BEA salts **11** (**a**) and **16** (**b**).

The crystal structures of three BEA salts **11**, **15** and **16** were determined by X-ray diffraction. The unit cells of BEA salts **11** and **15** contain two cations and two anions: hydrogen succinate or hydrogen oxalate, respectively (Figure 2a,b). The unit cell of compound **16** contains one cation/anion pair (Figure 2c). In all BEA cations, the hydroxyethyl group is directed to one of the ammonium hydrogen atoms, as in the case of the typical endo-conformation of the hydroxyalkylammonium cation [23,24,25,26]. A significant difference between the BEA cations in salts **11**, **15** and **16** was the position of the benzyl moiety relative to the ammonium group. In compounds **11** and **15**, the benzyl group is in the plane of the carbon and nitrogen atoms of the –CH_2_NH_2_CH_2_– fragment (Figure 3a). H_A_NCC torsion angles vary in the range of 41.9–70.5°. Torsion H_A_NCC in BEA salt **16** was 172.3°. The benzyl group is in the trans position and is significantly deviated from the plane of the nitrogen and carbon atoms of the hydroxyethyl group in the opposite direction from the ammonium group (Figure 3b).

**Figure 4:**
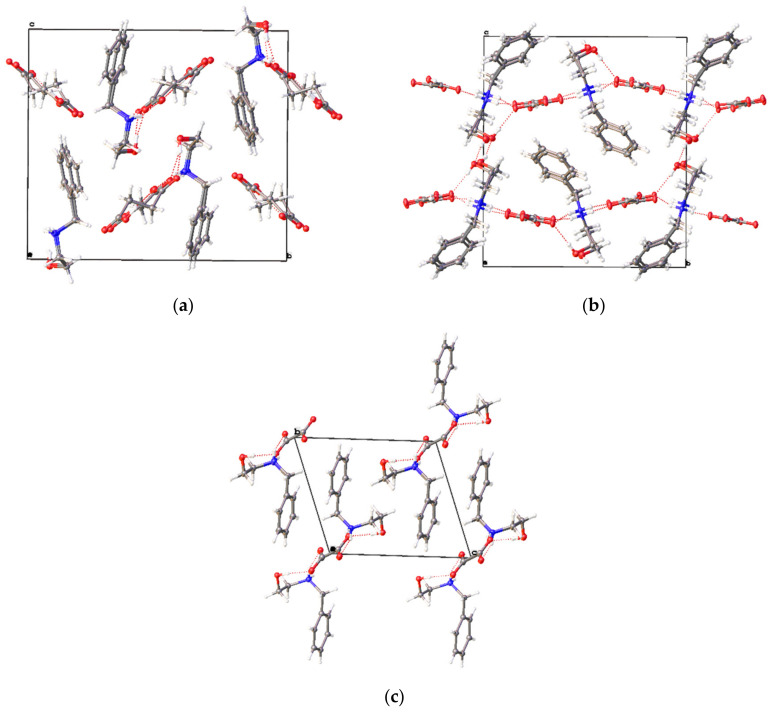
Packing diagram for BEA salts **11** (**a**), **15** (**b**) and **16** (**c**). Dashed lines indicate intermolecular interactions.

A fragment of the crystal structure of BEA salts **11**, **15** and **16** with intermolecular contacts is shown in Figure 4. In BEA salt **11**, each cation forms three hydrogen bonds with the COO^−^ groups of the hydrogen succinate anion using the ammonium (N_24_H_A_···O_9_ and N_24_H_B_···O_8_; N_35_H_A_···O_1_ and N_35_H_B_···O_16_) and hydroxyethyl group (O_27_H···O_16_ and O_38_H···O_8_). Hydrogen succinate anions are arranged in chains along the a-axis due to hydrogen bonds O_7_H···O_9_ and O_14_H···O_1_. Similar hydrogen-bonded chains of carboxylic acid anions –COOH···OOC– were observed in the crystal structures of TEA hydrogen succinate, hydrogen malonate and hydrogen oxalate [51]. A similar picture of intermolecular interactions is also observed for BEA hydrogen oxalate **15**. Hydrogen oxalate anions also form anionic chains due to contacts: O_1_H···O_10_ and O_7_H···O_5_. In contrast to the crystal structure of **11**, additional contacts involving the hydroxyl groups of BEA cations are observed in structure **15**. In particular, the hydroxyethyl group acts as a H-bond donor due to contact with the hydrogen oxalate anion (O_23_H···O_4_) and a H-bond acceptor due to interaction with the OH group of the neighboring BEA cation (O_34_H⋅⋅⋅O_23_). Such hydrogen bonding leads to the formation of cationic dimers from BEA cations arranged in columns along the a-axis (Figure 4b).

In BEA oxalate **16** (Figure 4c), as in the crystal structure **11**, cations are H-bonded only to anions through three contacts: N_11_H_A_⋅⋅⋅O_3_; N_11_H_B_⋅⋅⋅O_1_ and O_14_H⋅⋅⋅O_1_. The main difference is that the formation of anionic chains is not observed due to the absence of a free –COOH group. The hydrogen bonding parameters in the crystal structures of BEA salts **11**, **15** and **16** are presented in Table 4.

### 3.4. Hirshfeld Surface Analysis

**Figure 5:**
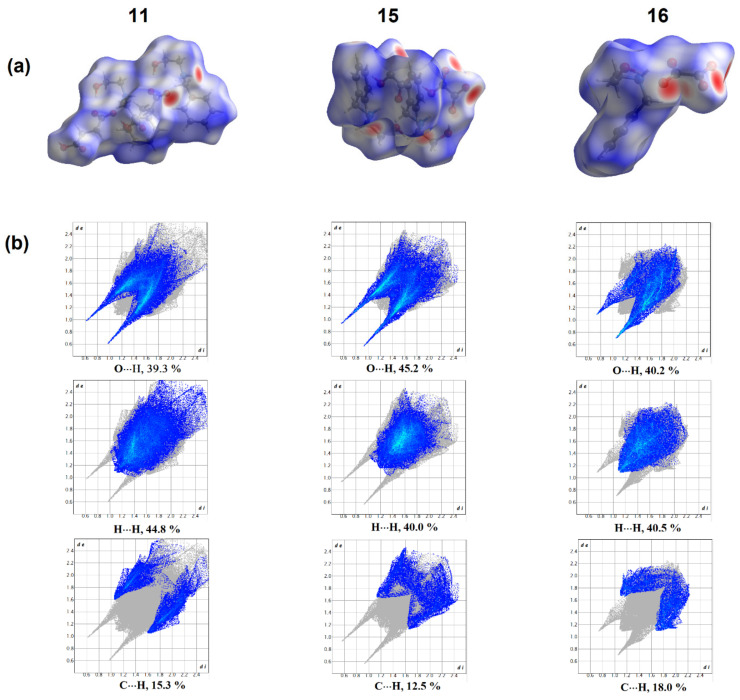
The three-dimensional Hirshfeld surface for BEA salts **11**, **15** and **16** plotted over d_norm_ (**a**); two-dimensional fingerprint plots for all intermolecular contacts in BEA salts **11**, **15** and **16** (**b**).

The intermolecular interactions in crystal structures **11**, **15** and **16** have been additionally investigated and visualized by Hirshfeld surface analysis. 3D Hirshfeld surface maps and 2D fingerprint plots were generated and presented in Figure 5. The red spots on the d_norm_ surface appear as a result of short interatomic contacts, while the other weak intermolecular interactions emerge as light red, white and blue spots. In all cases, the red spots (Figure 5a) correspond to hydrogen bonds involving carboxylate oxygen atoms of anions: N-H···O and O-H···O (Table 4).

As shown in Figure 5b, in the case of BEA salt **11**, the H···H interactions make a 44.8% contribution to the total Hirshfeld surface and are the most significant interactions. In BEA salt **15** interactions O⋅⋅⋅H/H⋅⋅⋅O ccupies most important place with a 45.2% contribution to the total Hirshfeld surface. This distinction can be explained by the difference in the anionic fragments of salts **11** and **15**, namely, the absence of two CH_2_ groups in BEA salt **15** compared to salt **11**. Unlike salts **11** and **15**, in salt **16** [NH_2_(CH_2_CH_2_OH)(CH_2_C_6_H_5_)]_2_(O_2_C-CO_2_), each oxalate anion is hydrogen-bonded with two [BEAH]^+^ cations. Here, almost the same contribution of the O⋅⋅⋅H/H⋅⋅⋅O and H···H interactions is observed, equal to 40.2 and 40.5%, respectively, to the total Hirshfeld surface. The benzyl group present in all BEA cations leads to a significant increase in the contribution of C⋅⋅⋅H/H⋅⋅⋅C interactions to the total surface compared to hydroxyalkylammonium salts that do not contain aromatic groups in cations and anions [26].

### 3.5. In Silico ADME Prediction

The ADME analysis was applied to evaluate the drug-likeness properties of new ILs based on BEA salts **1**–**16**. For comparison, two biologically active compounds trekrezan and chlorcrezacin [38,39,40,41,42,43], which are TEA 2-methylphenoxyacetate 2-methyl-4-chlorophenoxyacetate, respectively, were used. In silico, some ADME physicochemical properties were predicted and presented in Table 5. Lipinski’s rule of five was used to evaluate the potential oral bioavailability of BEA salts **1**–**16**. According to this rule, good absorption or permeation is more likely when the molecular weight (M_W_) < 500 Da, number of H-bond donors <5, number of H-bond acceptors <10 and an octanol/water partition coefficient (Log *P*) < 5. The compounds with no more than one violation are considered as the drug-likeness. From Table 5, all listed BEA salts (except BEA salt of pyromellitic acid **10**) are satisfied by Lipinski rule with zero violations. All calculated physicochemical properties for BEA salts (except compound **10**) are within the expected thresholds (M_w_ = 241–469; Log *P*: from −1.9 to +1.2; HB acceptors = 4–9 and HB donors = 2–5). In addition, BEA salts **2**–**7** and **11** are recognized the high gastrointestinal absorption. The lipophilicity, determined by an octanol/water partition coefficient (log *P*) is closely associated with transport processes, including membrane permeability, and distribution to different organs and tissues [52]. A general guidance for sufficient oral bioavailability (good solubility and permeability) is to have a temperate log *P* (0 < log *P* < 3) [53]. All tested ILs **1**–**16** maintains the log *P* values that are in the acceptable range according to Lipinski’s rule (Table 5).

Thus, fifteen of the sixteen tested BEA salts did not violate any of the Lipinski’s rules of five and were expected to be orally active. For most compounds, the bioavailability score was >0.5. The predicted parameters turned out to be close to known drugs (trekrezan, chlorcrezacin) with immunomodulatory and adaptogenic activity, which confirms their potential drug-likeness. The predicted drug-likeness for BEA salts turned out to be much more positive than for THEED-based ILs, whose cations contain two ammonium and four hydroxyethyl groups [26].

### 3.6. Buffer Activity in ^68^Ga-Radiolabeling Reactions

The screening of BEA carboxylates as potential buffer solutions for ^68^Ga-radiolabeling was carried out using the example of a reaction with a model BCA *p*-SCN-Bn-DOTA. DOTA as a model BCA was chosen because it is one of the most common chelating groups included in the structure of many clinically significant radiopharmaceuticals [54]. Furthermore, it is common knowledge that DOTA makes stable complexes with many isotopes in addition to gallium-68 and provides stability of bioconjugates in vitro and in vivo. The full evaluation was carried out with using of high-temperature (HT) and low-temperature (LT) radiolabeling protocols [23,25,26,36].

**Figure 6:**
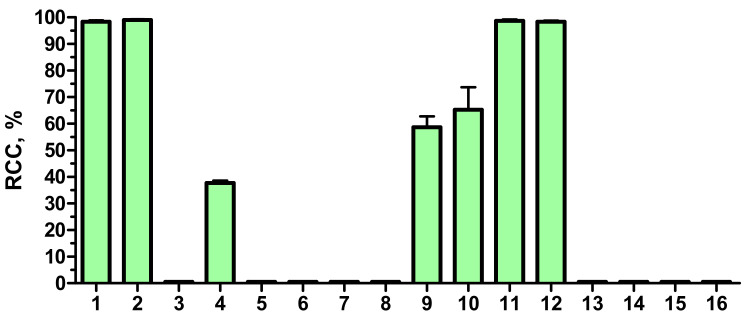
High-temperature ^68^Ga-radiolabeling of *p*-SCN-Bn-DOTA in a media of BEA salts **1**–**16** (95 °C, 10 min).

As expected (Figure 6), ^68^Ga-DOTA complex formation does not occur in BEA salts of oxalic and malonic acids **13**–**16** under HT-radiolabeling due to the tendency of the anions to interact with ^68^Ga itself. Surprisingly, a similar result was found for salts **5** and **6**. In our previous works [23,24,25,26,36], ILs containing 2-methylphenoxyacetate and 4-chloro-2-methylphenoxyacetate anions as buffering agents provide almost quantitative RCCs for the reaction of ^68^Ga radioisotope with the DOTA chelator. BEA salts **4**, **9** and **10** showed average RCCs in the 40–65% range. As a result, the most effective buffers in ^68^Ga-radiolabeling reactions were **1**, **2**, **11** and **12**, which provided RCCs over 98%. Thus, these four BEA-based ILs were chosen for further radiochemical experiments. 

**Figure 7:**
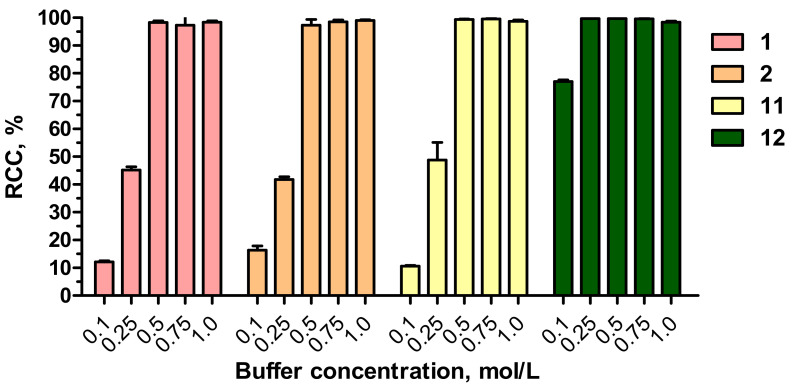
High-temperature ^68^Ga-radiolabeling of *p*-SCN-Bn-DOTA under different concentrations of BEA salts **1**, **2**, **11** and **12** (95 °C, 10 min).

In all previous experiments with monocationic hydroxyalkylammonium ILs, the optimal concentration of the buffer solution was 1 M [22]. Here, we studied ^68^Ga-radiolabeling reactions at different concentrations of BEA-based ILs: 0.1, 0.25, 0.5, and 0.75 M (Figure 7). At a buffer concentration of 0.1 M, the reaction efficiency was low (<20%) for all buffers except BEA salt **12** with a RCC of 77%. With increasing buffer concentration, efficiency gains for ^68^Ga-radiolabeling were observed. At concentration of 0.25 M, RCC > 99% was achieved only in the BEA succinate **12**. For the other three buffers, the RCCs varied from 40 to 50% at the same concentration. When using 0.5 M buffer solutions, maximum efficiency was achieved for all BEA-based ILs. This result is unexpected because for monocationic ILs (TEA, DEA, TPA, BIS-TRIS salts) high RCCs for ^68^Ga-radiolabeling reactions were only achieved at concentrations ≥1 M. However, it should be noted that for dicationic ILs (THEED carboxylates [26]), high RCCs can also be observed at a buffer concentration of 0.5 M.

**Figure 8:**
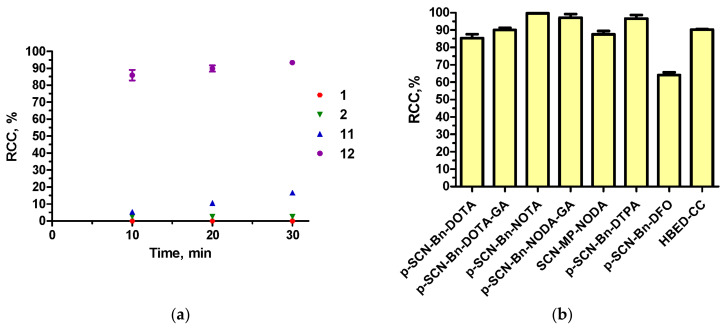
Effect of reaction time on low-temperature radiolabeling of *p*-SCN-Bn-DOTA in a media of buffers **1**, **2**, **11** and **12** (37 °C, 10–30 min) (**a**); low-temperature radiolabeling of cyclic and acyclic BCAs in a media of buffer **12** (37 °C, 10 min) (**b**).

At the next stage, the efficiency of ^68^Ga-radiolabeling of the *p*-SCN-Bn-DOTA chelator was evaluated at 37 °C (LT-radiolabeling) as a function of reaction time. For ^68^Ga-radiolabeling, the optimal incubation time is 10–15 min. It can be noted (Figure 8a) that in all cases (buffers **1**, **2**, **11** and **12**), an increase in the reaction time leads to a slight increase in efficiency at 37 °C. Surprisingly, BEA buffers **1** and **2** demonstrated almost zero efficiencies at any reaction time. In the buffer **11**, an increase in RCCs values was observed from 5% at 10 min to 17% at 30 min. Among the tested buffers, BEA succinate **12** demonstrated the highest efficiency under the LT protocol, which was 85% at 10 min and 92% at 30 min of reaction. The effectiveness of IL **12**, as the most promising BEA buffer, was also confirmed in ^68^Ga-radiolabeling reactions with various cyclic and acyclic BCAs under LT conditions (Figure 8b). For all chelators, the RCCs were above 85% with the exception of *p*-SCN-Bn-DFO. The RCC of radiolabeling of the *p*-SCN-Bn-DFO chelator was found to be 64%, which correlates well with the results obtained for DEA- and THEED-based ILs [23,26].

**Figure 9:**
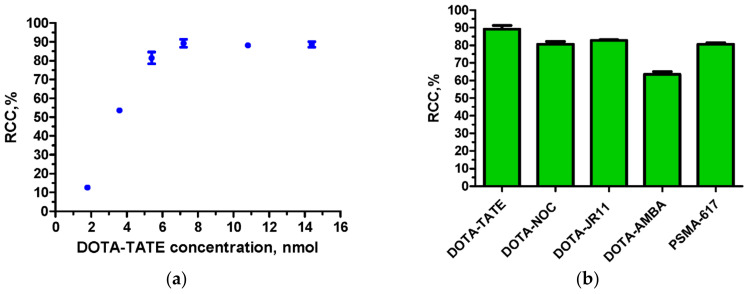
Optimization of ^68^Ga-radiolabeling of DOTA-TATE peptide in a media of buffer **12** (37 °C, 10 min) (**a**); LT radiolabeling of clinically relevant peptides in a media of buffer **12** (37 °C, 10 min) (**b**).

After the model reactions with BCAs, buffer **12** was screened in reactions with peptides that are widely used in the synthesis of ^68^Ga-labeled radiopharmaceuticals. It should be noted that all reactions were carried out using the LT-radiolabeling protocol. Figure 9a shows the dependence of the RCC values on the concentration of the DOTA-TATE peptide chosen as a model. The results indicate that the maximum efficiency of ^68^Ga-radiolabeling was achieved with a peptide amount of 7.2 nmol. An additional increase in the amount of the peptide did not lead to an increase in the RCC values. Further, after determining the optimal peptide concentration, the effectiveness of buffer **12** was tested in reactions with other clinically significant peptides: DOTA-NOC, DOTA-JR11, DOTA-AMBA and PSMA-617 (Figure 9b). High efficiency (RCCs > 80%) was achieved for most of the tested peptides. The only exception was the DOTA-AMBA peptide (RCC ~64%).

### 3.7. Buffer Activity in Al^18^F-Radiolabeling Reactions

One of the essential points in the formation of complexes with aluminum fluoride ([Al^18^F]^2+^ cation) is also the choice of a suitable BCA which is stable for several hours in biological media. Since aluminum preferentially forms octahedral complexes, pentadentate ligands (usually N_3_O_2_) are predominantly used. Due to this, the free binding site is occupied by the fluoride ion [55,56].

**Figure 10:**
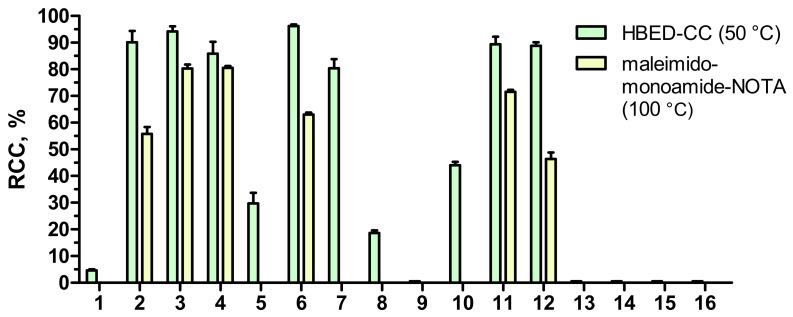
Al^18^F-radiolabeling of HBED-CC and maleimido-monoamide-NOTA chelators in a media of BEA salts **1**–**16** (50–100 °C, 20 min).

Based on previous results [37], here we studied the ability of the HBED-CC chelator to form complexes with the [Al^18^F]^2+^ cation in BEA-based buffers (Figure 10). By analogy with ^68^Ga-radiolabeling reactions, Al^18^F-radiolabeling of the HBED-CC chelator in BEA salts of malonic and oxalic acids **13**–**16** did not proceed (RCCs ≈ 0). Buffers **1** (RCC < 5%) and **5** (RCC 30%) showed extremely low efficiency, which is unusual for salts containing similar anions [37]. In the BEA salts of phthalic (**7**, **8**) and pyromellitic (**9**, **10**) acids, only buffer **7** showed high radiolabeling efficiency (80%). Six BEA salts: **2** (90%), **3** (94%), **4** (89%), **6** (96%), **11** (89%) and **12** (88%) turned out to be the most effective in Al^18^F-radiolabeling reactions and were selected for further screening. Six selected BEA-based buffers were tested in Al^18^F-radiolabeling reactions of the maleimido-monoamide-NOTA chelator (Figure 10). This chelator has an N_3_O_2_-configuration, which is most suitable for Al^18^F-radiolabeling reactions with cyclic BCAs. In contrast to the HBED-CC chelator, reactions were carried out at 100 °C [57]. Among the tested buffers, only BEA salts of salicylic (**3**) and 1-hydroxy-2-naphthoic (**4**) acids showed the highest efficiency (over 80%). The RCCs of Al^18^F-radiolabeling of the maleimido-monoamide-NOTA chelator in buffers **2**, **6**, **11** and **12** did not exceed 72%. The lowest efficiency of Al^18^F-radiolabeling was achieved in buffer **12**, which was the most optimal in the ^68^Ga-radiolabeling reactions.

**Figure 11:**
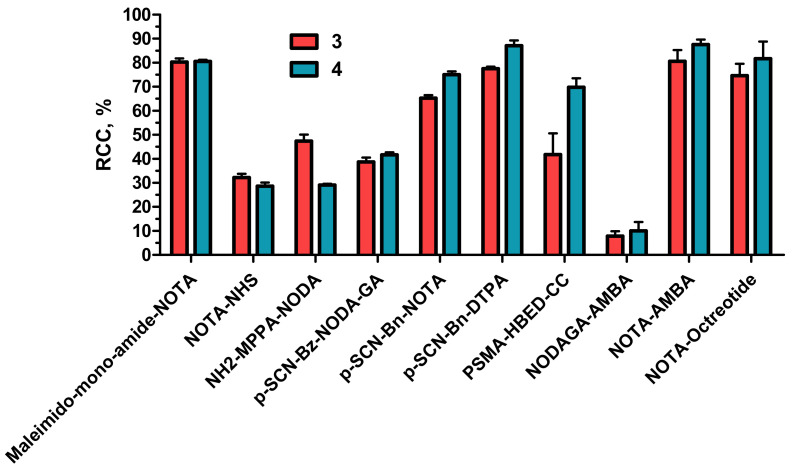
Al^18^F-radiolabeling of BCAs and clinically relevant peptides in BEA-based buffers **3** and **4** (50–100 °C, 20 min).

In the next step, BEA salts **3** and **4** were screened in Al^18^F-radiolabeling of a number of BCAs (Figure 11), which are structural fragments of clinically significant peptides. RCCs for Al^18^F-radiolabeling of NOTA-NHS and NH_2_-MPPA-NODA in buffer **3** were 32% and 47%, respectively. Buffer **4** showed approximately the same efficiency for these chelators, which was about 30%. NOTA-NHS and NH_2_-MPPA-NODA chelators, as well as maleimido-mono-amide-NOTA, have the N_3_O_2_ configuration. In this regard, it was expected that high RCCs would be achieved in Al^18^F-radiolabeling reactions of these chelators. Nevertheless, the efficiency of radiolabeling turned out to be low, which is probably due to the side processes of the interaction of the BCA molecule with ^18^F-fluoride. As expected, the radiolabeling efficiency of the hexadentate chelator *p*-SCN-Bz-NODA-GA chelator (N_3_O_3_ configuration) was low for both buffers (less than 45%). However, the reaction with the *p*-SCN-Bn-NOTA N_3_O_3_-chelator proceeded more efficiently (>60%). In addition, the reactions of the *p*-SCN-Bn-DTPA chelator with the N_3_O_4_ structure, which forms complexes with di- and trivalent cations and, in particular, with [Al^18^F]^2+^ [58], have been studied. In the radiolabeling reactions with *p*-SCN-Bn-DTPA, buffers **3** and **4** demonstrated the highest efficiency of 78% and 87%, respectively.

The Al^18^F-radiolabeling process for some clinically significant peptides has also been investigated in buffers **3** and **4** (Figure 11). Thus, the RCCs for complex Al^18^F-PSMA-HBED-CC (Al^18^F-PSMA-11) at 50 °C were 42% for **3** and 70% for **4**, which is slightly lower than for TEA- and TPA-based buffers [37]. The NODAGA-AMBA peptide, as a potential GRPR-agonist, has the N_3_O_3_-structure of BCA. Therefore, the low efficiency of the radiolabeling process was expected. The conversion at 100 °C was 8% for salt **3** and 10% for **4**, which is almost two-fold higher than the RCCs in a previously published study [59]. For another GRPR-agonist NOTA-AMBA with the N_3_O_2_ chelating fragment, the expected high yields were achieved (81% for **3** and 88% for **4**). Similarly, Al^18^F-NOTA-Octreotide complex, as one of the most promising ^18^F-labeled somatostatin analogs for PET diagnostics [60,61], was obtained with a high efficiency of over 75% in both BEA-based buffers. Thus, the developed technique of Al^18^F-radiolabeling can subsequently be adapted for the synthesis of a wide range of clinically significant peptides.

pH Profiles were measured for BEA salts **12**, **3** and **4**, which proved to be the most effective in the ^68^Ga- and Al^18^F-radiolabeling reactions. These results (Figures S32–S34) confirmed that these BEA-based ILs are capable of maintaining their buffer potential in an aqueous medium.

## 4. Conclusions

A series of sixteen new hydroxyalkylammonium salts containing *N*-benzylethanolammonium cations has been synthesized for the first time. It has been shown that BEA salts are alkanolammonium protic ionic liquids (M.P. < 100 °C) or protic molten salts (M.P. < 200 °C) with thermal stability in the range of 109–182 °C depending on the anion. Structural studies have shown that the BEA conformations of cations can differ depending on the location of the benzyl group relative to the ammonium fragment. In crystal packing, O⋅⋅⋅H/H⋅⋅⋅O and H···H interactions are the most important and contribute more than 39% to the total Hirshfeld surface. Hydrogen succinate and hydrogen oxalate anions form the strongest hydrogen bonds –COOH⋅⋅⋅OOC–, which leads to the creation of anionic chains. Due to the benzyl fragment in BEA cations, the contribution of C⋅⋅⋅H/H⋅⋅⋅C interactions to the total surface is significantly increased and varies in the range of 12–18%. In silico ADME prediction revealed that most of the synthesized BEA salts are potentially orally active with bioavailability score >0.5. It has been shown that BEA salts can be used as buffer agents in ^68^Ga- and Al^18^F-radiolabeling reactions of bifunctional chelating agents and peptides. Surprisingly, the high efficiency of ^68^Ga-radiolabeling was achieved at a buffer concentration of 0.5 M. Under low-temperature conditions among the tested buffers, BEA succinate demonstrated the highest radiolabeling efficiency. BEA salts of salicylic and 1-hydroxy-2-naphthoic acids proved to be the most effective buffers in the Al^18^F-radiolabeling reactions of chelating agents. Al^18^F-radiolabeling of clinically significant peptides, such as PSMA-11 and NOTA-Octreotide, were carried out in with high efficiency in these buffers. Thus, the developed technique of Al^18^F-radiolabeling can subsequently be adapted for the synthesis of a wide range of clinically significant peptides.

## Figures and Tables

**Table 1:** Some experimental details and elemental analysis for BEA salts **1–16**.

No.	Formula	Aggregate State	Yield, %	M.P., °C	Elemental Analysis, %					
					Calculated			Found		
					C	H	N	C	H	N
**1**	C_16_H_19_NO_3_	Solid	87	109	70.31	7.01	5.12	70.85	7.33	4.81
**2**	C_18_H_21_NO_3_	Solid	88	113	72.22	7.07	4.68	72.73	7.26	4.43
**3**	C_16_H_19_NO_3_	Liquid	74	-	-					
**4**	C_20_H_21_NO_4_	Liquid	85	-	-					
**5**	C_18_H_23_NO_5_	Liquid	69	-	-					
**6**	C_18_H_22_ClNO_5_	Liquid	67	-	-					
**7**	C_17_H_19_NO_5_	Solid	86	93	64.34	6.03	4.41	64.95	6.33	4.27
**8**	C_26_H_32_N_2_O_6_	Solid	96	91	66.65	6.88	5.98	67.05	6.95	5.74
**9**	C_19_H_19_NO_9_	Solid	99	171	56.30	4.72	3.46	56.84	4.96	3.31
**10**	C_28_H_32_N_2_O_10_	Solid	99	154	60.42	5.80	5.03	60.89	5.99	4.83
**11**	C_13_H_19_NO_5_	Solid	83	98	57.98	7.11	5.20	58.42	6.56	4.72
**12**	C_22_H_32_N_2_O_6_	Solid	71	96	62.84	7.67	6.66	63.27	7.92	6.51
**13**	C_12_H_17_NO_5_	Liquid	63	-	-					
**14**	C_21_H_30_N_2_O_6_	Liquid	60	-	-					
**15**	C_11_H_15_NO_5_	Solid	75	183	54.77	6.27	5.81	55.38	5.96	5.76
**16**	C_20_H_28_N_2_O_6_	Solid	82	163	61.21	7.19	7.14	61.79	6.79	6.81

**Table 2:** Crystal data and structure refinement for BEA salts **11**, **15** and **16**.

	BEA Salts		
	11	15	16
Chemical formula	C_13_H_19_NO_5_	C_11_H_15_NO_5_	C_10_H_14_NO_3_
M_r_	269.29	241.24	196.22
Crystal system, space group	Monoclinic, *P*2_1_/*c*	Monoclinic, *P*2_1_/*n*	Triclinic, *P*-1
a, b, c (Å)	12.0752(1); 15.8387(2); 14.0684(2)	11.0513(2); 13.5777(2); 15.7497(2)	6.1853(3); 8.4052(3); 9.9266(3)
α, β, γ (°)	90; 90.697(1); 90	90; 101.412(2); 90	108.291(3); 100.797(4); 90.629(4)
V (Å^3^)	2690.45(6)	2316.53(6)	479.97(3)
Z	8	8	2
Radiation type	CuKα (λ = 1.54184 Å)	CuKα (λ = 1.54184 Å)	CuKα (λ = 1.54184 Å)
µ (mm^−1^)	0.855	0.930	0.831
Crystal size (mm)	0.22 × 0.17 × 0.10	0.18 × 0.14 × 0.10	0.22 × 0.14 × 0.09
Diffractometer	Rigaku «XtaLAB Synergy»	Rigaku «XtaLAB Synergy»	Rigaku «XtaLAB Synergy»
T, K	100	100	100
Absorption correction	Multi-scan	Multi-scan	Multi-scan
T_min_, T_max_	0.968; 1.000	0.850; 1.000	0.831; 1.000
No. of measured, independent and observed [I > 2σ(I)] reflections	14,439; 5000; 4445	12,011; 4300; 3717	3485; 1775; 1608
R_int_	0.0301	0.0302	0.0289
θ_min_, θ_max_	3.661; 69.096	4.336; 69.166	4.79; 69.034
R[F^2^ > 2σ(F^2^)], wR(F^2^), S	0.0349; 0.0922; 1.044	0.0375; 0.1026; 1.035	0.0408; 0.1127; 1.049
No. of reflections	5000	4300	1775
No. of parameters	359	323	131
No. of restraints	0	4	0
H-atom treatment	mixed	mixed	mixed
Δρ_max_, Δρ_min_ (eÅ^−3^)	0.21; −0.22	0.36; −0.24	0.25; −0.24

**Table 3:** Some observed FTIR bands (cm^−1^) and their assignment of BEA salts **1**–**16** (w = weak; m = medium, s = strong; br = broadened).

No.	ν(OH)	ν(CH), ν(CH_2_), ν(CH_3_)	ν(N^+^H_2_)	ν_as_(COO^−^), ν_s_(COO^−^)	ν(Ar)	ν(COOH)
**1**	3190	3010, 2925, 2875, 2780, 2730	2630 (br.), 2500, 2430	1530, 1380	1640, 1590 (br.), 1460	-
**2**	3180	3030, 2925, 2875, 2780, 2730	2590, 2520, 2430	1540, 1380	1650, 1490, 1450	-
**3**	3115 (br.)	3010, 2940, 2810, 2720	2600, 2450	1550, 1340	1600, 1480, 1460	-
**4**	3270 (br.w)	3060, 2945, 2874, 2825	2600 (br.w), 2420 (br.w)	1580, 1404	1625, 1505, 1466, 1435	-
**5**	3200 (br.)	3020, 2920, 2790 (br.)	2660 (br.), 2420 (br.)	1580, 1400	1490, 1460	-
**6**	3200 (br.)	3030, 2950, 2920, 2790 (br.)	2400 (br.)	1580, 1400	1490, 1460	-
**7**	3440	2990, 2850, 2755	2650, 2600, 2520	1624, 1380	1685, 1590, 1455	1700
**8**	3445	3000, 2960, 2850, 2755	2655, 2610, 2523	1620, 1400	1680, 1590, 1455	-
**9**	3120	2975, 2865, 2790	2600, 2470	1540, 1393	1650, 1455	1700
**10**	3440, 3385	3030, 3010, 2955, 2880, 2830	2490 (br.w)	1560, 1323	1455	1700 w
**11**	3250 (br.)	3030, 2950	2650 (br.), 2540 (br.)	1580, 1400	1500, 1460, 1430	1700
**12**	3220 (br.)	3060, 3030, 2940, 2820	2530 (br. w), 2370, 2350	1555, 1390	1500, 1455	-
**13**	3300 (br.)	3040, 2950, 2790 (br.)	2670 (br.) 2540 (br.)	1570, 1350	1450, 1410	1710
**14**	3180 (br.)	3030, 2950, 2810	2670 (br.), 2540 (br.), 2400 (br.)	1550, 1330	1500, 1450	-
**15**	3385 (br.)	3036, 2970, 2900, 2860, 2830	2680 (br.w), 2430 (br.w)	1540, 1420	1651, 1462	1700
**16**	3210	2965, 2940, 2830, 2760	2665, 2595, 2530	1580, 1380	1455	-

**Table 4:** Hydrogen bonding geometry for BEA salts **11**, **15** and **16**.

D-H···A	D-H (Å)	H···A (Å)	D···A (Å)	∠ D-H···A, °
**11**				
N_24_-H···O_9_	0.91	1.90	2.782 (1)	162.3
N_24_-H···O_8_	0.91	1.82	2.717 (1)	168.5
N_24_-H···O_8_	0.85	1.90	2.729 (1)	168 (2)
N_24_-H···O_8_	0.91	1.99	2.868 (1)	161.8
N_24_-H···O_8_	0.91	1.92	2.820 (1)	169.4
N_24_-H···O_8_	0.88	1.86	2.735 (1)	172 (2)
N_24_-H···O_8_	0.92	1.67	2.595 (1)	178 (2)
N_24_-H···O_8_	0.95	1.65	2.589 (1)	171 (2)
**15**				
N_20_-H···O_6_	0.91	1.96	2.854 (1)	166.9
N_20_-H···O_4_	0.91	2.01	2.884 (1)	160.6
O_23_-H···O_4_	0.82	1.97	2.789 (1)	179 (2)
O_1_-H···O_10_	0.95	1.50	2.450 (1)	175 (2)
N_31_-H···O_12_	0.91	1.99	2.888 (1)	167.0
N_31_-H···O_11_	0.91	1.96	2.826 (1)	158.9
O_34_-H···O_23_	0.82	2.03	2.845 (1)	169 (2)
O_7_-H···O_5_	0.93	1.55	2.481 (1)	175 (2)
		**16**		
N_11_-H···O_3_	0.91	1.91	2.762 (1)	155.9
N_11_-H···O_1_	0.91	1.93	2.781 (1)	155.7
O_14_-H···O_1_	0.89	1.85	2.739 (2)	177 (3)

**Table 5:** The ADME properties of BEA salts **1**–**16** and drugs trekrezan and chlorcrezacin (M_w_ = molecular weight (g/mol); HBD = number of H-bond donors; HBA = number of H-bond acceptors; RB = number of rotatable bonds; log *P* = an octanol/water partition coefficient; Vio = number of Lipinski’s rule violations; GIA = gastrointestinal absorption; BAS = bioavailability score; SA = synthetic accessibility).

Salt	M_w_	HBD	HBA	RB	log *P*	Lipinski’s Rule (Vio)	GIA	BAS	SA
Trekrezan	315.36	4	6	9	−1.53	Yes (0)	Low	0.55	2.31
Chlorcrezacin	349.81	4	6	9	−0.99	Yes (0)	Low	0.55	2.48
**1**	273.33	2	3	5	0.54	Yes (0)	High	0.55	1.43
**2**	299.36	2	3	6	0.75	Yes (0)	High	0.55	2.26
**3**	289.33	3	4	5	0.48	Yes (0)	High	0.55	1.63
**4**	339.39	3	4	5	1.01	Yes (0)	High	0.55	1.89
**5**	317.38	2	4	7	0.65	Yes (0)	High	0.55	2.25
**6**	351.82	2	4	7	1.20	Yes (0)	High	0.55	2.40
**7**	317.34	3	5	6	0.11	Yes (0)	High	0.56	1.79
**8**	468.54	4	6	10	−0.85	Yes (0)	Low	0.55	2.69
**9**	405.36	5	9	8	−0.86	Yes (0)	Low	0.11	2.32
**10**	556.56	6	10	12	−1.90	No (3)	Low	0.11	3.24
**11**	269.29	3	5	7	−0.72	Yes (0)	High	0.56	1.55
**12**	420.50	4	6	11	−1.70	Yes (0)	Low	0.55	2.44
**13**	255.27	3	5	6	−0.88	Yes (0)	Low	0.56	1.34
**14**	406.47	4	6	10	−1.81	Yes (0)	Low	0.55	2.23
**15**	241.24	3	5	5	−1.11	Yes (0)	Low	0.56	1.23
**16**	392.45	4	6	9	−1.87	Yes (0)	Low	0.55	2.10

## Data Availability

Not applicable.

## Supplementary Materials

The following are available online at https://www.mdpi.com/article/10.3390/pharmaceutics15020694/s1. Figures S1–S16: ATR-FTIR spectra of BEA salts **1**–**16**; Figures S17a–S31a: ^1^H NMR spectra of BEA salts **1**–**16**; Figures S17b–S31b: ^13^C NMR spectra of BEA salts **1**–**16**; Figures S32–S34: pH profile of buffer **3**, **4** and **12**; Table S1: pH value of reaction mixtures for ^68^Ga- and Al^18^F-radiolabeling reactions.
