# Supplementary material for: N-Benzylethanolammonium Ionic Liquids and Molten Salts in the Synthesis of 68Ga- and Al18F-Labeled Radiopharmaceuticals
Source: Pharmaceutics. 2023 Feb 18;15(2):694. doi: 10.3390/pharmaceutics15020694 (PMC9962170; doi:10.3390/pharmaceutics15020694)
Supplement: Supplementary file 1 [file pharmaceutics-15-00694-s001.zip › pharmaceutics-2213570-supplementary.pdf]

Supplementary Materials

N-benzylethanolammonium ionic liquids and molten salts in the synthesis of ^{68}Ga- and Al^{18}F-labelled radiopharmaceuticals

Yulia A. Kondratenko, Julia S. Shilova, Vladislav A. Gavrilov, Andrey A. Zolotarev,
Michail A. Nadporojskii, Tatyana A. Kochina and Dmitrii O. Antuganov

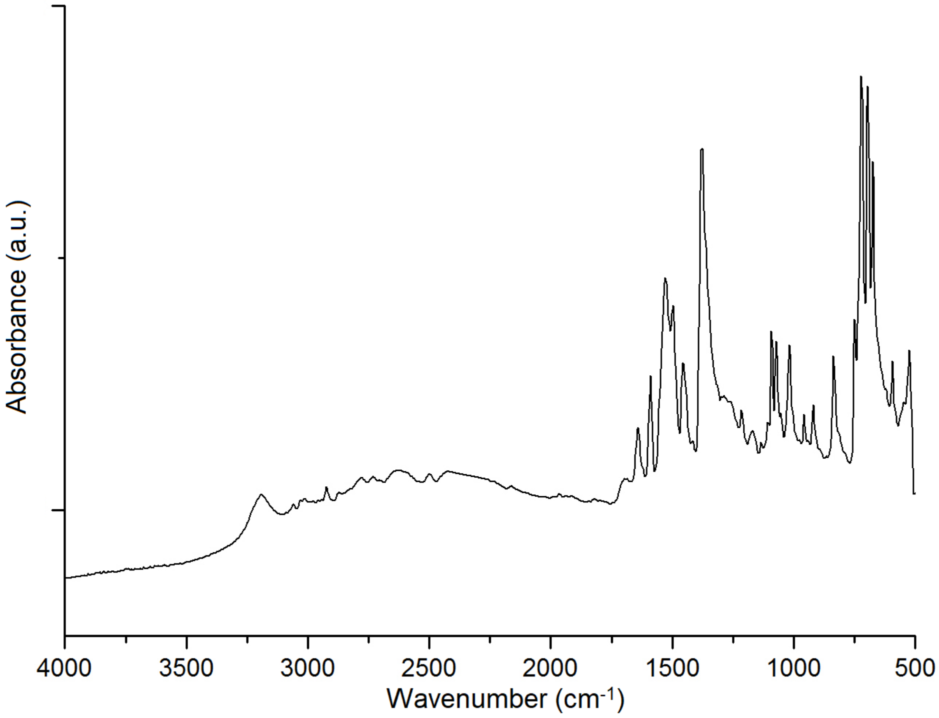

Figure S1. ATR-FTIR spectrum of BEA salt **1**

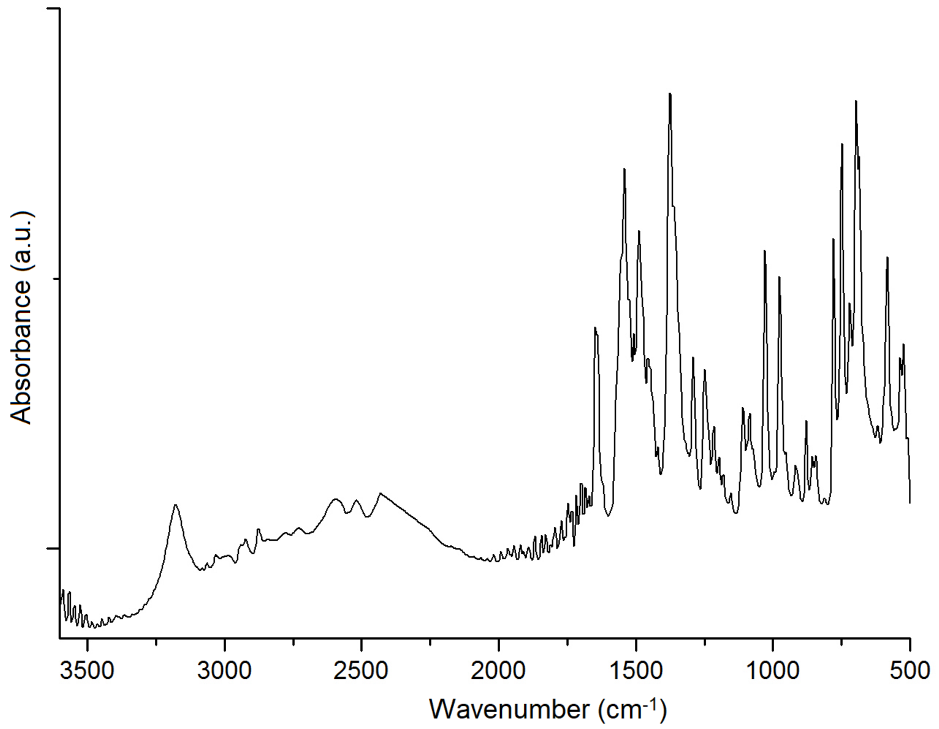

Figure S2. ATR-FTIR spectrum of BEA salt **2**

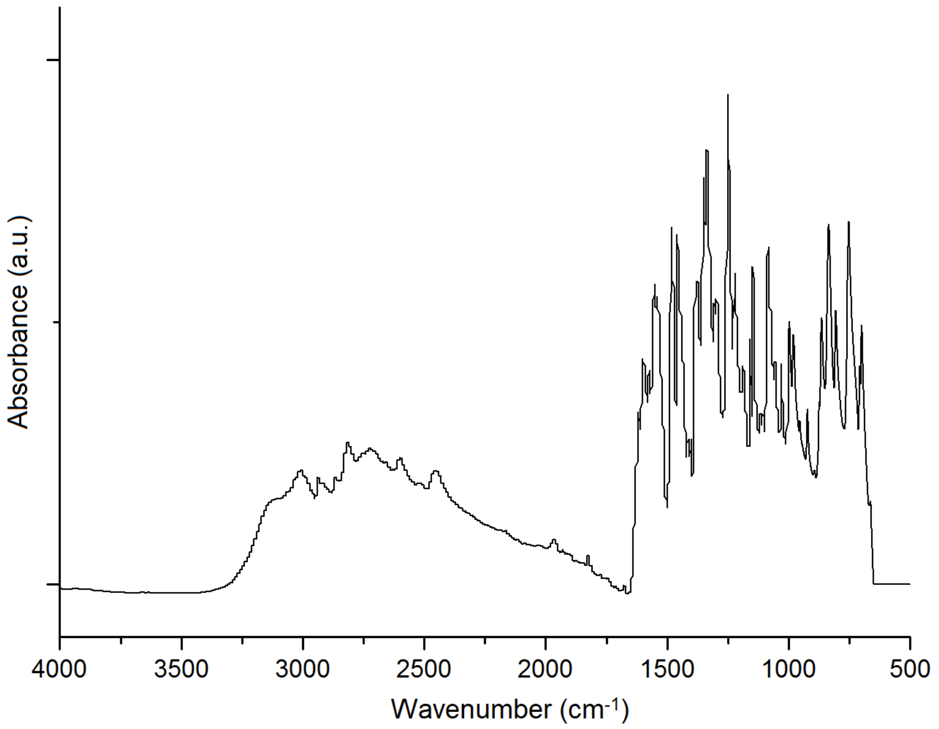

Figure S3. ATR-FTIR spectrum of BEA salt **3**

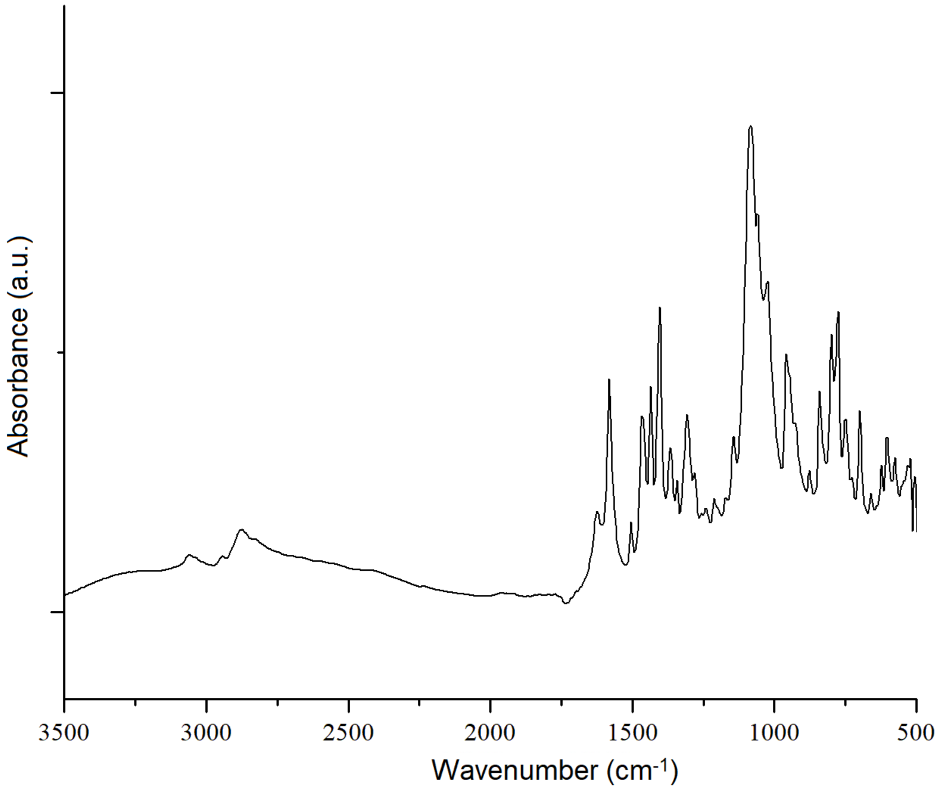

Figure S4. ATR-FTIR spectrum of BEA salt **4**

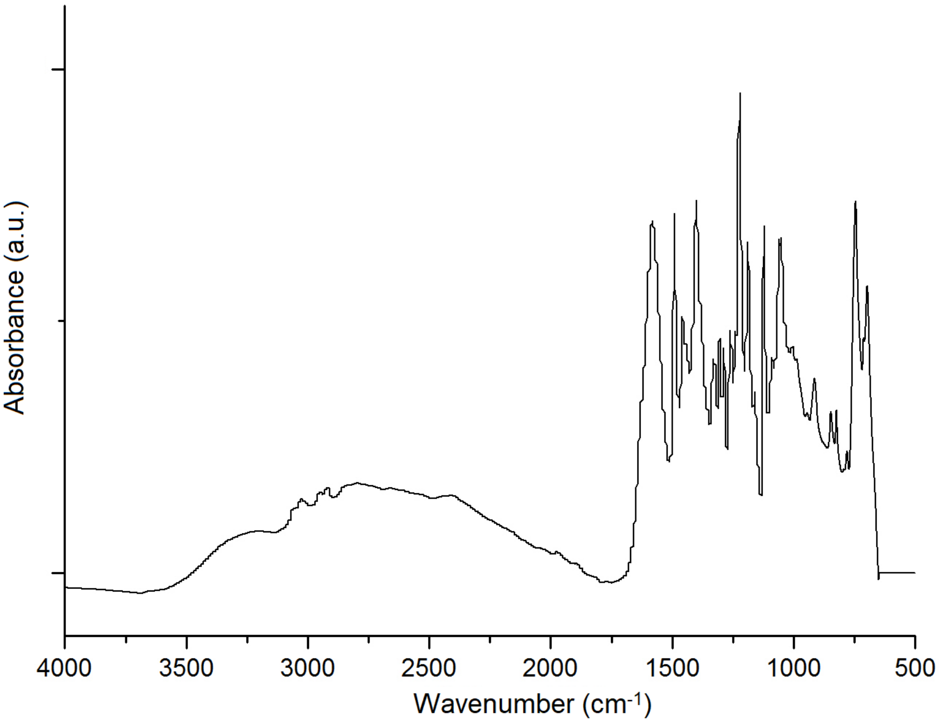

Figure S5. ATR-FTIR spectrum of BEA salt **5**

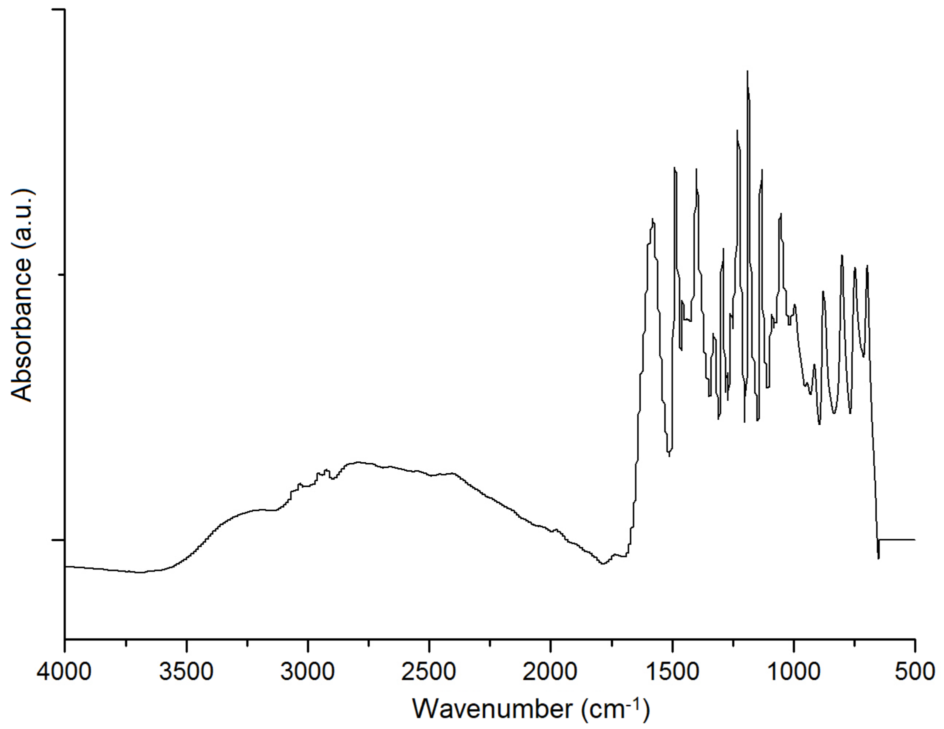

Figure S6. ATR-FTIR spectrum of BEA salt **6**

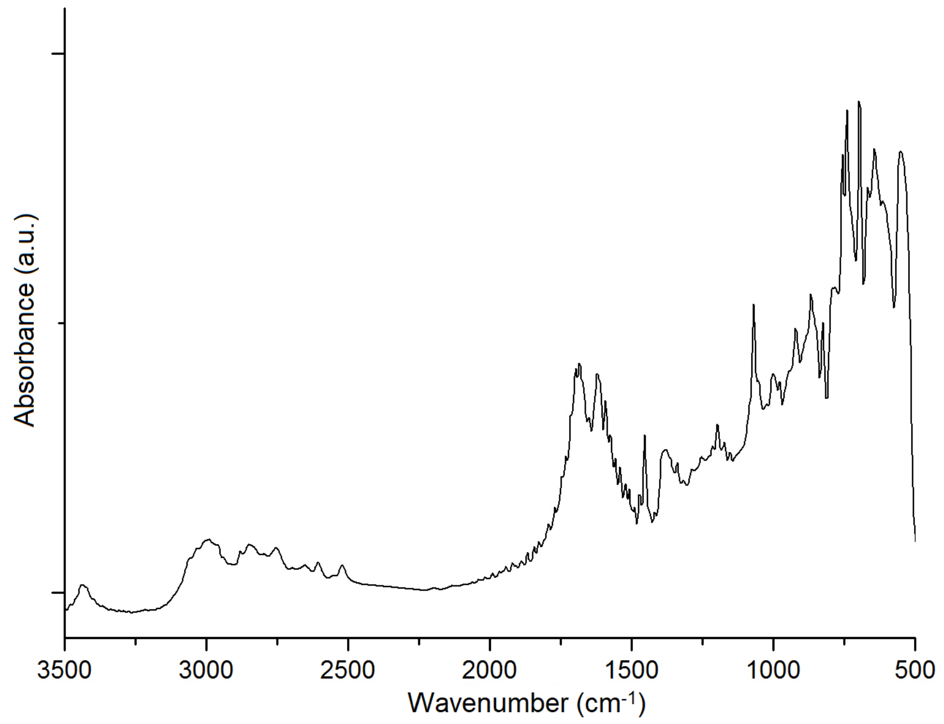

Figure S7. ATR-FTIR spectrum of BEA salt **7**

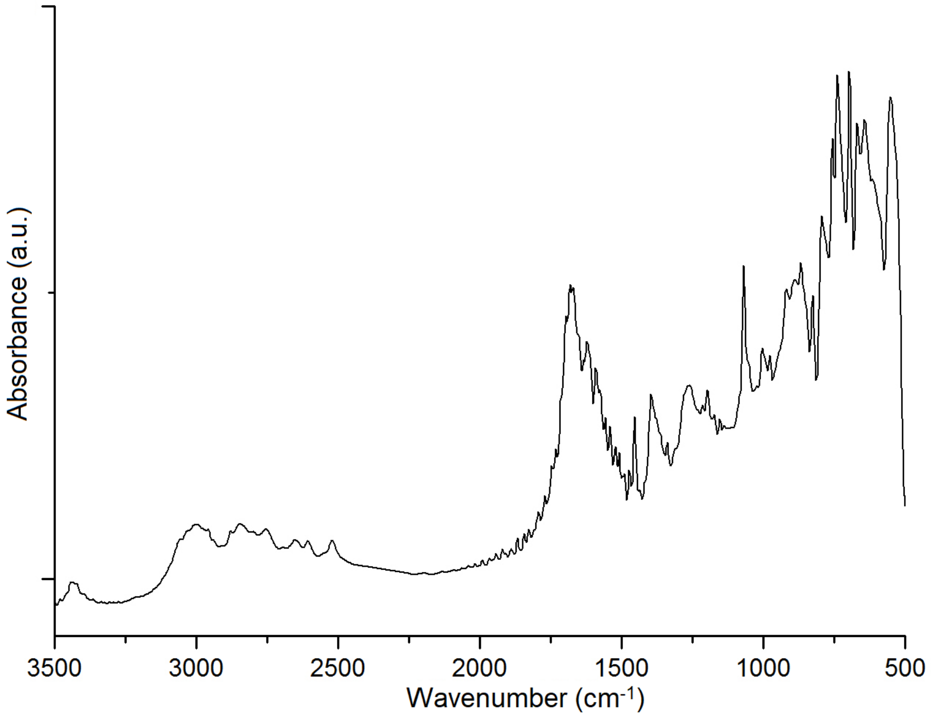

Figure S8. ATR-FTIR spectrum of BEA salt **8**

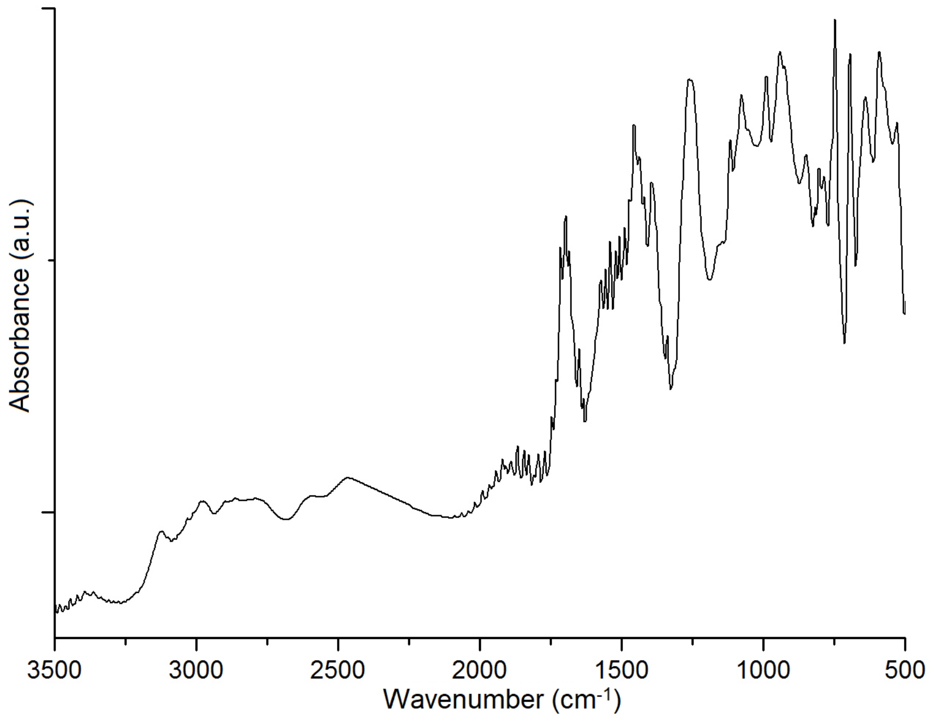

Figure S9. ATR-FTIR spectrum of BEA salt **9**

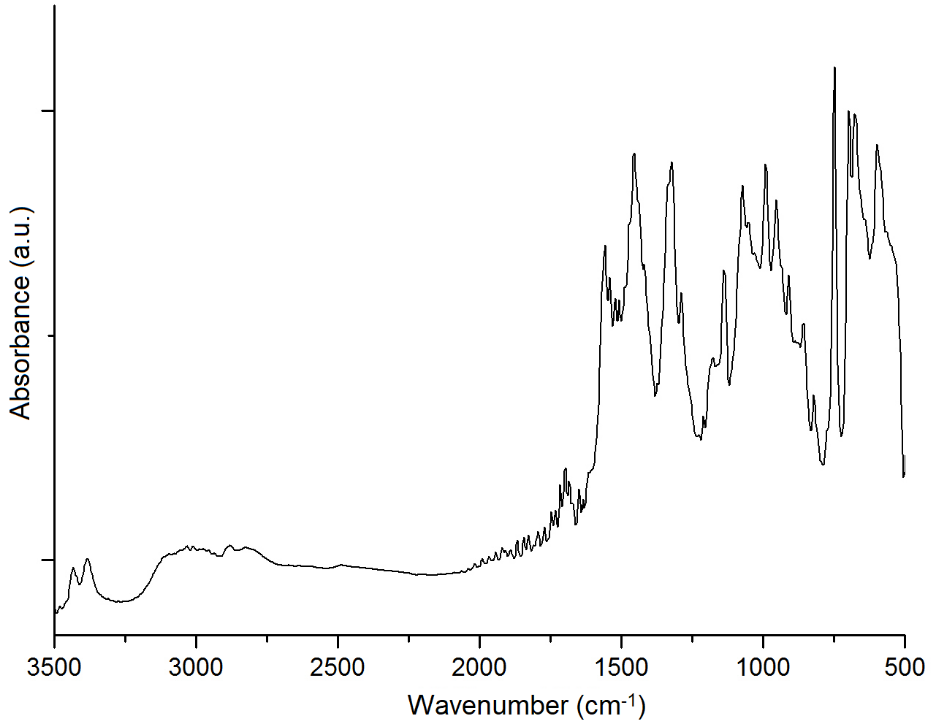

Figure S10. ATR-FTIR spectrum of BEA salt **10**

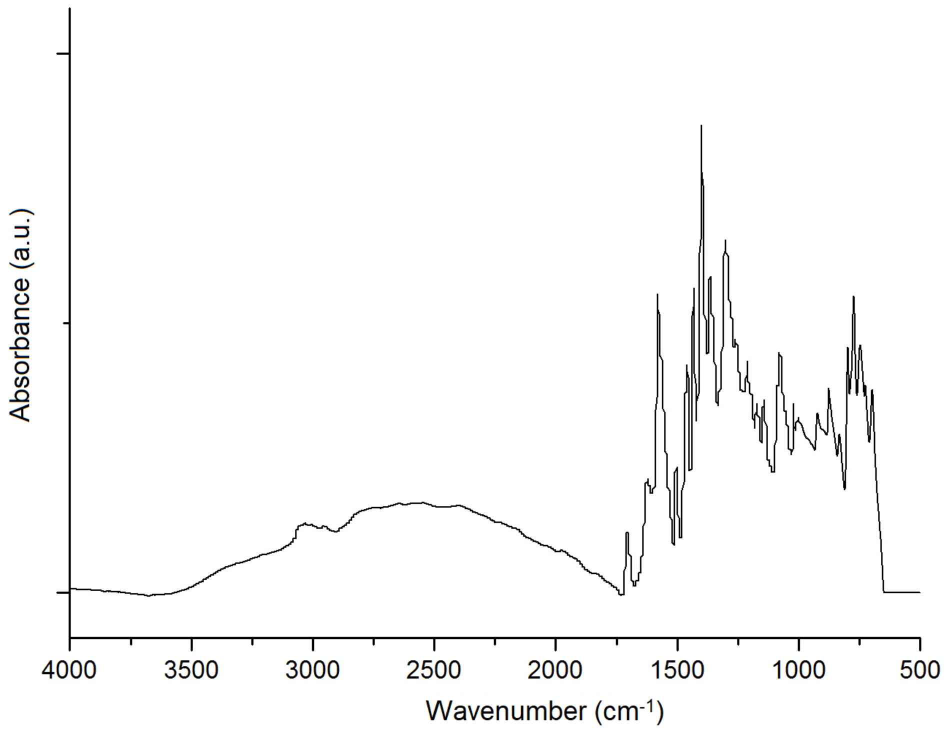

Figure S11. ATR-FTIR spectrum of BEA salt **11**

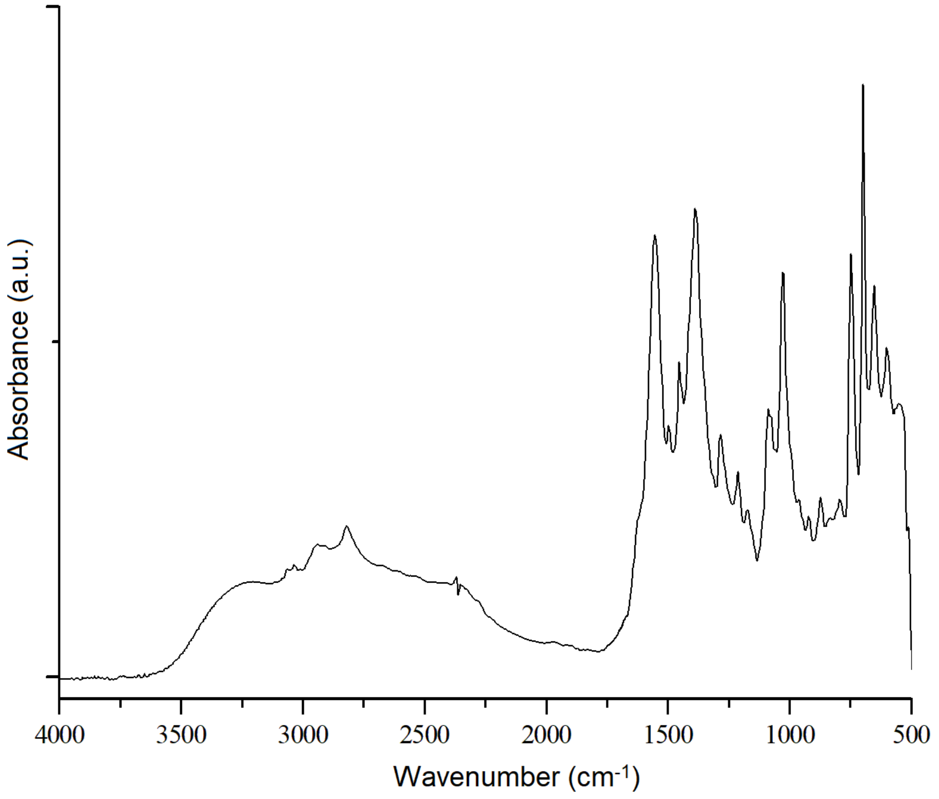

Figure S12. ATR-FTIR spectrum of BEA salt **12**

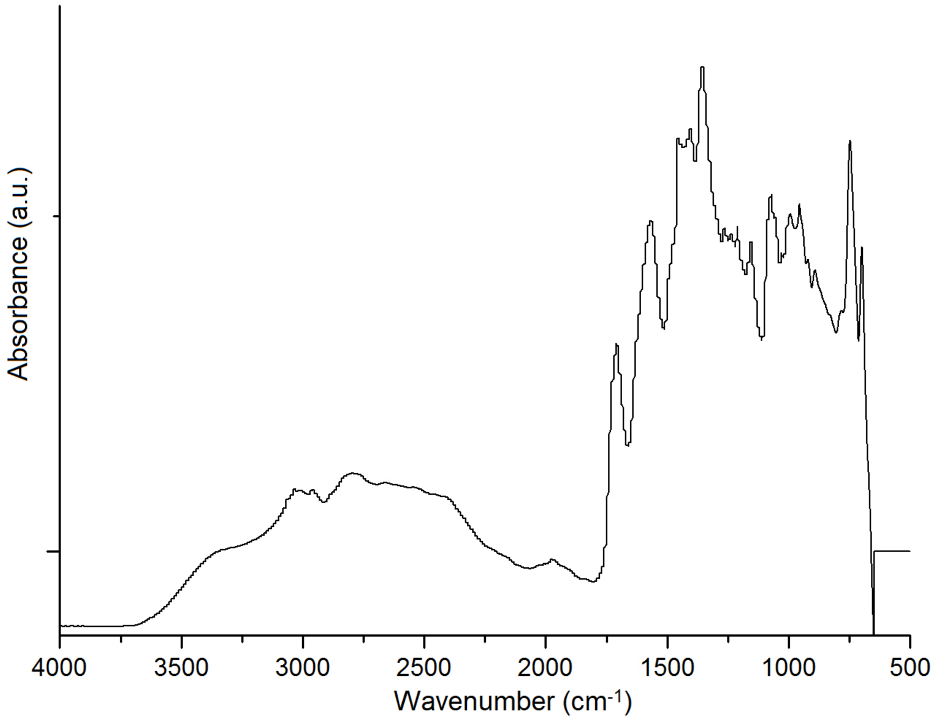

Figure S13. ATR-FTIR spectrum of BEA salt **13**

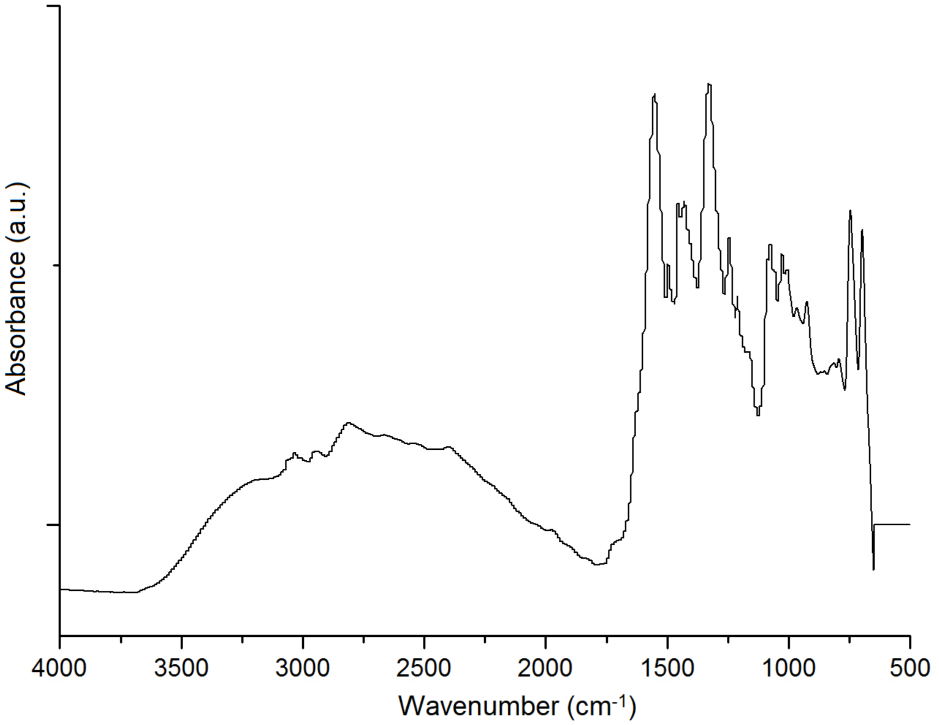

Figure S14. ATR-FTIR spectrum of BEA salt **14**

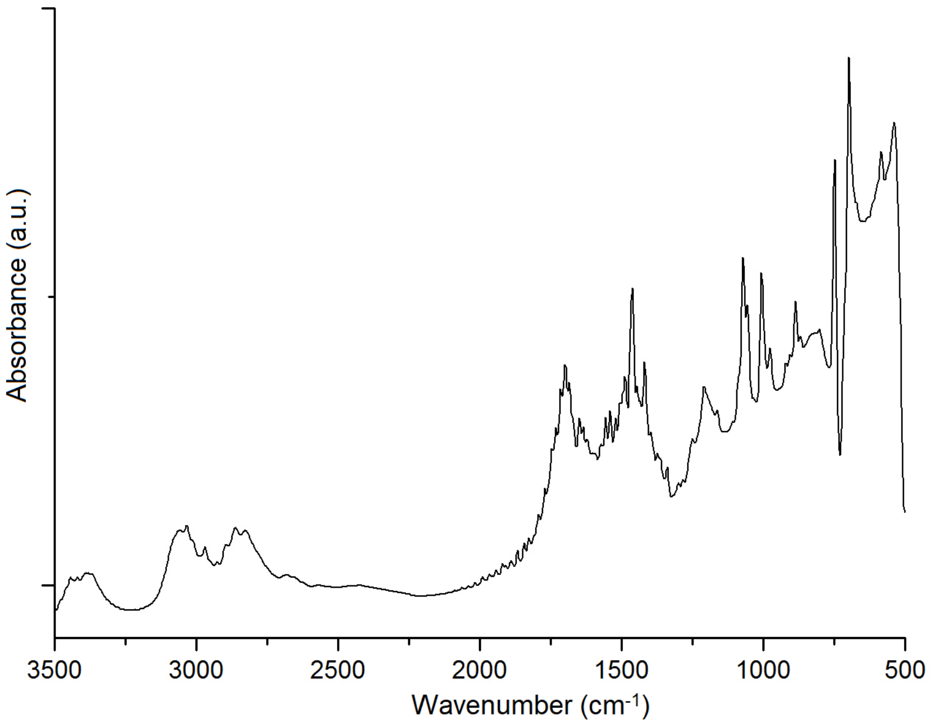

Figure S15. ATR-FTIR spectrum of BEA salt **15**

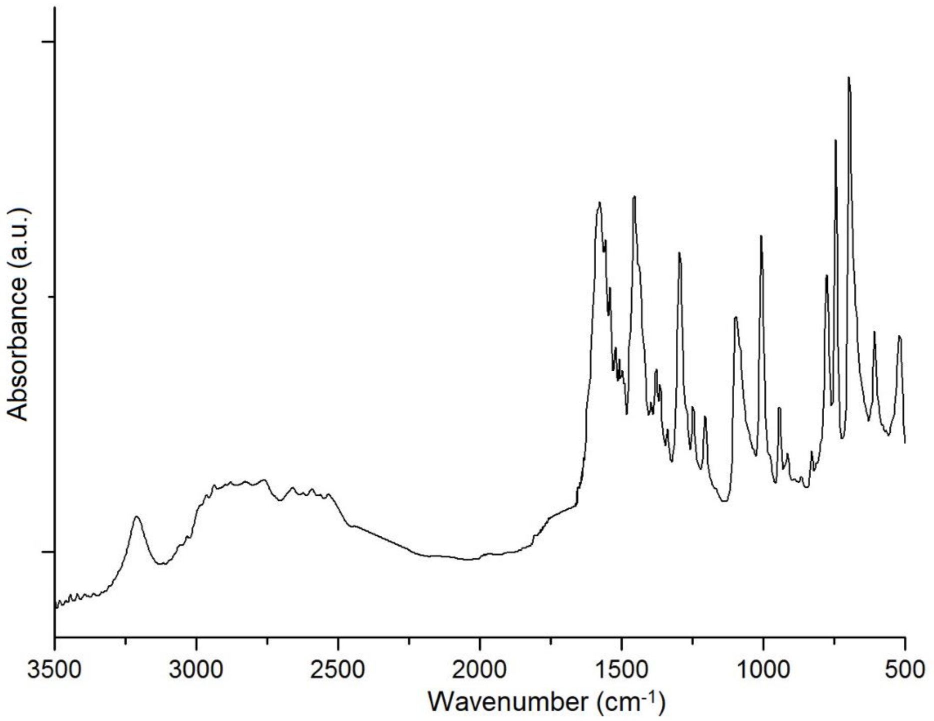

Figure S16. ATR-FTIR spectrum of BEA salt **16**

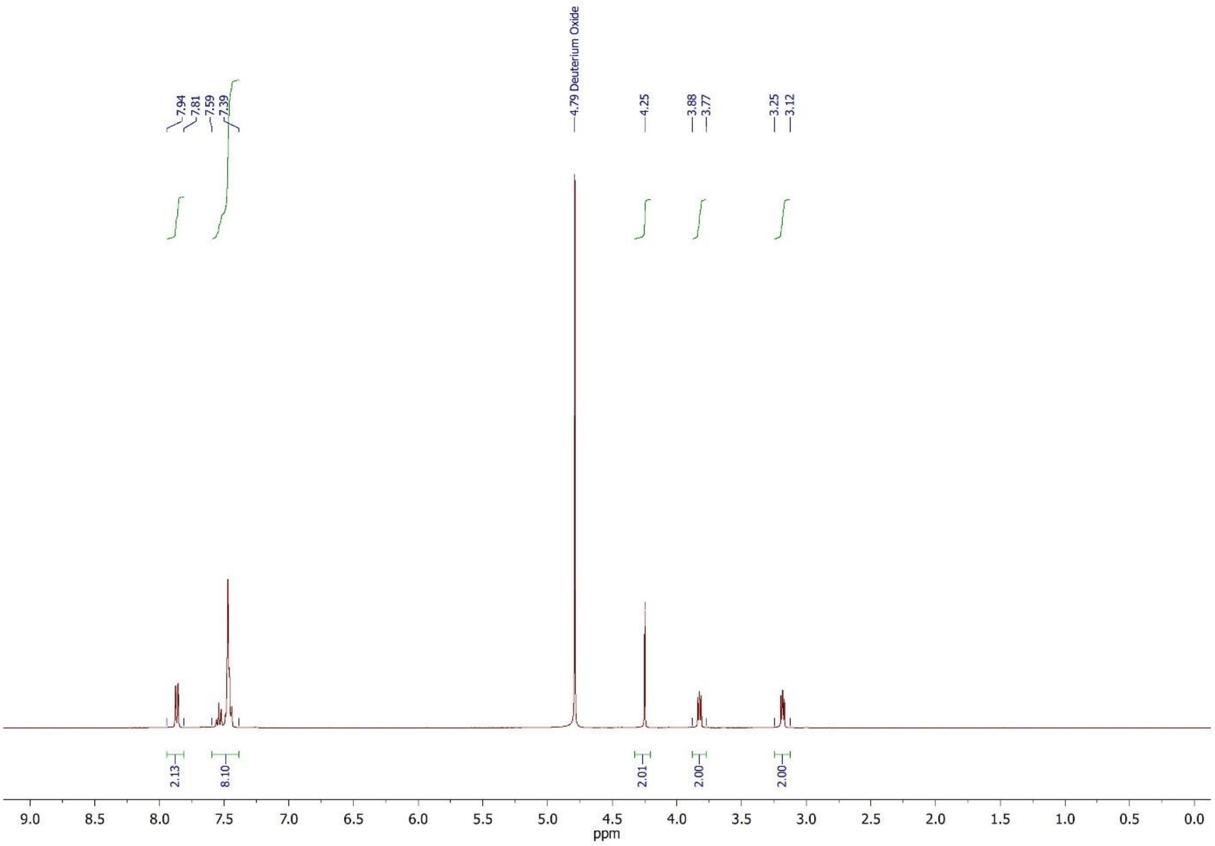

Figure S17 (a).^{1}H NMR Spectrum of **1** (D_2O, δ, ppm, *J*, Hz)

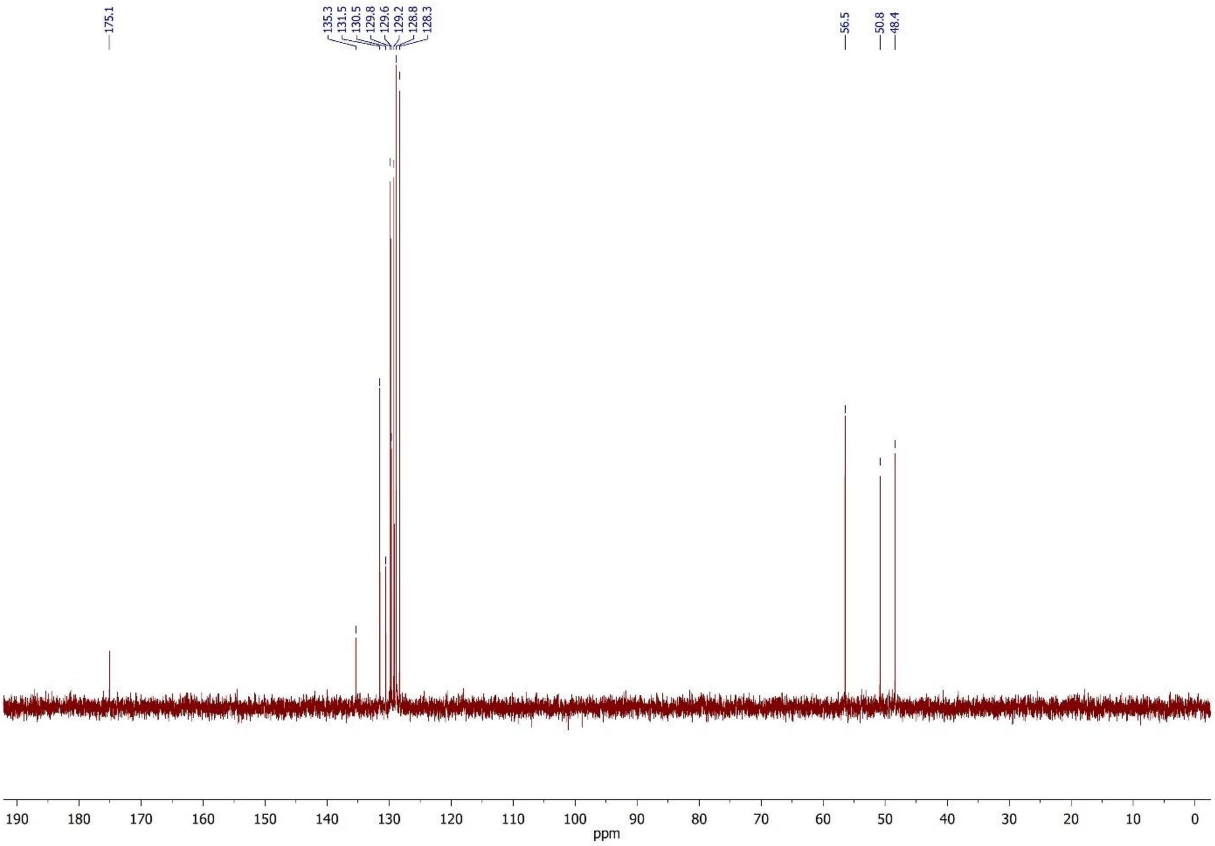

Figure S17 (b). ^{13}C NMR Spectrum of **1** (D_2O, δ, ppm)

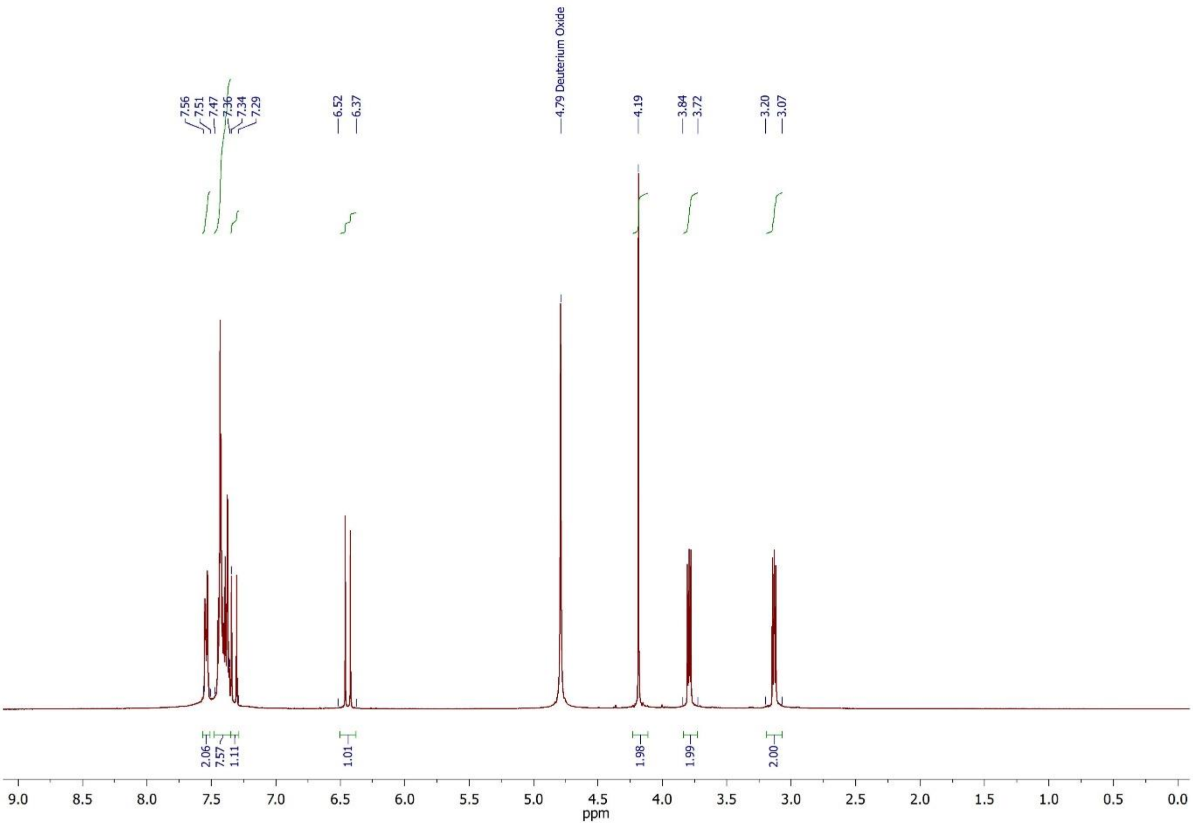

Figure S18 (a).^{1}H NMR Spectrum of **2** (D_2O, δ, ppm, *J*, Hz)

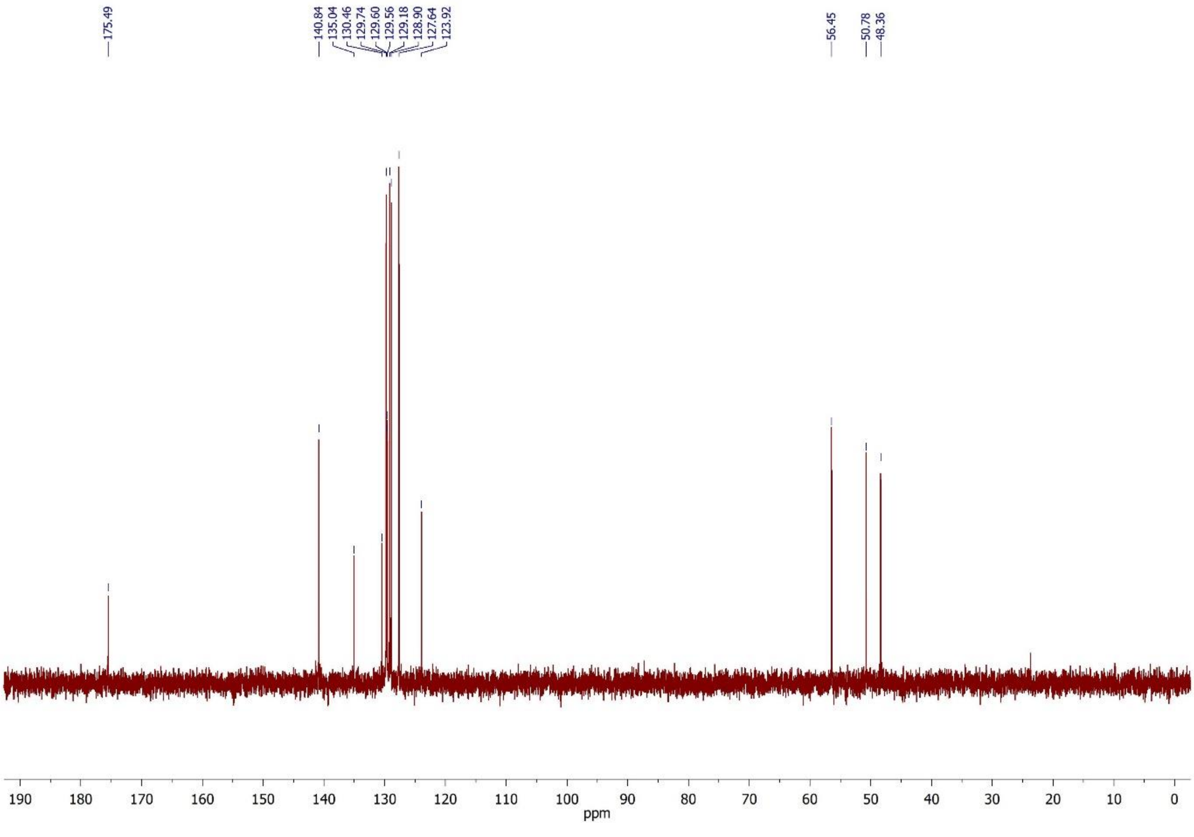

Figure S18 (b). ^{13}C NMR Spectrum of **2** (D_2O, δ, ppm)

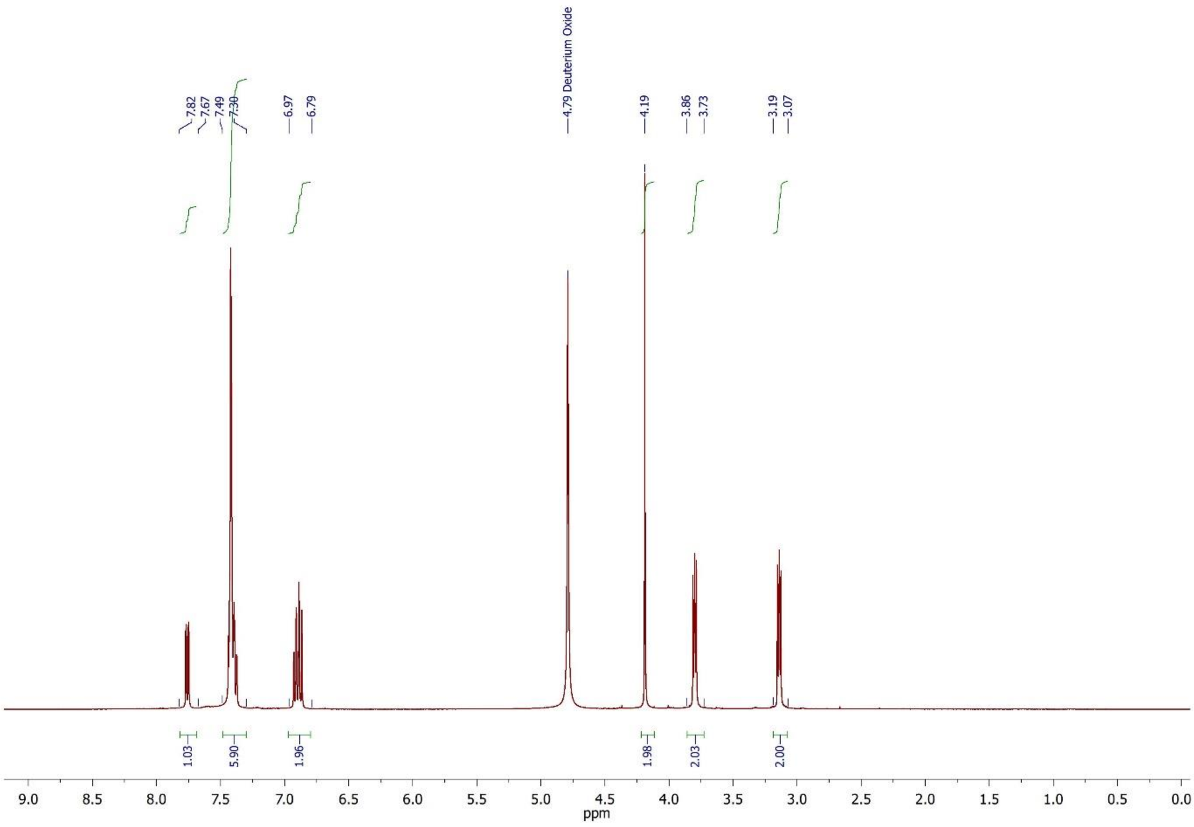

Figure S19 (a).^{1}H NMR Spectrum of **3** (D_2O, δ, ppm, *J*, Hz)

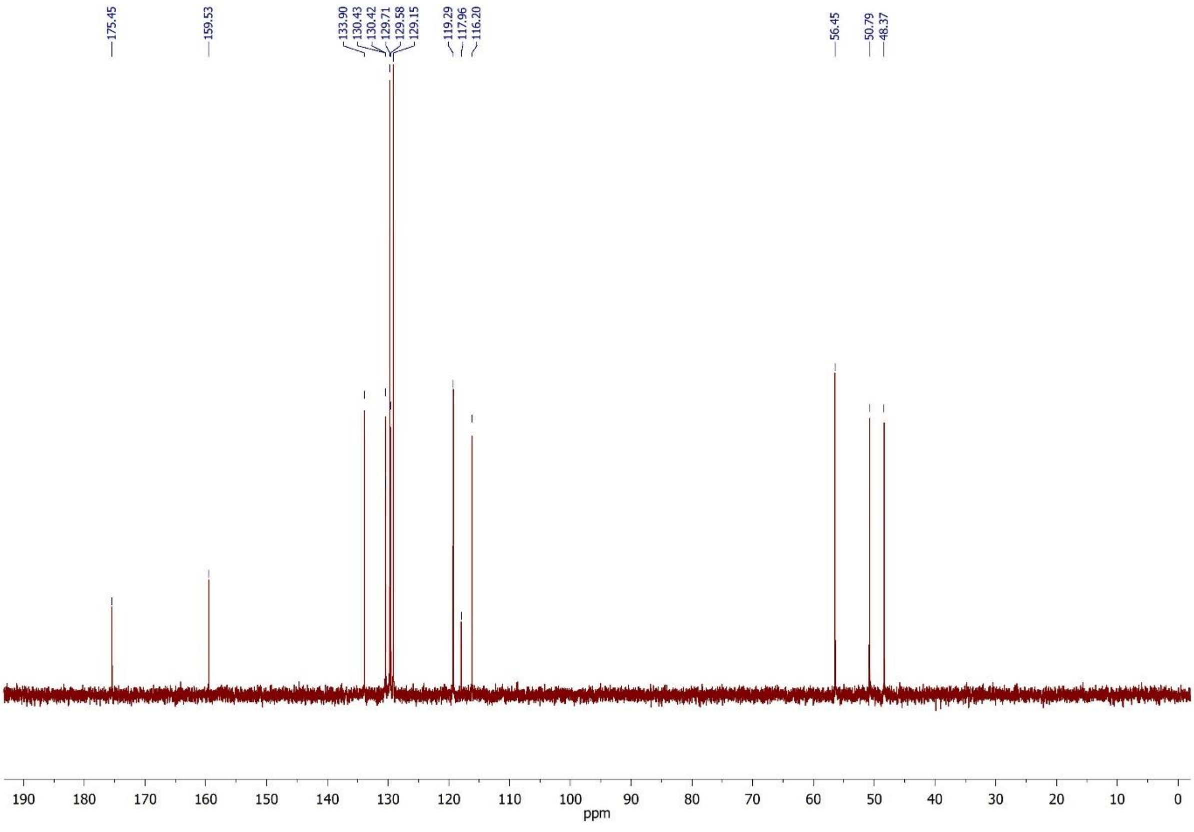

Figure S19 (b). ^{13}C NMR Spectrum of **3** (D_2O, δ, ppm)

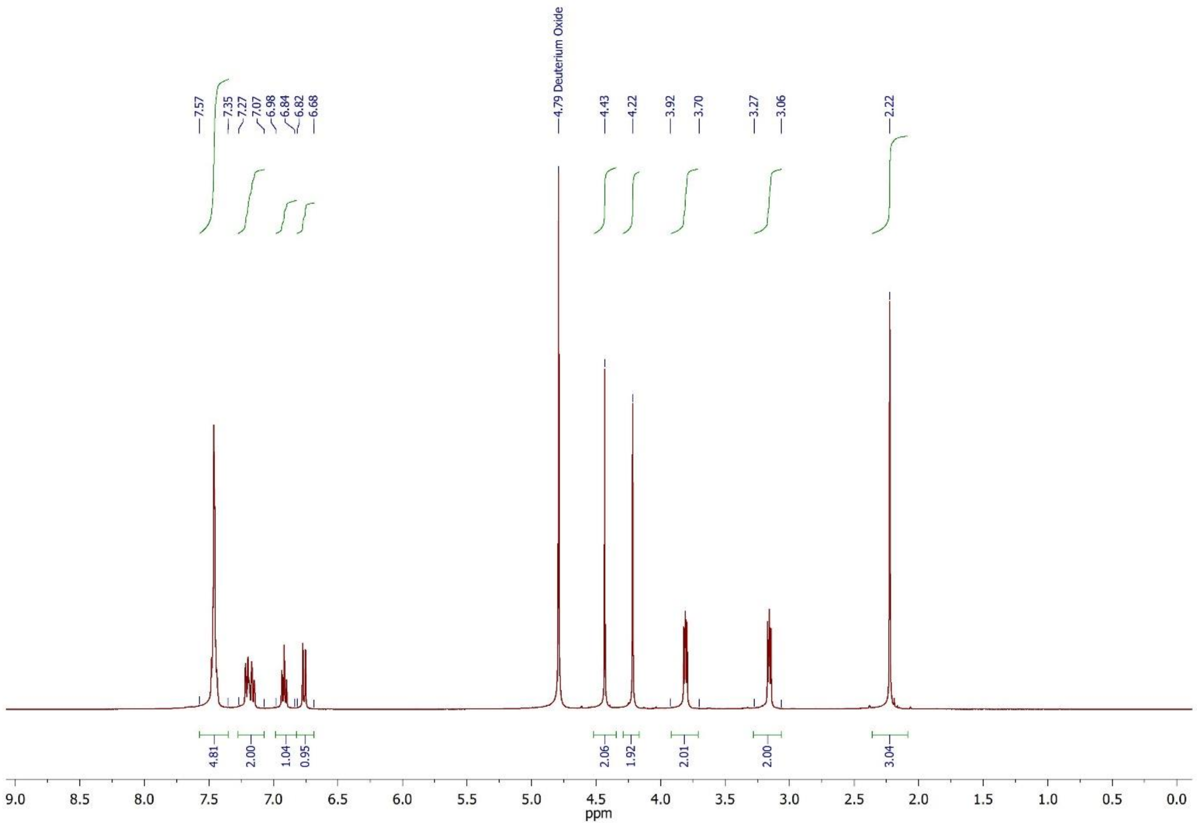

Figure S20 (a).^{1}H NMR Spectrum of **5** (D_2O, δ, ppm, *J*, Hz)

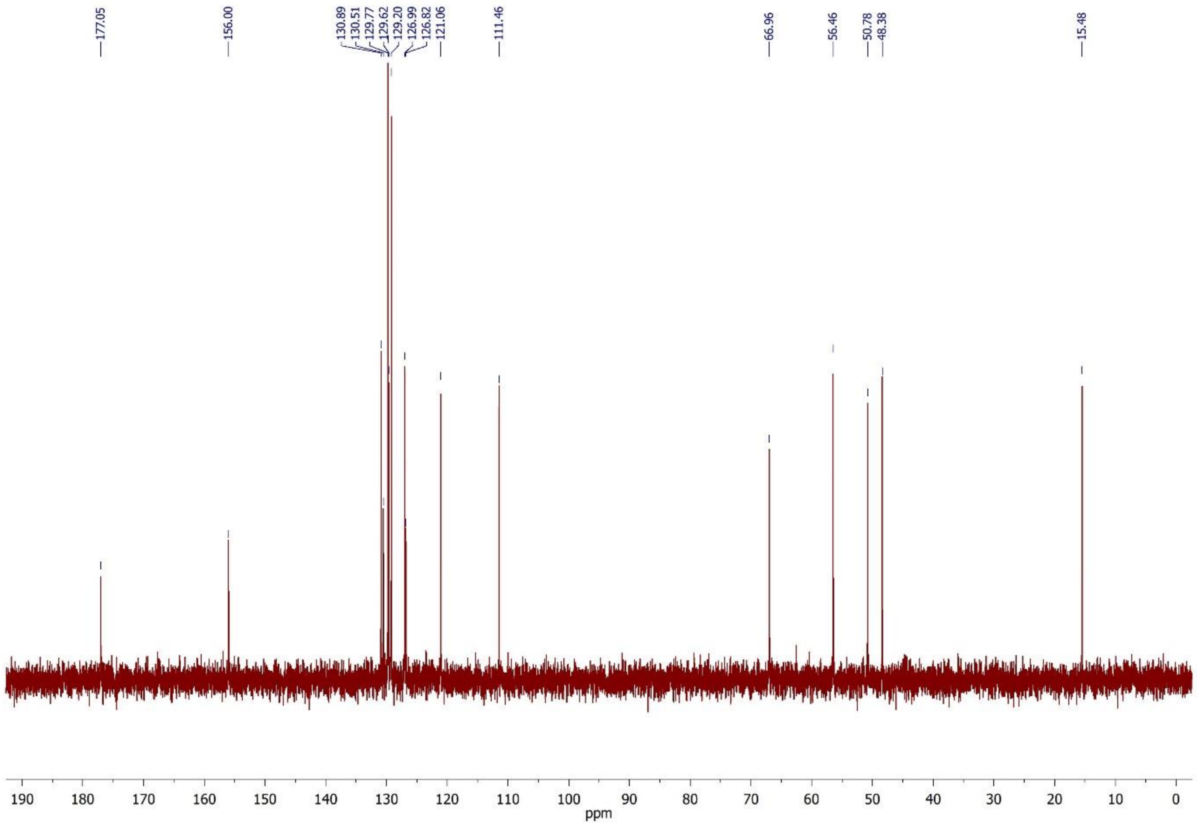

Figure S20 (b). ^{13}C NMR Spectrum of **5** (D_2O, δ, ppm)

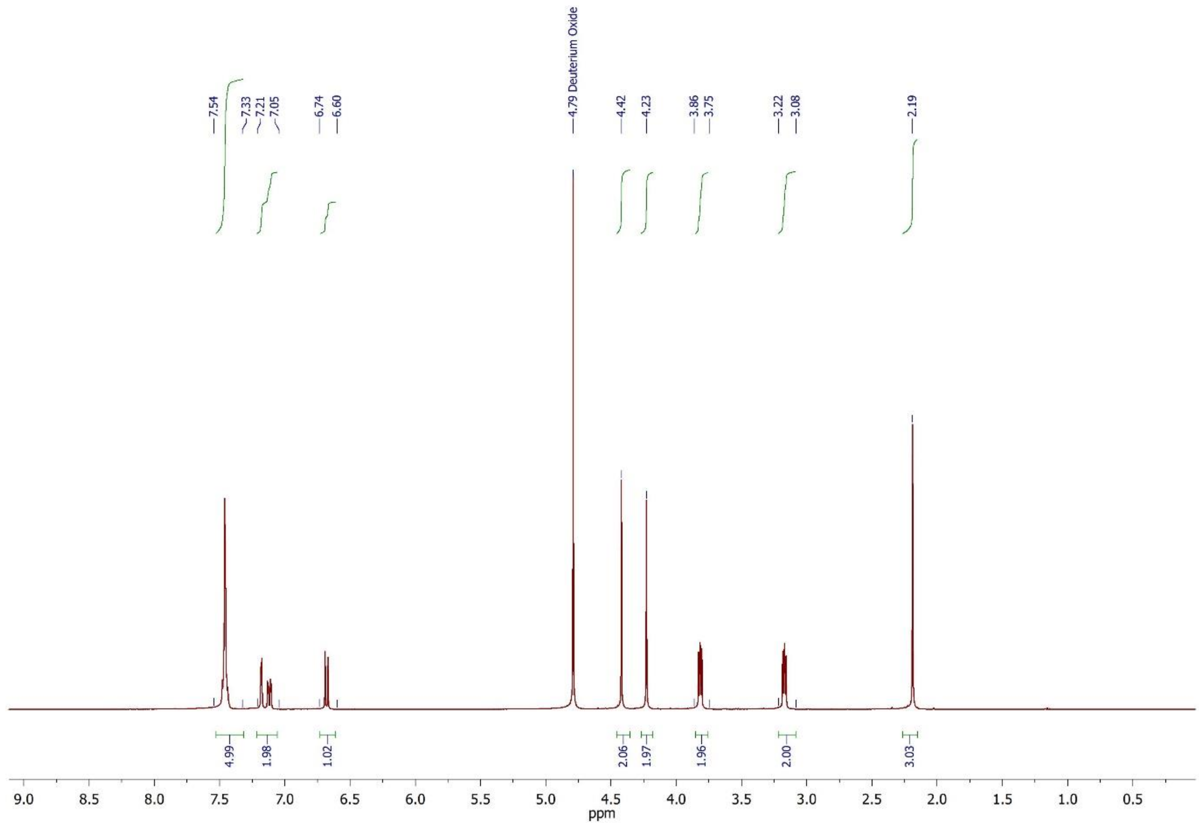

Figure S21 (a).^{1}H NMR Spectrum of **6** (D_2O, δ, ppm, *J*, Hz)

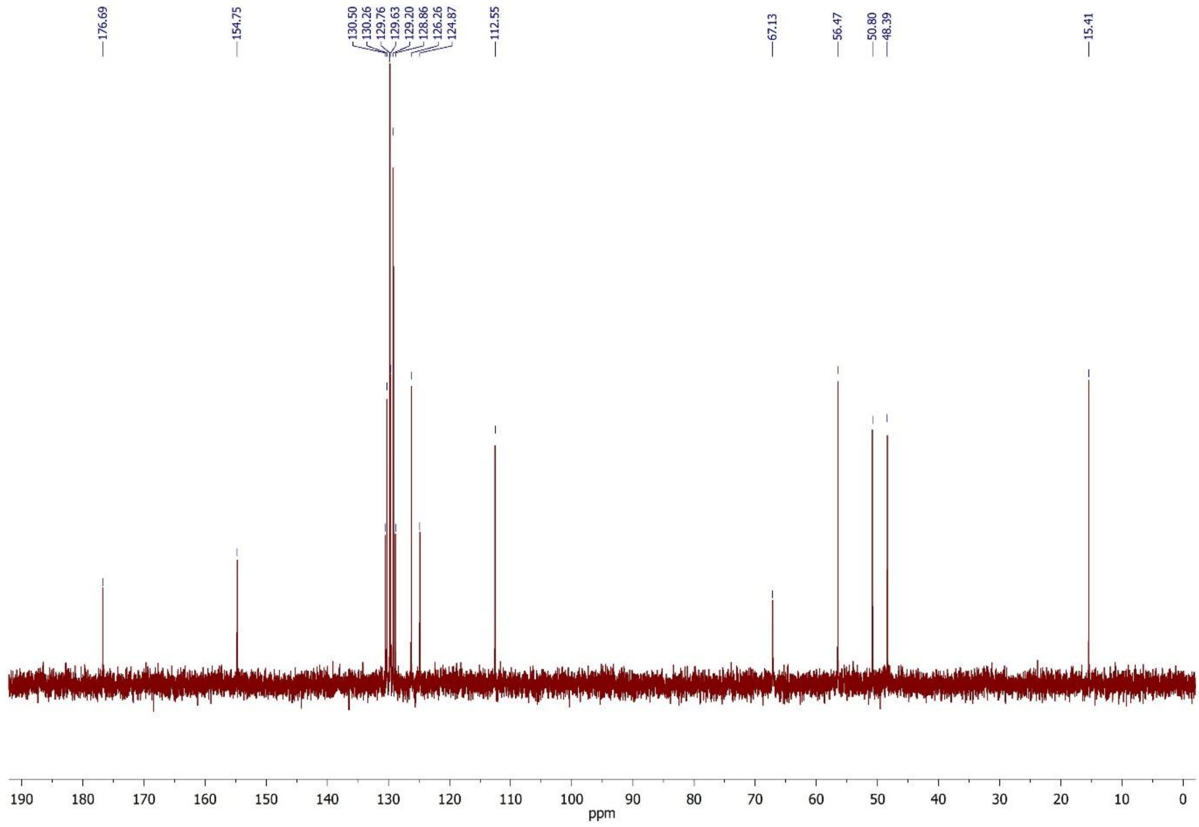

Figure S21 (b). ^{13}C NMR Spectrum of **6** (D_2O, δ, ppm)

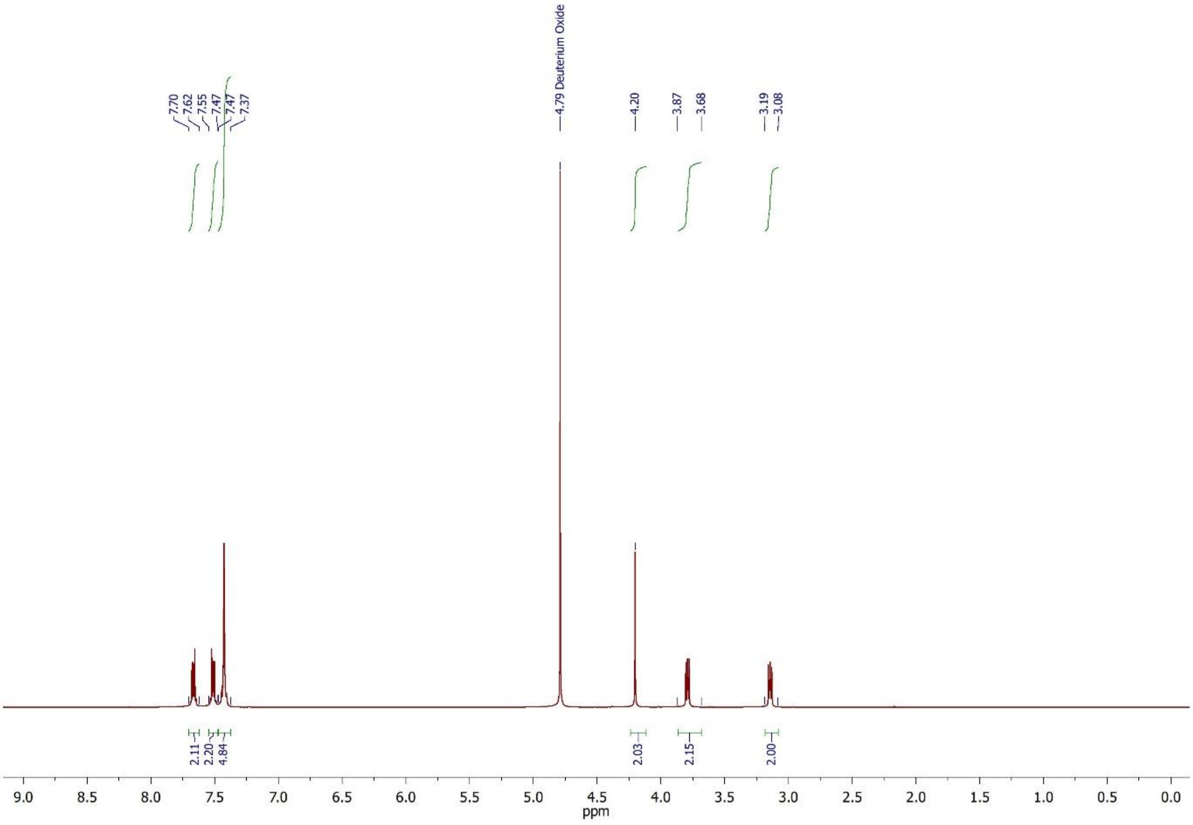

Figure S22 (a).^{1}H NMR Spectrum of **7** (D_2O, δ, ppm, *J*, Hz)

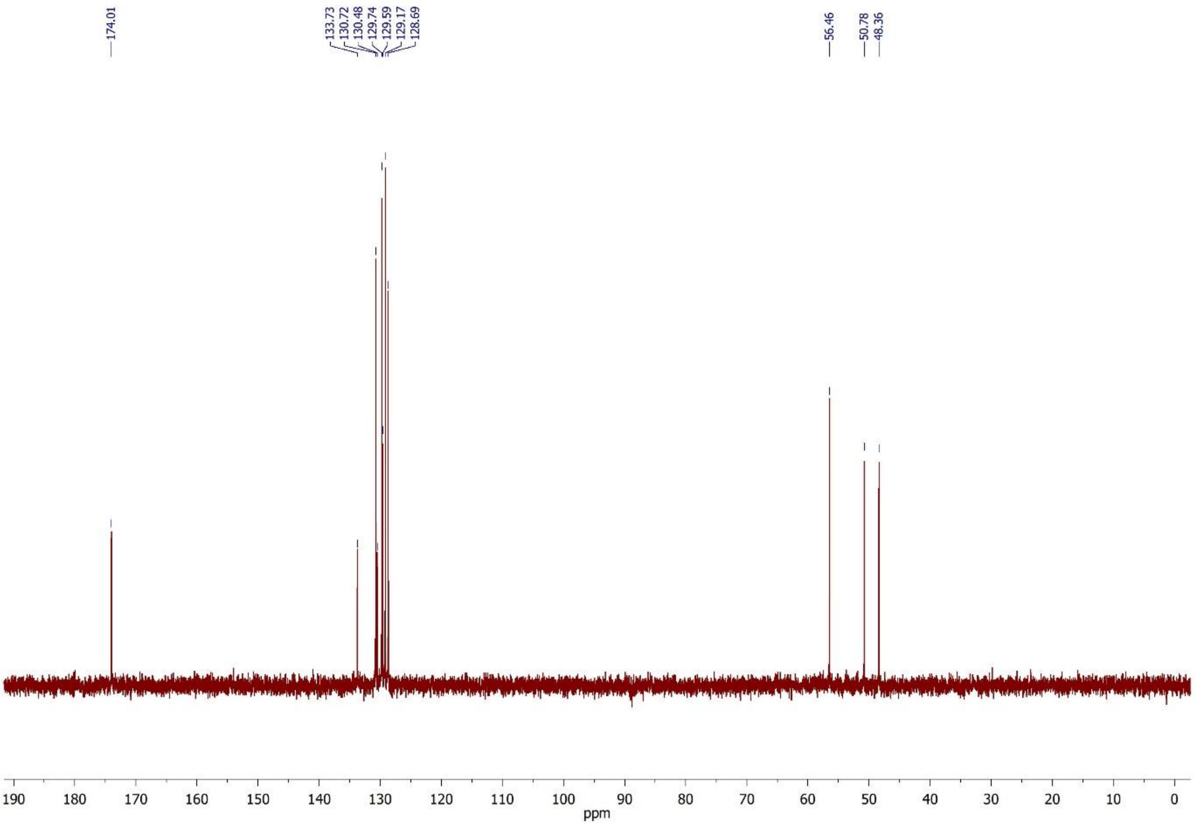

Figure S22 (b). ^{13}C NMR Spectrum of **7** (D_2O, δ, ppm)

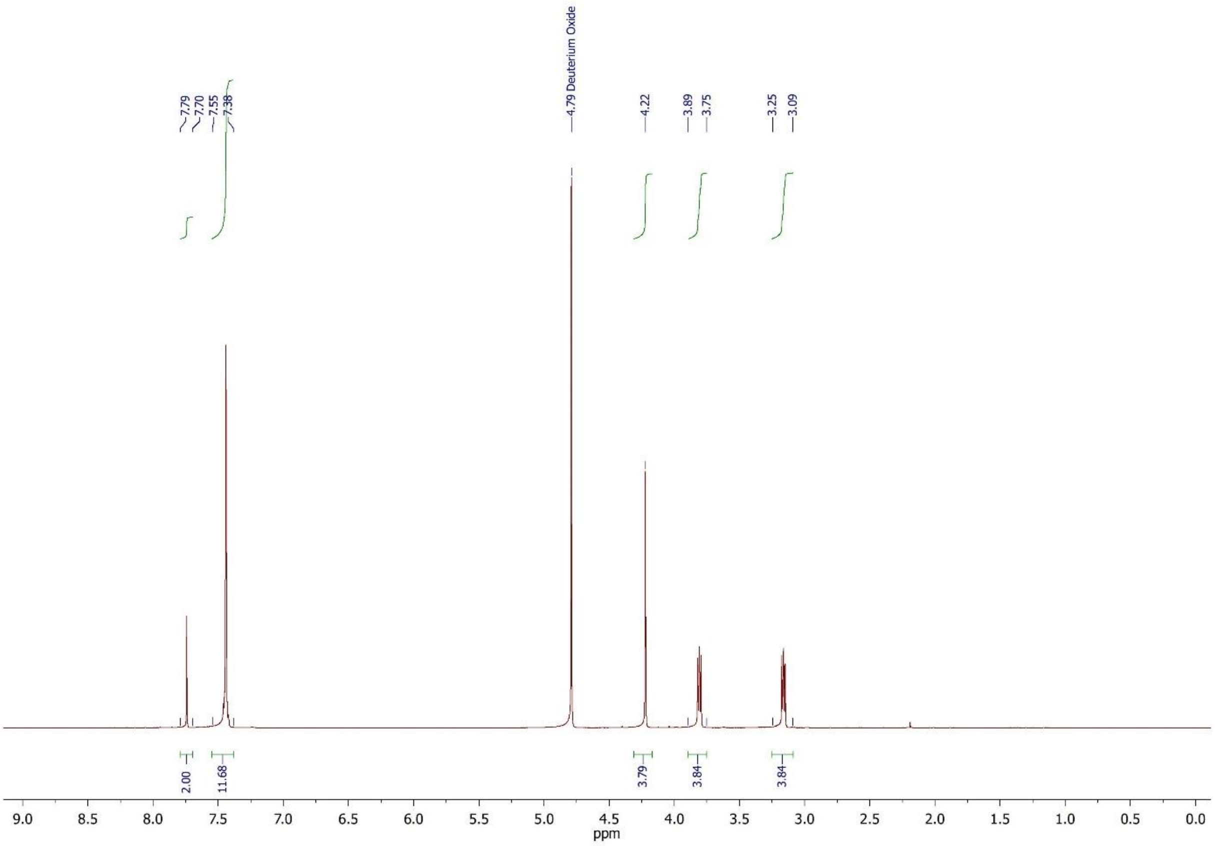

Figure S23 (a).^{1}H NMR Spectrum of **8** (D_2O, δ, ppm, *J*, Hz)

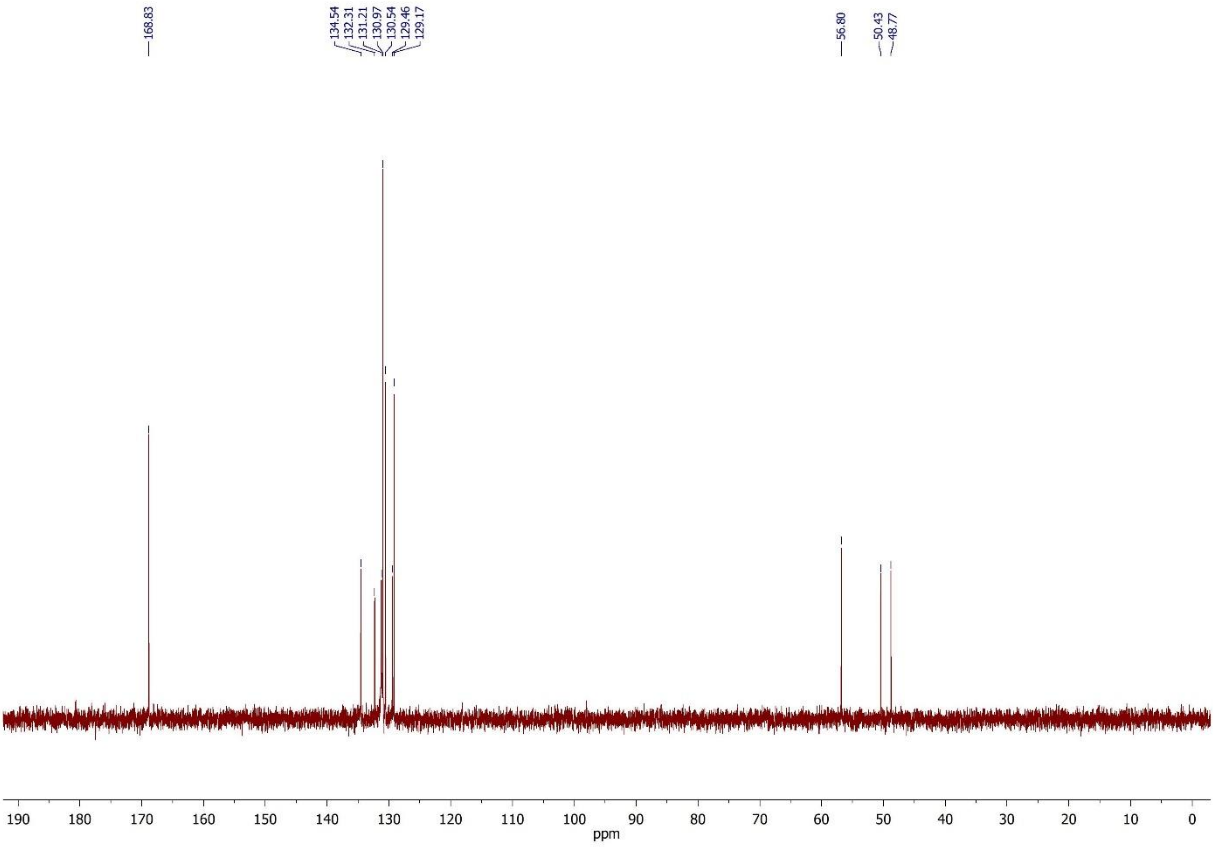

Figure S23 (b). ^{13}C NMR Spectrum of **8** (D_2O, δ, ppm)

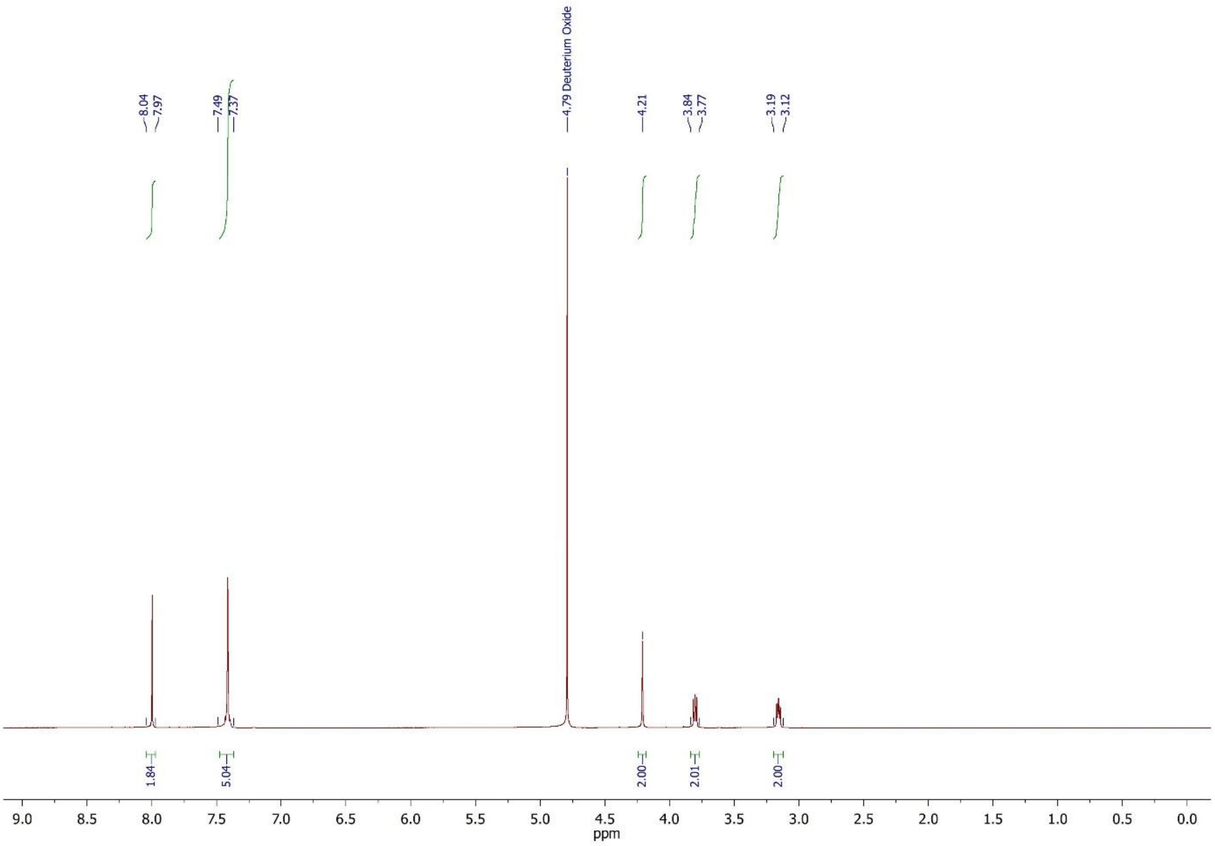

Figure S24 (a).^{1}H NMR Spectrum of **9** (D_2O, δ, ppm, *J*, Hz)

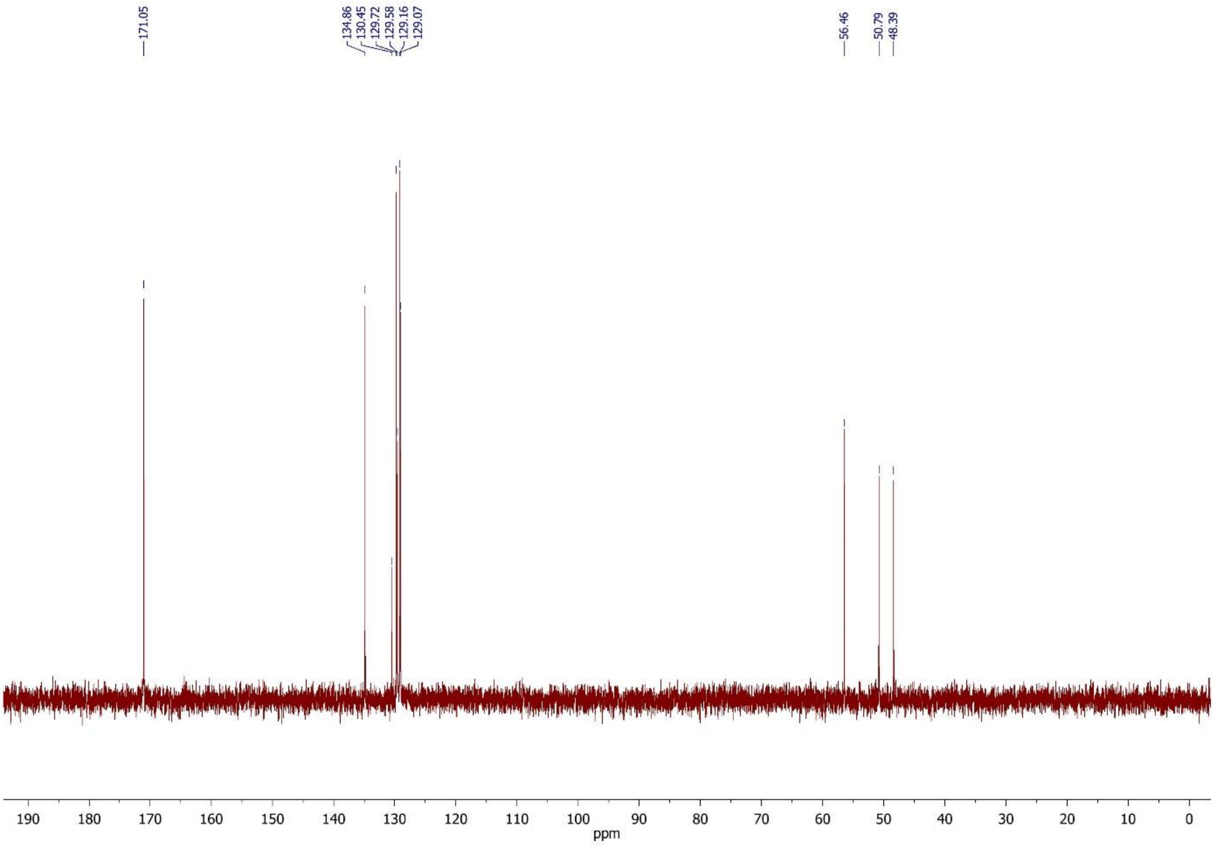

Figure S24 (b). ^{13}C NMR Spectrum of **9** (D_2O, δ, ppm)

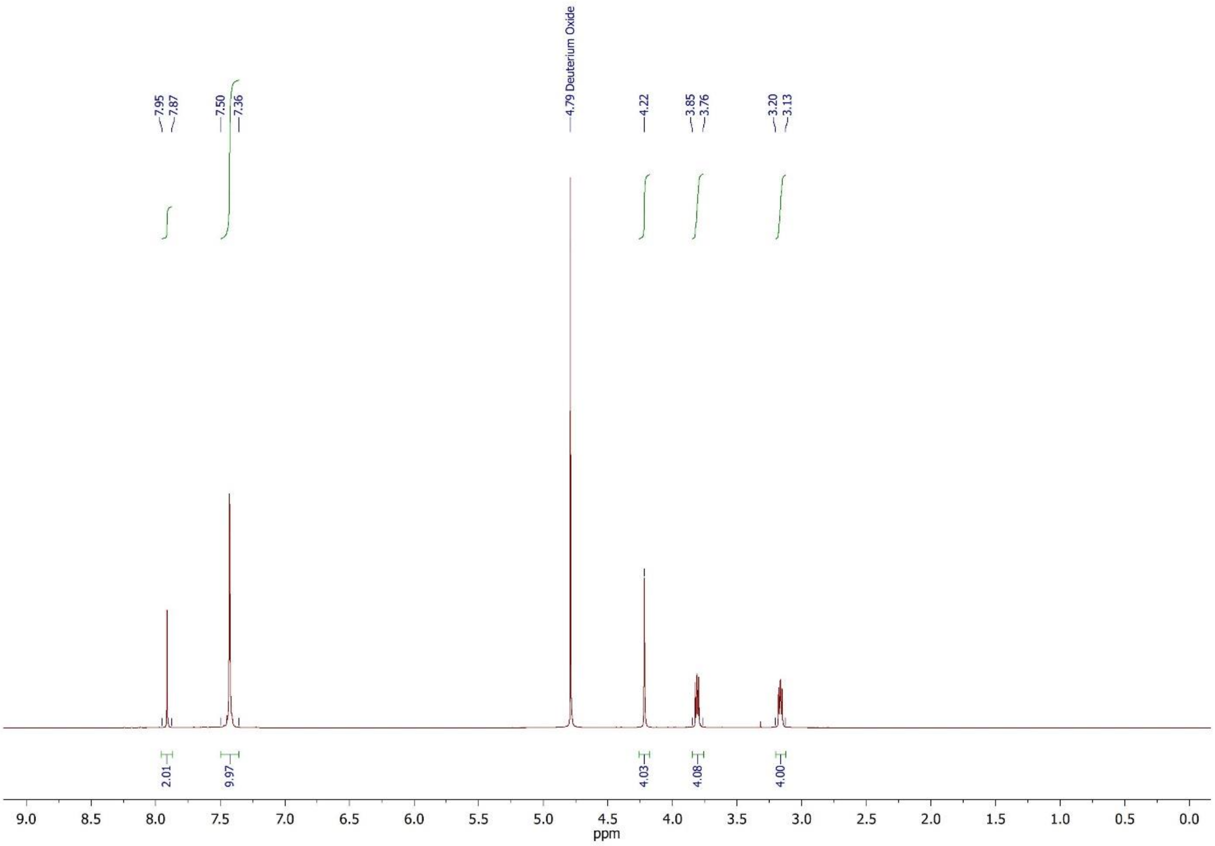

Figure S25 (a).^{1}H NMR Spectrum of **10** (D_2O, δ, ppm, *J*, Hz)

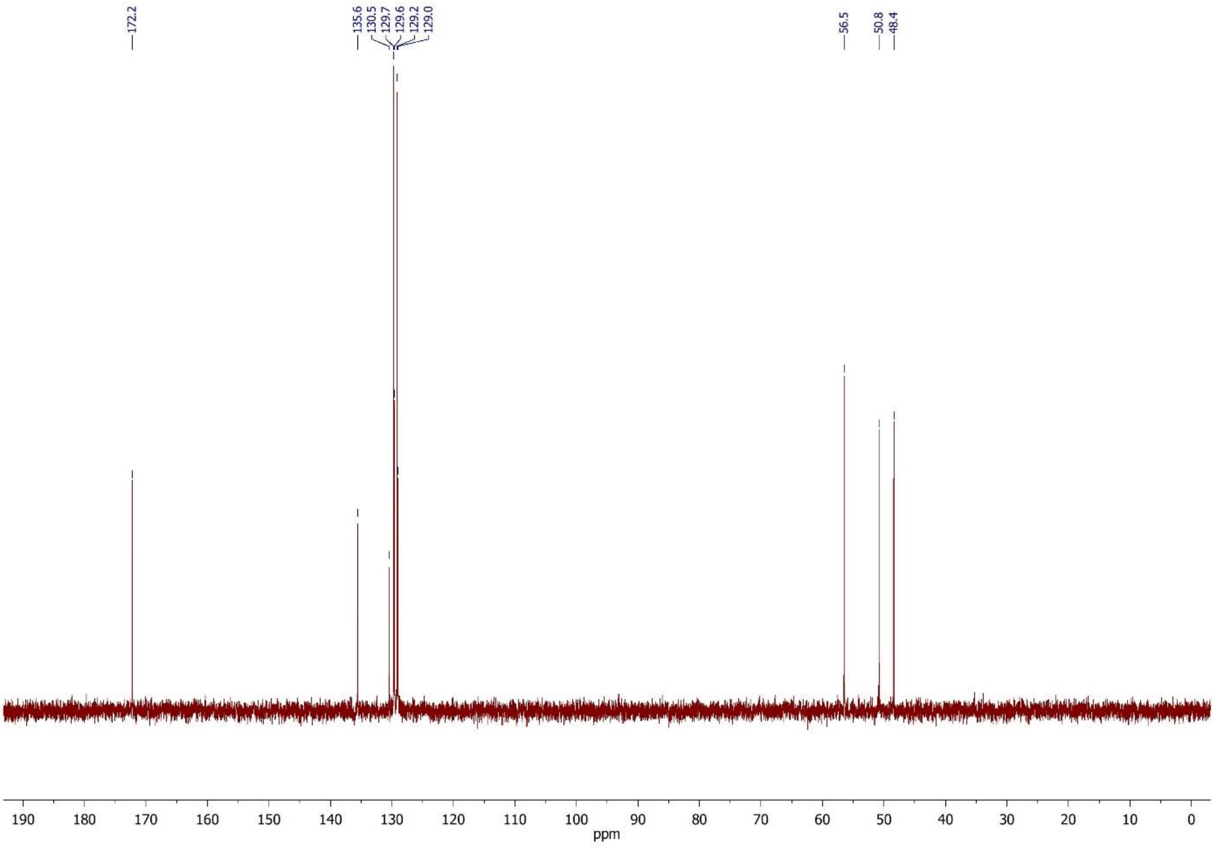

Figure S25 (b). ^{13}C NMR Spectrum of **10** (D_2O, δ, ppm)

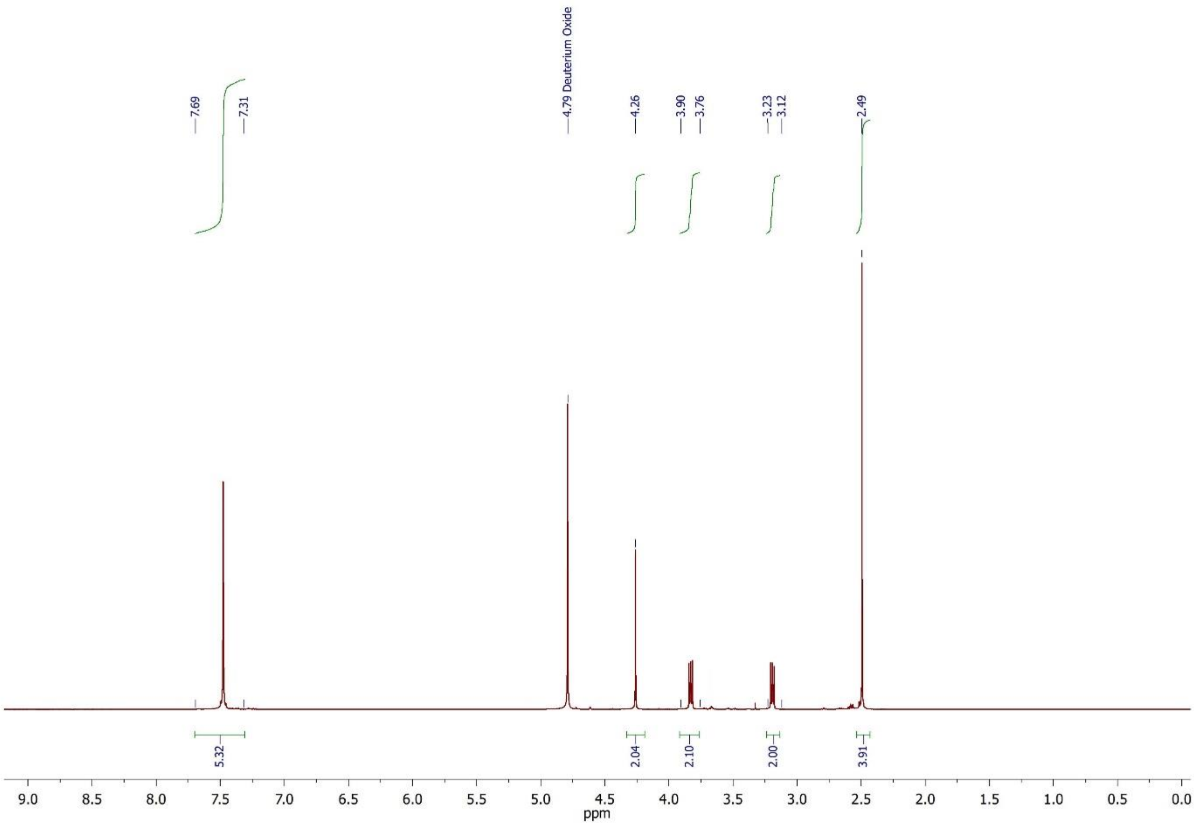

Figure S26 (a).^{1}H NMR Spectrum of **11** (D_2O, δ, ppm, *J*, Hz)

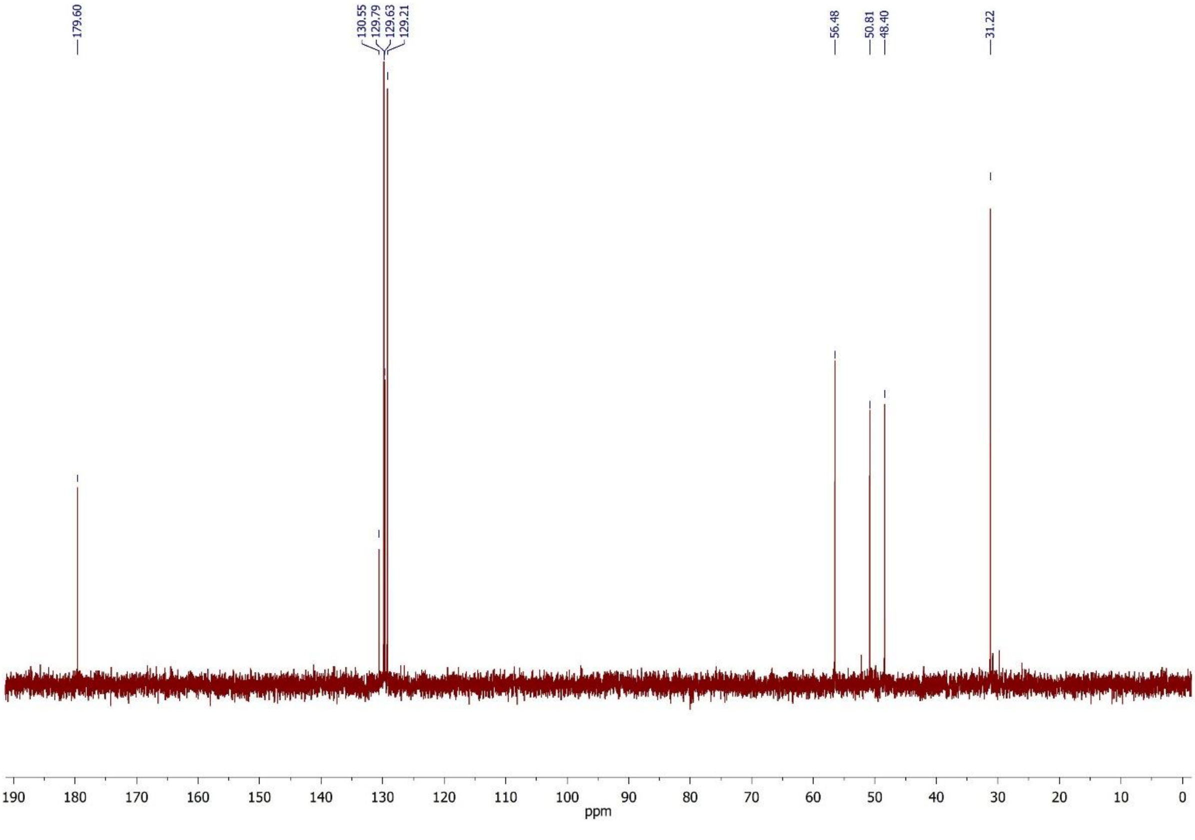

Figure S26 (b). ^{13}C NMR Spectrum of **11** (D_2O, δ, ppm)

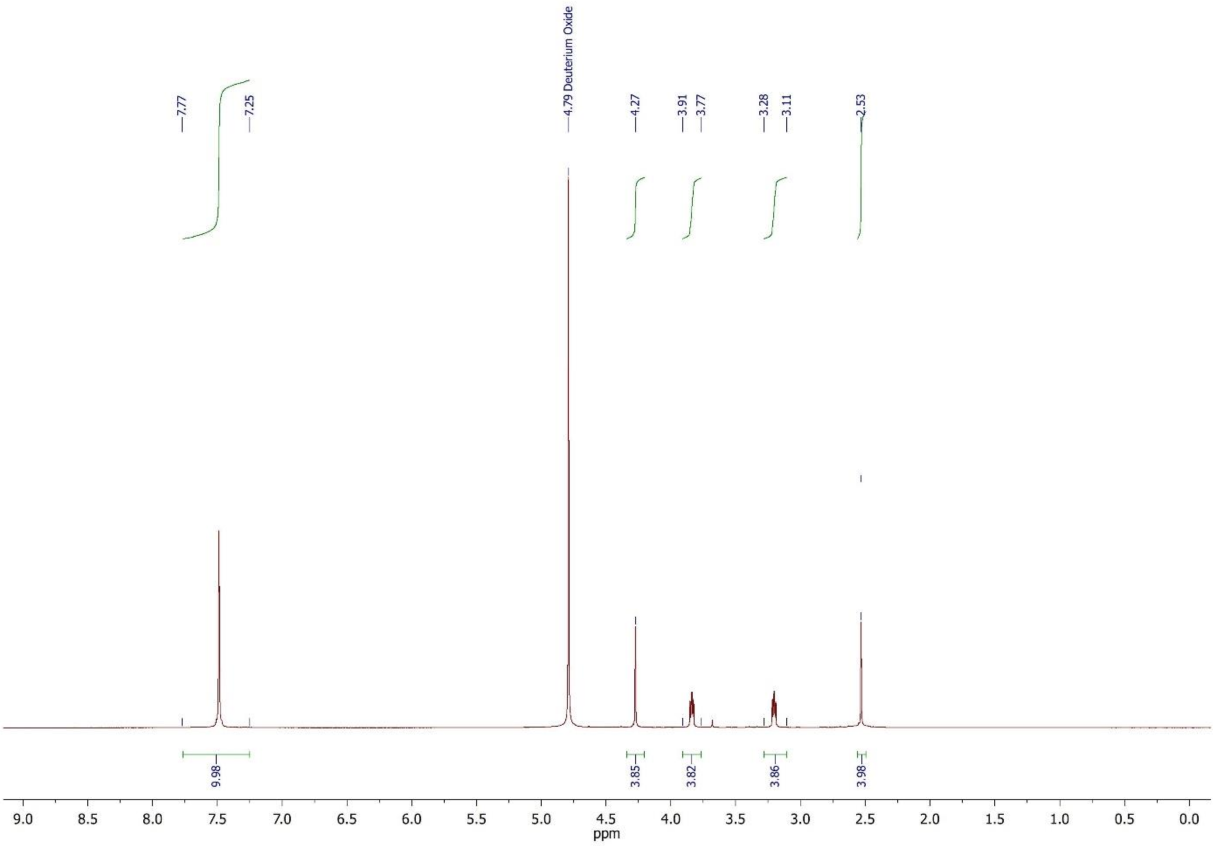

Figure S27 (a).^{1}H NMR Spectrum of **12** (D_2O, δ, ppm, *J*, Hz)

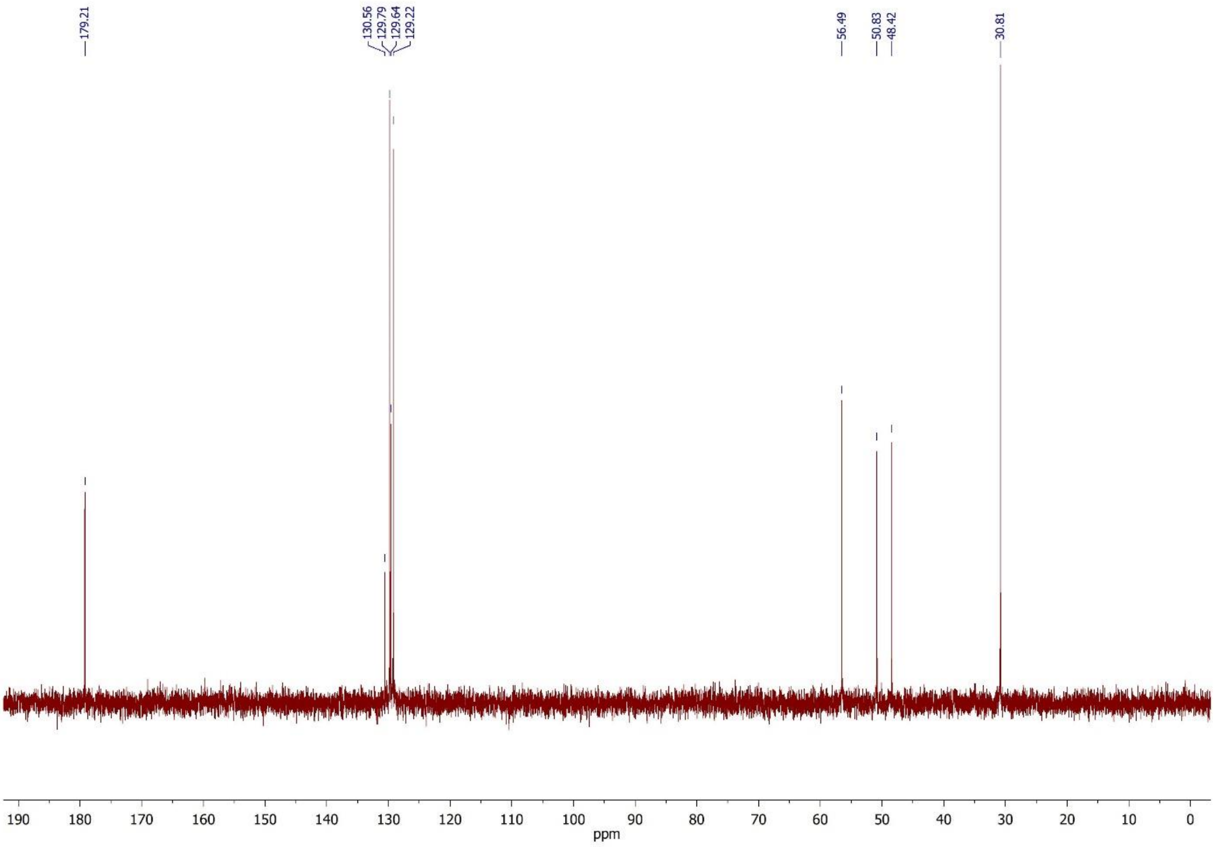

Figure S27 (b). ^{13}C NMR Spectrum of **12** (D_2O, δ, ppm)

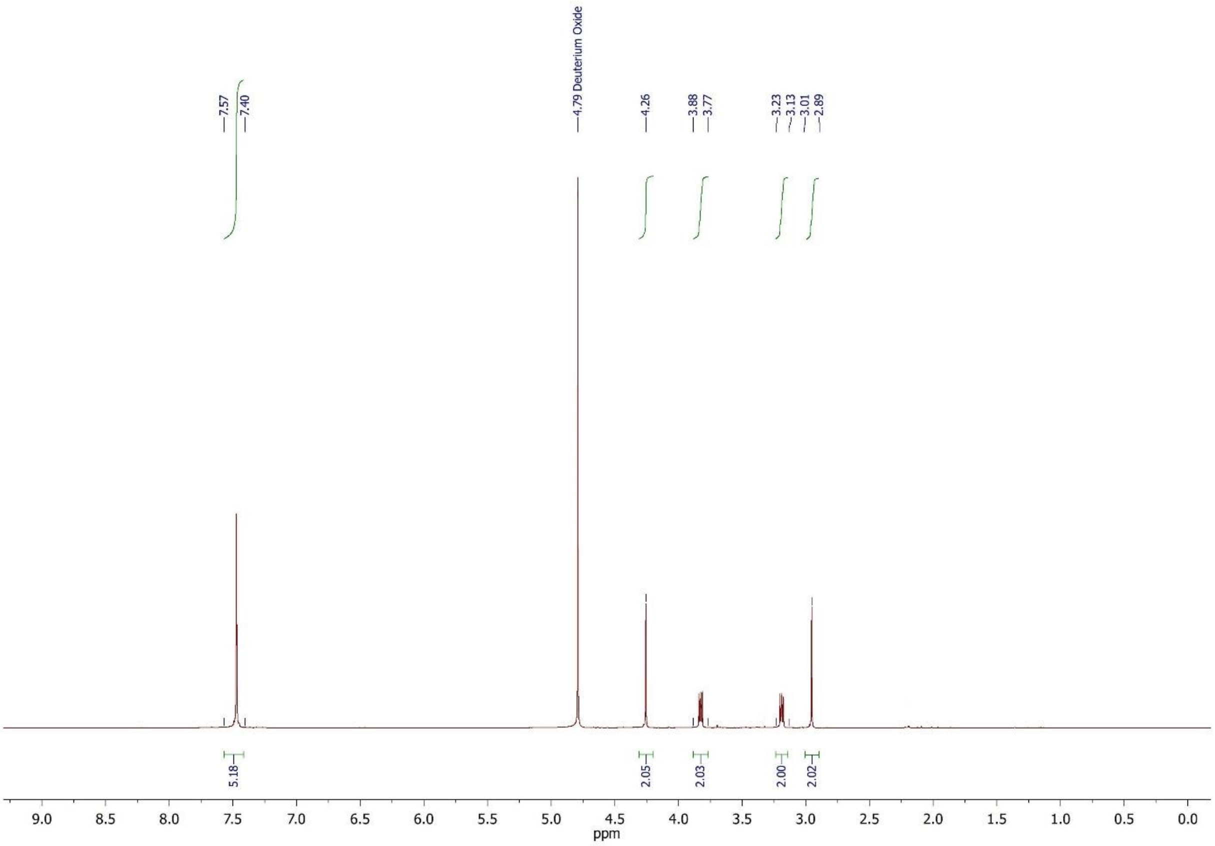

Figure S28 (a).^{1}H NMR Spectrum of **13** (D_2O, δ, ppm, *J*, Hz)

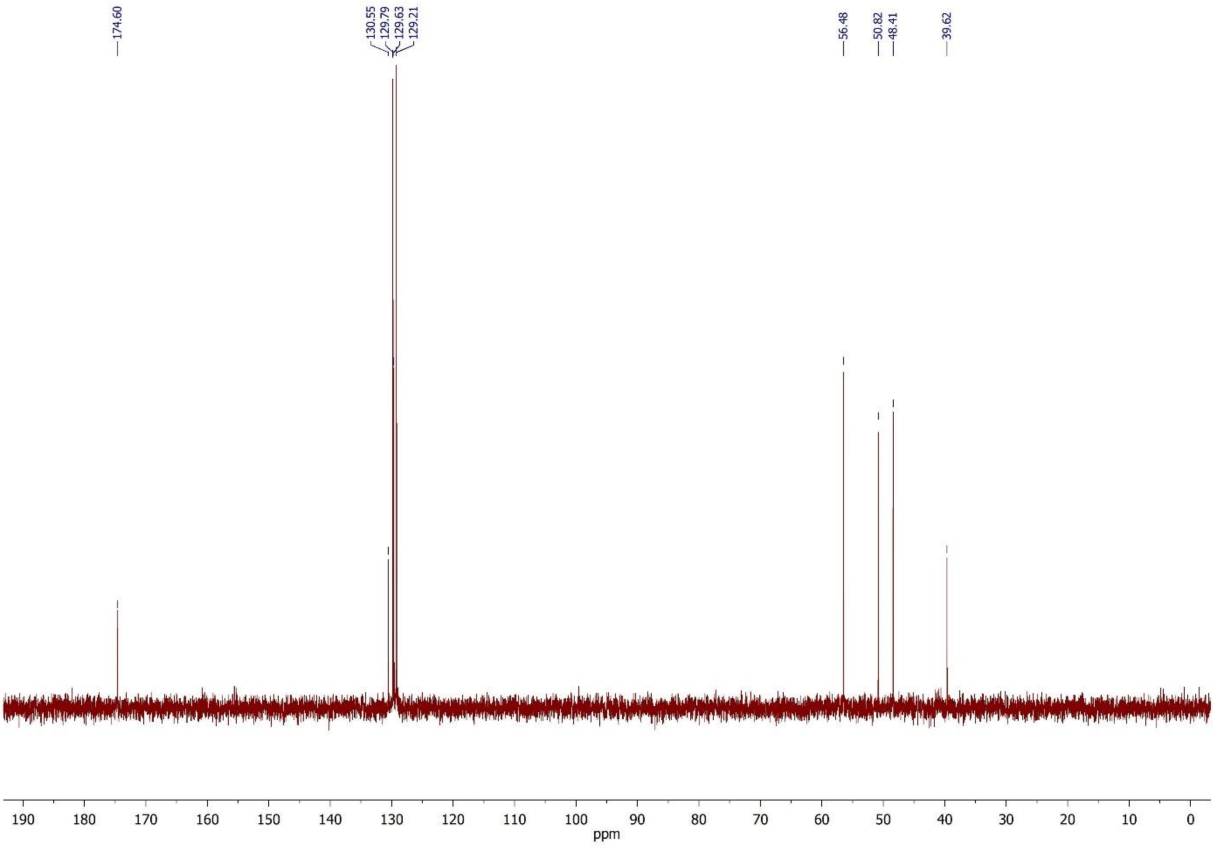

Figure S28 (b). ^{13}C NMR Spectrum of **13** (D_2O, δ, ppm)

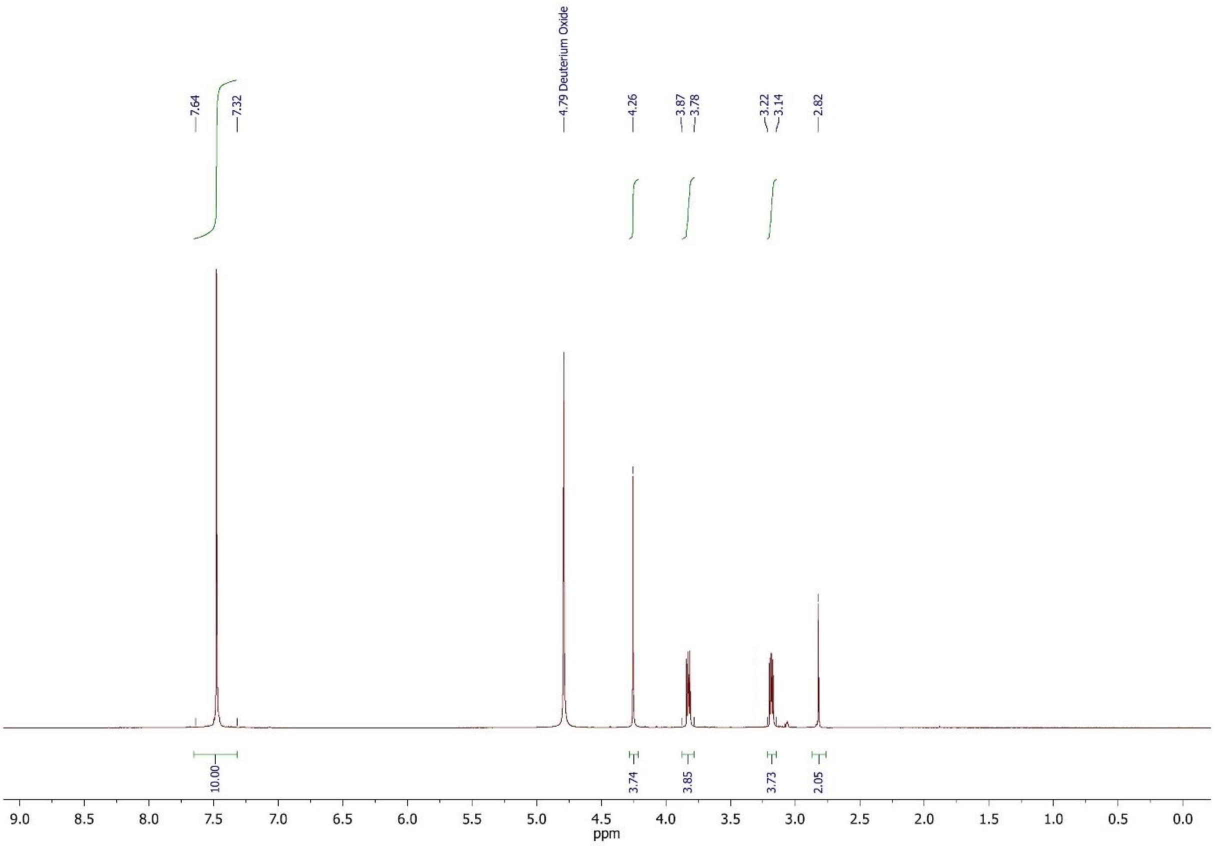

Figure S29 (a).^{1}H NMR Spectrum of **14** (D_2O, δ, ppm, *J*, Hz)

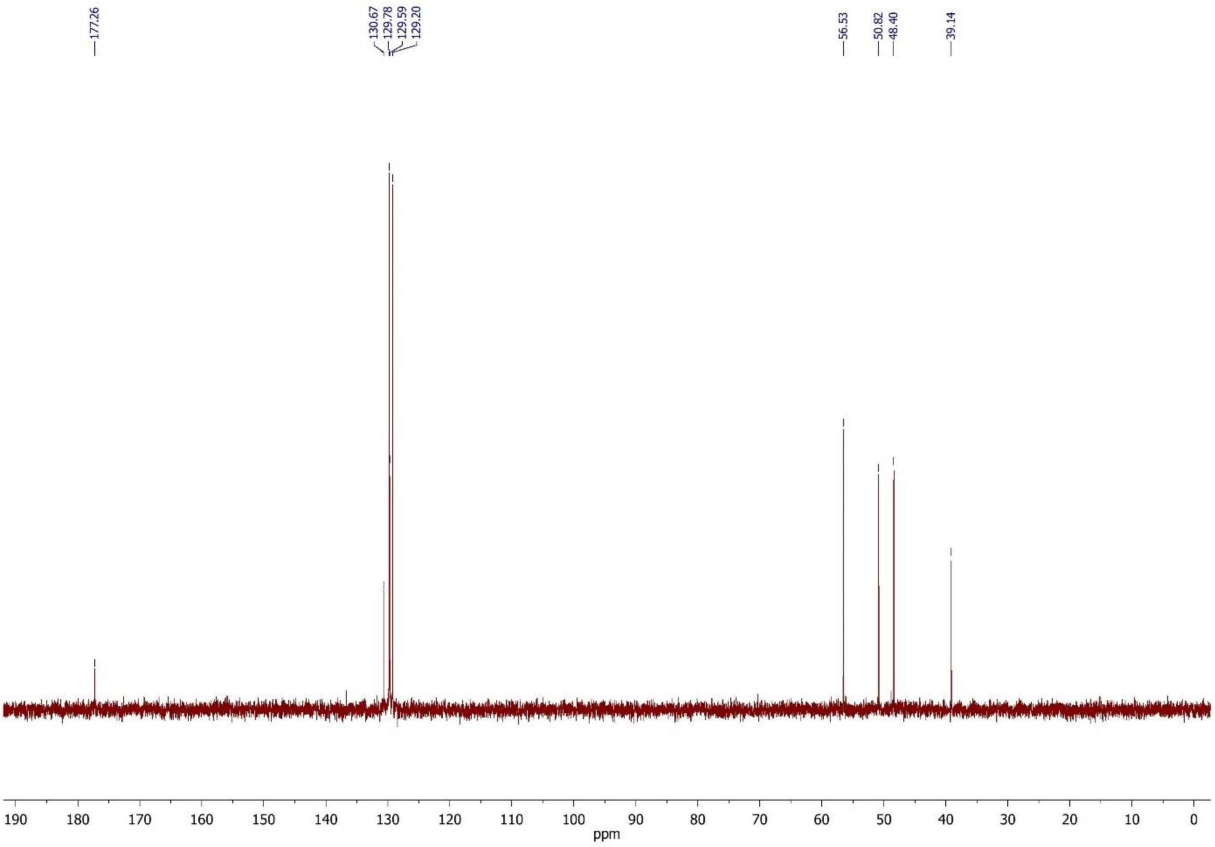

Figure S29 (b). ^{13}C NMR Spectrum of **14** (D_2O, δ, ppm)

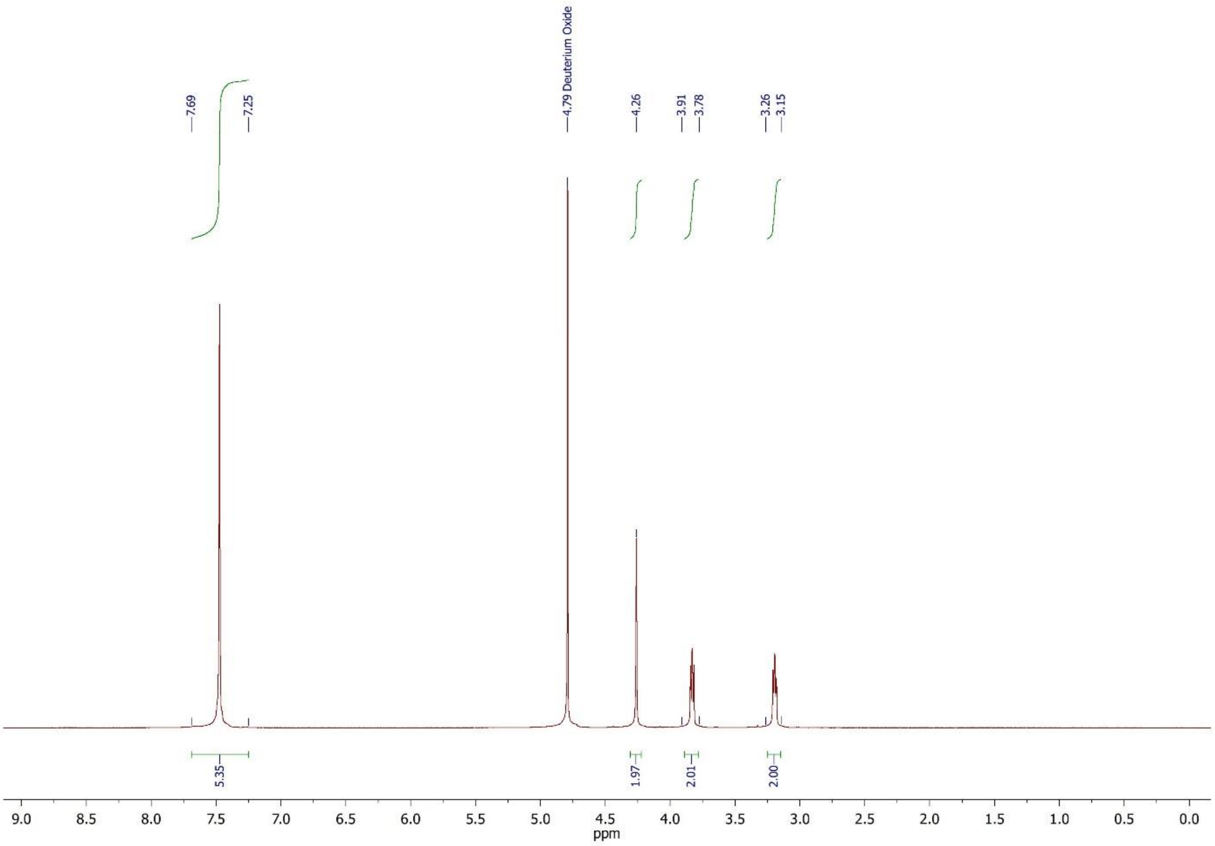

Figure S30 (a).^{1}H NMR Spectrum of **15** (D_2O, δ, ppm, *J*, Hz)

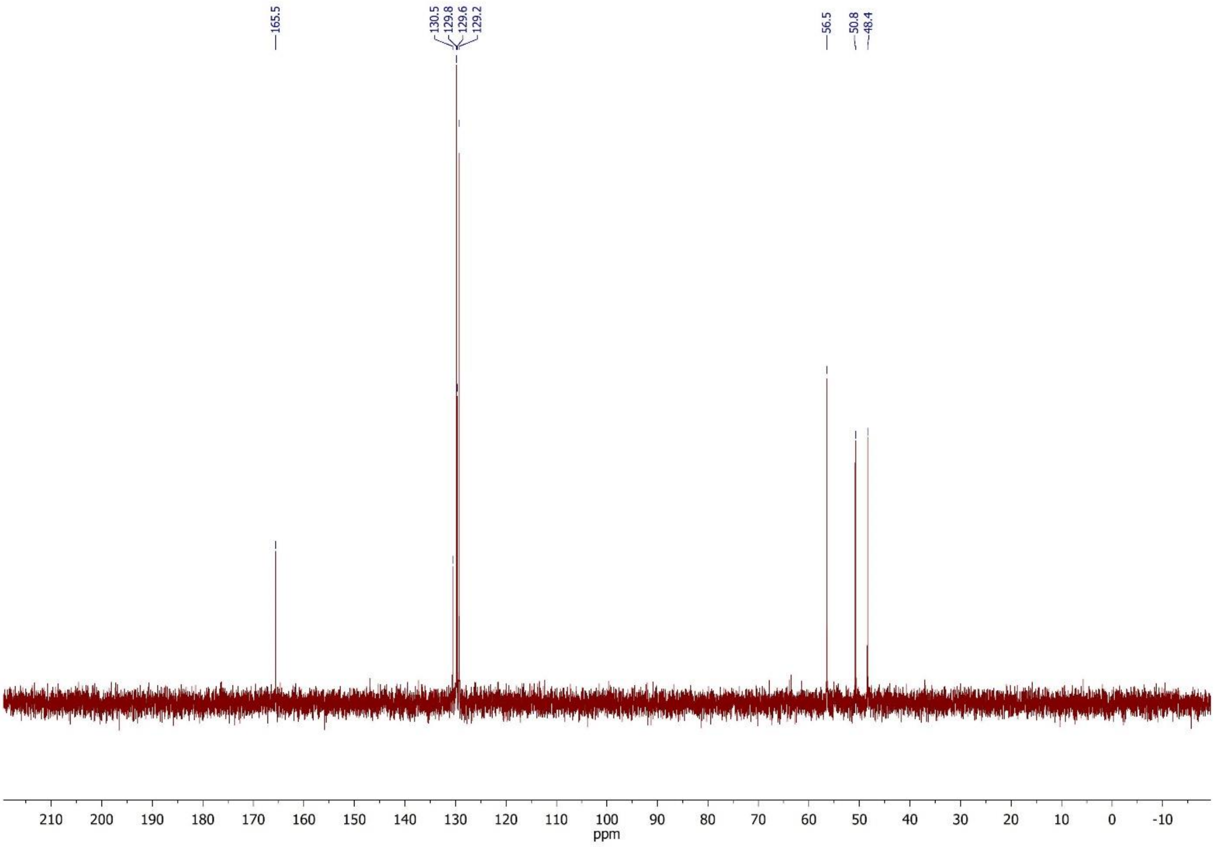

Figure S30 (b). ^{13}C NMR Spectrum of **15** (D_2O, δ, ppm)

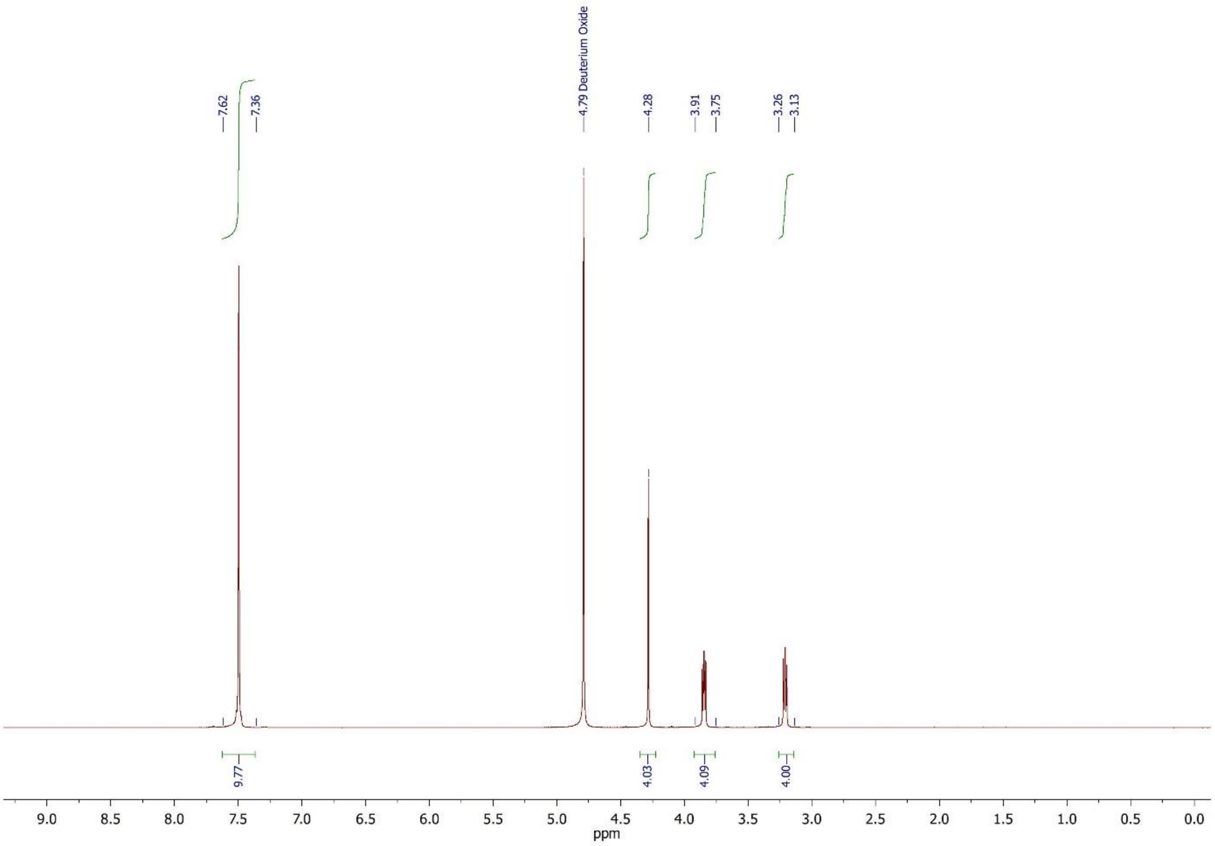

Figure S31 (a).^{1}H NMR Spectrum of **16** (D_2O, δ, ppm, *J*, Hz)

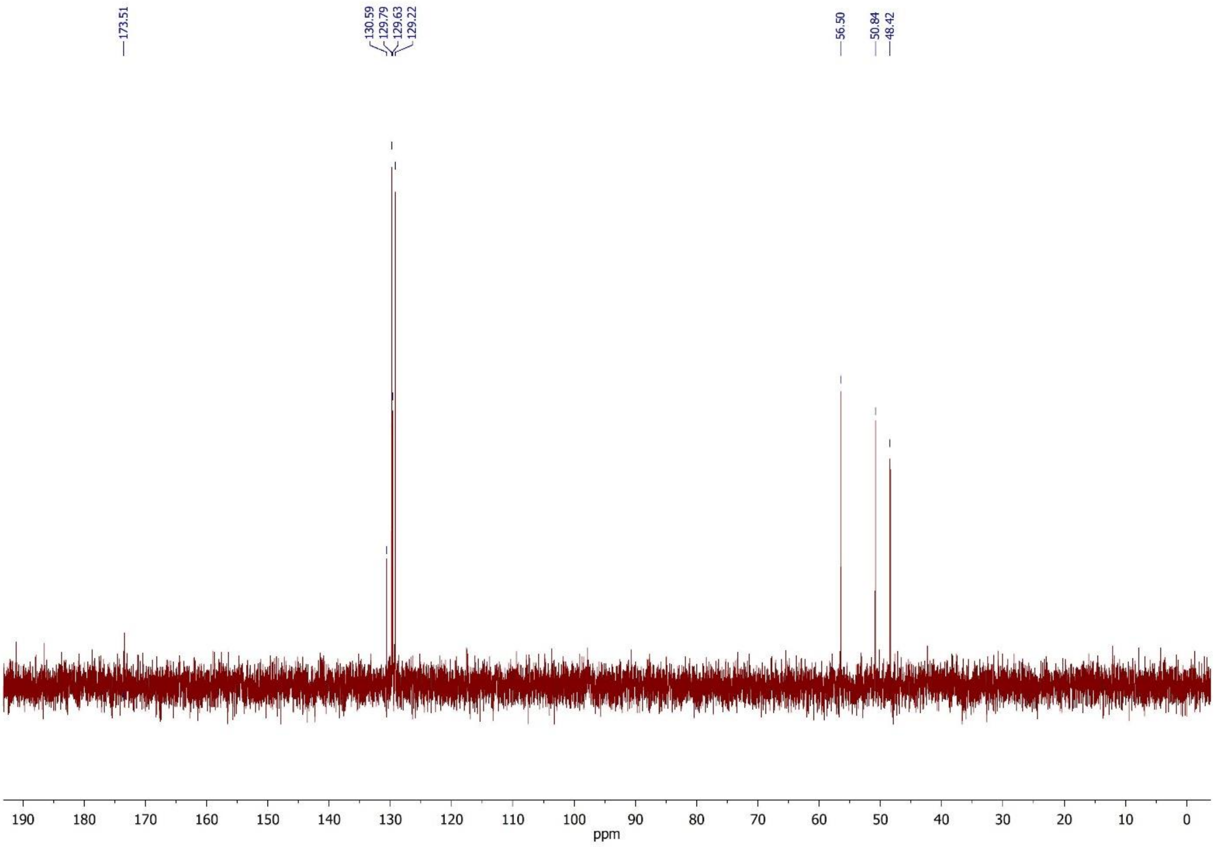

Figure S31 (b). ^{13}C NMR Spectrum of **16** (D_2O, δ, ppm)

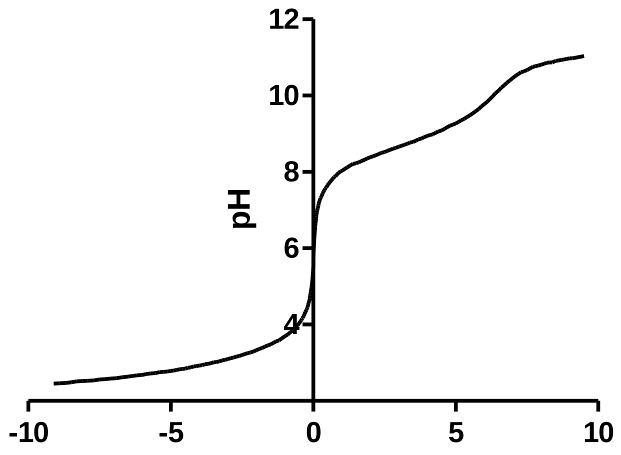

Figure S32. pH profile of buffer **3**

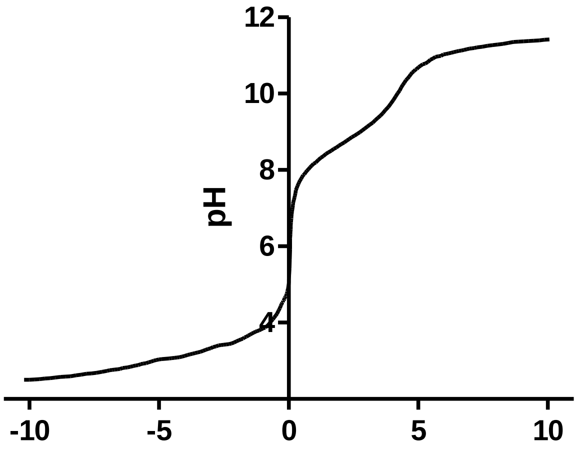

Figure S33. pH profile of buffer **4**

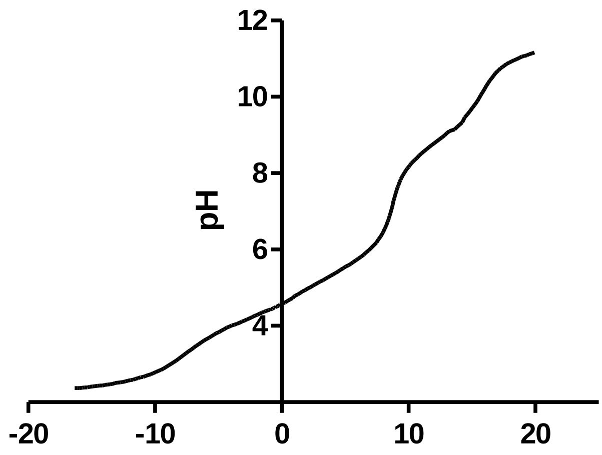

Figure S34. pH profile of buffer **12**

NMR (^{1}H, ^{13}C) spectroscopy data of BEA salts 1-16:

1) N-benzylethanolammonium benzoate (1)

^{1}H NMR spectrum of **1** (D_2O, ppm): 3.12-3.25 m (2H, $C_6H_5-CH_2-NH_2^+-C\underline{H_2}-CH_2-OH$); 3.77-3.88 m (2H, $C_6H_5-CH_2-NH_2^+-CH_2-C\underline{H_2}-OH$); 4.25 s (2H, $C_6H_5-C\underline{H_2}-NH_2^+-CH_2-CH_2-OH$); 7.39-7.59 m ($8H_{Ar}$); 7.81-7.94 m ($2H_{Ar}$).

^{13}C NMR spectrum of **1** (D_2O, ppm): 48.4 ($C_6H_5-CH_2-NH_2^+-\underline{C}H_2-CH_2-OH$); 50.8 ($C_6H_5-\underline{C}H_2-NH_2^+-CH_2-CH_2-OH$); 56.5 ($C_6H_5-CH_2-NH_2^+-CH_2-\underline{C}H_2-OH$); 128.3 ($\underline{C}_{Ar}H$); 128.8 ($\underline{C}_{Ar}H$); 129.2 ($\underline{C}_{Ar}H$); 129.6 ($\underline{C}_{Ar}H$); 129.8 ($\underline{C}_{Ar}H$); 130.5 ($\underline{C}_{Ar}$); 131.5 ($\underline{C}_{Ar}H$); 135.3($\underline{C}_{Ar}$); 175.1 ($\underline{C}O_2^-$).

2) N-benzylethanolammonium cinnamate (2)

^{1}H NMR spectrum of **2** (D_2O, ppm): 3.07-3.20 m (2H, $C_6H_5-CH_2-NH_2^+-C\underline{H_2}-CH_2-OH$); 3.72-3.84 m (2H, $C_6H_5-CH_2-NH_2^+-CH_2-C\underline{H_2}-OH$); 4.19 s (2H, $C_6H_5-C\underline{H_2}-NH_2^+-CH_2-CH_2-OH$); 6.37-6.52 m (1H, $C_6H_5-CH=C\underline{H}-CO_2^-$); 7.29-7.34 m (1H, $C_6H_5-CH=C\underline{H}-CO_2$); 7.36-7.47 m ($8H_{Ar}$); 7.51-7.56 м ($2H_{Ar}$).

^{13}C NMR spectrum of **2** (D_2O, ppm): 48.4 ($C_6H_5-CH_2-NH_2^+-\underline{C}H_2-CH_2-OH$); 50.8 ($C_6H_5-\underline{C}H_2-NH_2^+-CH_2-CH_2-OH$); 56.5 ($C_6H_5-CH_2-NH_2^+-CH_2-\underline{C}H_2-OH$); 123.9 ($C_6H_5CH=\underline{C}H-CO_2^-$); 127.6 ($\underline{C}_{Ar}H$); 128.9 ($\underline{C}_{Ar}H$); 129.2 ($\underline{C}_{Ar}H$); 129.6 ($\underline{C}_{Ar}H$); 129.7 ($\underline{C}_{Ar}H$); 130.5 ($\underline{C}_{Ar}$); 135.0 ($\underline{C}_{Ar}$); 140.8 ($C_6H_5\underline{C}H=CH-CO_2^-$); 175.5 ($C_6H_5-CH=CH-\underline{C}O_2^-$).

3) N-benzylethanolammonium salicylate (3)

^{1}H NMR spectrum of **3** (D_2O, ppm): 3.07-3.19 m (2H, $C_6H_5-CH_2-NH_2^+-C\underline{H_2}-CH_2-OH$); 3.73-3.86 m (2H, $C_6H_5-CH_2-NH_2^+-CH_2-C\underline{H_2}-OH$); 4.19 s (2H, $C_6H_5-C\underline{H_2}-NH_2^+-CH_2-CH_2-OH$); 6.79-6.97 m ($2H_{Ar}$); 7.30-7.49 m ($6H_{Ar}$); 7.67-7.82 m ($1H_{Ar}$).

^{13}C NMR spectrum of **3** (D_2O, ppm): 48.4 ($C_6H_5-CH_2-NH_2^+-\underline{C}H_2-CH_2-OH$); 50.8 ($C_6H_5-\underline{C}H_2-NH_2^+-CH_2-CH_2-OH$); 56.5 ($C_6H_5-CH_2-NH_2^+-CH_2-\underline{C}H_2-OH$); 116.2 ($\underline{C}_{Ar}H$); 118.0 ($\underline{C}_{Ar}$); 119.3 ($\underline{C}_{Ar}H$); 129.2 ($\underline{C}_{Ar}H$); 129.6 ($\underline{C}_{Ar}H$); 129.7 ($\underline{C}_{Ar}H$); 130.4 ($\underline{C}_{Ar}H$); 130.4 ($\underline{C}_{Ar}H$); 133.9 ($\underline{C}_{Ar}$); 159.5 ($\underline{C}_{Ar}$); 175.5 ($\underline{C}O_2^-$).

4) N-benzylethanolammonium 2-methylpnenoxyacetate (5)

^{1}H NMR spectrum of **5** (D_2O, ppm): 2.22 c (3H, o-$C\underline{H_3}-C_6H_4-O-CH_2-CO_2^-$); 3.06-3.27 m (2H, C_6H_5- $CH_2-NH_2^+-C\underline{H_2}-CH_2-OH$); 3.70-3.92 m (2H, C_6H_5- CH_2-

NH_2^+-CH_2-C$\underline{H_2}$-OH); 4.22 s (2H, C_6H_5-C$\underline{H_2}$-NH_2^+-CH_2-CH_2-OH); 4.43 s (2H, *o*-CH_3-C_6H_4-O-C$\underline{H_2}$-CO_2^-); 6.68-6.82 m (1H_{Ar}); 6.84-6.98 m (1H_{Ar}); 7.07-7.27 m (2H_{Ar}); 7.35-7.57 m (5H_{Ar}).

^{13}C NMR spectrum of **5** (D_2O, ppm): 15.5 (*o*-$\underline{C}H_3$-C_6H_4-O-CH_2- CO_2^-); 48.4 (C_6H_5-CH_2-NH_2^+-$\underline{C}H_2$-CH_2-OH); 50.8 (C_6H_5-$\underline{C}H_2$-NH_2^+-CH_2-CH_2-OH); 56.5 (C_6H_5-CH_2-NH_2^+-CH_2-$\underline{C}H_2$-OH); 67.0 (*o*-CH_3-C_6H_4-O-$\underline{C}H_2$-CO_2^-); 111.5 ($\underline{C}_{Ar}$H); 121.1 ($\underline{C}_{Ar}$H); 126.8 ($\underline{C}_{Ar}$); 127.0 ($\underline{C}_{Ar}$H); 129.2 ($\underline{C}_{Ar}$H); 129.6 ($\underline{C}_{Ar}$H); 129.8 ($\underline{C}_{Ar}$H); 130.5 ($\underline{C}_{Ar}$); 130.9 ($\underline{C}_{Ar}$H); 156.0 ($\underline{C}_{Ar}$); 177.0 ($\underline{C}O_2^-$).

5) N-benzylethanolammonium 4-chloro-2-methylpnenoxyacetate (6)

^{1}H NMR spectrum of **6** (D_2O, ppm): 2.19 s (3H, (Cl)(C$\underline{H_3}$)C_6H_4-O-CH_2- CO_2^-); 3.08-3.22 m (2H, C_6H_5- CH_2-NH_2^+-C$\underline{H_2}$-CH_2-OH); 3.75-3.86 m (2H, C_6H_5- CH_2-NH_2^+-CH_2-C$\underline{H_2}$-OH); 4.23 s (2H, C_6H_5-C$\underline{H_2}$-NH_2^+-CH_2-CH_2-OH); 4.42 s (2H, (Cl)(CH_3)C_6H_4-O-C$\underline{H_2}$-CO_2^-); 6.60-6.74 m (1H_{Ar}); 7.05-7.21 m (2H_{Ar}); 7.33-7.54 m (5H_{Ar}).

^{13}C NMR spectrum of **6** (D_2O, ppm): 15.4 ((Cl)($\underline{C}H_3$)C_6H_4-O-CH_2-CO_2^-); 48.4 (C_6H_5-CH_2-NH_2^+-$\underline{C}H_2$-CH_2-OH); 50.8 (C_6H_5-$\underline{C}H_2$-NH_2^+-CH_2-CH_2-OH); 56.5 (C_6H_5-CH_2-NH_2^+-CH_2-$\underline{C}H_2$-OH); 67.1 ((Cl)(CH_3)C_6H_4-O-$\underline{C}H_2$-CO_2^-); 112.6 ($\underline{C}_{Ar}$H); 124.9 ($\underline{C}_{Ar}$); 126.3 ($\underline{C}_{Ar}$H); 128.9 ($\underline{C}_{Ar}$); 129.2 ($\underline{C}_{Ar}$H); 129.6 ($\underline{C}_{Ar}$H); 129.8 ($\underline{C}_{Ar}$H); 130.3 ($\underline{C}_{Ar}$H); 130.5 ($\underline{C}_{Ar}$); 154.6 ($\underline{C}_{Ar}$); 176.7 ($\underline{C}O_2^-$).

6) N-benzylethanolammonium hydrogen phthalate (7)

^{1}H NMR spectrum of **7** (D_2O, ppm): 3.08-3.19 m (2H, C_6H_5-CH_2-NH_2^+-C$\underline{H_2}$-CH_2-OH); 3.68-3.87 m (2H, C_6H_5- CH_2-NH_2^+-CH_2-C$\underline{H_2}$-OH); 4.20 s (2H, C_6H_5-C$\underline{H_2}$-NH_2^+-CH_2-CH_2-OH); 7.37-7.47 m (5H_{Ar}); 7.47-7.55 m (2H_{Ar}); 7.65-7.70 m (2H_{Ar}).

^{13}C NMR spectrum of **7** (D_2O, ppm): 48.4 (C_6H_5-CH_2-NH_2^+-$\underline{C}H_2$-CH_2-OH); 50.8 (C_6H_5-$\underline{C}H_2$-NH_2^+-CH_2-CH_2-OH); 56.5 (C_6H_5-CH_2-NH_2^+-CH_2-$\underline{C}H_2$-OH); 128.7 ($\underline{C}_{Ar}$H); 129.2 ($\underline{C}_{Ar}$H); 129.6 ($\underline{C}_{Ar}$H); 129.7 ($\underline{C}_{Ar}$H); 130.5 ($\underline{C}_{Ar}$); 130.7 ($\underline{C}_{Ar}$H); 133.7 ($\underline{C}_{Ar}$); 174.0 ($HO_2\underline{C}$-C_6H_4-$\underline{C}O_2^-$).

7) N-benzylethanolammonium phthalate (8)

^{1}H NMR spectrum of **8** (D_2O, ppm): 3.09-3.25 m (4H, C_6H_5-CH_2-NH_2^+-$C\underline{H_2}$-CH_2-OH); 3.75-3.89 m (4H, C_6H_5- CH_2-NH_2^+-CH_2-$C\underline{H_2}$-OH); 4.22 s (4H, C_6H_5-$C\underline{H_2}$-NH_2^+-CH_2-CH_2-OH); 7.38-7.44 m (12H_{Ar}); 7.70-7.79 m (2H_{Ar}).

^{13}C NMR spectrum of **8** (D_2O, ppm): 48.8 (C_6H_5-CH_2-NH_2^+-$\underline{C}H_2$-CH_2-OH); 50.4 (C_6H_5-$\underline{C}H_2$-NH_2^+-CH_2-CH_2-OH); 56.8 (C_6H_5-CH_2-NH_2^+-CH_2-$\underline{C}H_2$-OH); 129.2 ($\underline{C}_{Ar}H$); 129.5 ($\underline{C}_{Ar}H$); 130.5 ($\underline{C}_{Ar}H$); 131.0 ($\underline{C}_{Ar}H$); 131.2 ($\underline{C}_{Ar}$); 132.3 ($\underline{C}_{Ar}H$); 134.5 ($\underline{C}_{Ar}$); 168.8 ($^-O_2\underline{C}$-C_6H_4-$\underline{C}O_2^-$).

8) N-benzylethanolammonium trihydrogen pyromellitate (9)

^{1}H NMR spectrum of **9** (D_2O, ppm): 3.12-3.19 m (2H, C_6H_5-CH_2-NH_2^+-$C\underline{H_2}$-CH_2-OH); 3.77-3.84 m (2H, C_6H_5-CH_2-NH_2^+-CH_2-$C\underline{H_2}$-OH); 4.21 s (2H, C_6H_5-$C\underline{H_2}$-NH_2^+-CH_2-CH_2-OH); 7.37-7.49 m (5H_{Ar}); 7.97-8.04 m (2H_{Ar}).

^{13}C NMR spectrum of **9** (D_2O, ppm): 48.4 (C_6H_5-CH_2-NH_2^+-$\underline{C}H_2$-CH_2-OH); 50.8 (C_6H_5-$\underline{C}H_2$-NH_2^+-CH_2-CH_2-OH); 56.5 (C_6H_5-CH_2-NH_2^+-CH_2-$\underline{C}H_2$-OH); 129.1 ($\underline{C}_{Ar}H$); 129.2 ($\underline{C}_{Ar}H$); 129.6 ($\underline{C}_{Ar}H$); 129.7 ($\underline{C}_{Ar}H$); 130.5 ($\underline{C}_{Ar}$); 134.9 ($\underline{C}_{Ar}$); 171.0 ($(HO_2\underline{C})_3$-C_6H_5-$\underline{C}O_2^-$).

9) N-benzylethanolammonium dihydrogen pyromellitate (10)

^{1}H NMR spectrum of **10** (D_2O, ppm): 3.13-3.20 m (4H, C_6H_5-CH_2-NH_2^+-$C\underline{H_2}$-CH_2-OH); 3.76-3.85 m (4H, C_6H_5-CH_2-NH_2^+-CH_2-$C\underline{H_2}$-OH); 4.22 s (2H, C_6H_5-$C\underline{H_2}$-NH_2^+-CH_2-CH_2-OH); 7.36-7.50 m (10H_{Ar}); 7.87-7.95 m (2H_{Ar}).

^{13}C NMR spectrum of **10** (D_2O, ppm): 48.4 (C_6H_5-CH_2-NH_2^+-$\underline{C}H_2$-CH_2-OH); 50.8 (C_6H_5-$\underline{C}H_2$-NH_2^+-CH_2-CH_2-OH); 56.5 (C_6H_5-CH_2-NH_2^+-CH_2-$\underline{C}H_2$-OH); 129.0 ($\underline{C}_{Ar}H$); 129.2 ($\underline{C}_{Ar}H$); 129.6 ($\underline{C}_{Ar}H$); 129.7 ($\underline{C}_{Ar}H$); 130.5 ($\underline{C}_{Ar}$); 135.6 ($\underline{C}_{Ar}$); 172.2 ($(HO_2\underline{C})_2$-C_6H_5-$(\underline{C}O_2^-)_2$).

10) N-benzylethanolammonium hydrogen succinate (11)

^{1}H NMR spectrum of **11** (D_2O, ppm): 2.49 s (4H, COOH-$C\underline{H_2}$-$C\underline{H_2}$-CO_2^-); 3.12-3.23 m (2H, C_6H_5- CH_2-NH_2^+-$C\underline{H_2}$-CH_2-OH); 3.76-3.90 m (2H, C_6H_5- CH_2-NH_2^+-CH_2-$C\underline{H_2}$-OH); 4.26 s (2H, C_6H_5-$C\underline{H_2}$-NH_2^+-CH_2-CH_2-OH); 7.31-7.69 m (5H_{Ar}).

^{13}C NMR spectrum of **11** (D_2O, ppm): 31.2 (COOH-$\underline{C}H_2$-$\underline{C}H_2$-CO_2^-); 48.4 (C_6H_5-CH_2-NH_2^+-$\underline{C}H_2$-CH_2-OH); 50.8 (C_6H_5-$\underline{C}H_2$-NH_2^+-CH_2-CH_2-OH); 56.5

($C_6H_5-CH_2-NH_2^+-CH_2-\underline{C}H_2-OH$); 129.2 ($\underline{C}_{Ar}H$); 129.6 ($\underline{C}_{Ar}H$); 129.8 ($\underline{C}_{Ar}H$); 130.6 ($\underline{C}_{Ar}$); 179.6 ($\underline{C}OOH-CH_2-CH_2-\underline{C}O_2^-$).

11) N-benzylethanolammonium succinate (12)

^{1}H NMR spectrum of **12** (D_2O, ppm): 2.53 s (4H, $^-O_2C-C\underline{H_2}-C\underline{H_2}-CO_2^-$); 3.11-3.28 m (4H, C_6H_5- $CH_2-NH_2^+-C\underline{H_2}-CH_2-OH$); 3.77-3.91 m (4H, C_6H_5- $CH_2-NH_2^+-CH_2-C\underline{H_2}-OH$); 4.27 s (4H, $C_6H_5-C\underline{H_2}-NH_2^+-CH_2-CH_2-OH$); 7.25-7.77 m ($10H_{Ar}$).

^{13}C NMR spectrum of **12** (D_2O, ppm): 30.8 ($^-O_2C-\underline{C}H_2-\underline{C}H_2-CO_2^-$); 48.4 ($C_6H_5-CH_2-NH_2^+-\underline{C}H_2-CH_2-OH$); 50.8 ($C_6H_5-\underline{C}H_2-NH_2^+-CH_2-CH_2-OH$); 56.5 ($C_6H_5-CH_2-NH_2^+-CH_2-\underline{C}H_2-OH$); 129.2 ($\underline{C}_{Ar}H$); 129.6 ($\underline{C}_{Ar}H$); 129.8 ($\underline{C}_{Ar}H$); 130.6 ($\underline{C}_{Ar}$); 179.2 ($^-O_2\underline{C}-CH_2-CH_2-\underline{C}O_2^-$).

12) N-benzylethanolammonium hydrogen malonate (13)

^{1}H NMR spectrum of **13** (D_2O, ppm): 2.89-3.01 m (2H, $HO_2C-C\underline{H_2}-CO_2^-$); 3.13-3.23 m (2H, $C_6H_5-CH_2-NH_2^+-C\underline{H_2}-CH_2-OH$); 3.77-3.88 m (2H, $C_6H_5-CH_2-NH_2^+-CH_2-C\underline{H_2}-OH$); 4.26 s (2H, $C_6H_5-C\underline{H_2}-NH_2^+-CH_2-CH_2-OH$); 7.40-7.67 m ($5H_{Ar}$).

^{13}C NMR spectrum of **13** (D_2O, ppm): 39.6 ($HO_2C-\underline{C}H_2-CO_2^-$); 48.4 ($C_6H_5-CH_2-NH_2^+-\underline{C}H_2-CH_2-OH$); 50.8 ($C_6H_5-\underline{C}H_2-NH_2^+-CH_2-CH_2-OH$); 56.5 ($C_6H_5-CH_2-NH_2^+-CH_2-\underline{C}H_2-OH$); 129.2 ($\underline{C}_{Ar}H$); 129.6 ($\underline{C}_{Ar}H$); 129.8 ($\underline{C}_{Ar}H$); 130.6 ($\underline{C}_{Ar}$); 174.6 ($HO_2\underline{C}-CH_2-\underline{C}O_2^-$).

13) N-benzylethanolammonium malonate (14)

^{1}H NMR spectrum of **14** (D_2O, ppm): 2.82 s (2H, $^-O_2C-C\underline{H_2}-CO_2^-$); 3.14-3.22 m (4H, $C_6H_5-CH_2-NH_2^+-C\underline{H_2}-CH_2-OH$); 3.78-3.87 m (4H, $C_6H_5-CH_2-NH_2^+-CH_2-C\underline{H_2}-OH$); 4.26 s (4H, $C_6H_5-C\underline{H_2}-NH_2^+-CH_2-CH_2-OH$); 7.32-7.64 m ($10H_{Ar}$).

^{13}C NMR spectrum of **14** (D_2O, ppm): 39.1 ($^-O_2C-\underline{C}H_2-CO_2^-$); 48.4 ($C_6H_5-CH_2-NH_2^+-\underline{C}H_2-CH_2-OH$); 50.8 ($C_6H_5-\underline{C}H_2-NH_2^+-CH_2-CH_2-OH$); 56.5 ($C_6H_5-CH_2-NH_2^+-CH_2-\underline{C}H_2-OH$); 129.2 ($\underline{C}_{Ar}H$); 129.6 ($\underline{C}_{Ar}H$); 129.8 ($\underline{C}_{Ar}H$); 130.6 ($\underline{C}_{Ar}$); 177.3 ($^-O_2\underline{C}-CH_2-\underline{C}O_2^-$).

14) N-benzylethanolammonium hydrogen oxalate (15)

^{1}H NMR spectrum of **15** (D_2O, ppm): 3.15-3.26 m (2H, C_6H_5- $CH_2\text{-}NH_2^+\text{-}C\underline{H_2}\text{-}CH_2\text{-}OH$); 3.78-3.91 m (2H, C_6H_5- $CH_2\text{-}NH_2^+\text{-}CH_2\text{-}C\underline{H_2}\text{-}OH$); 4.26 s (2H, $C_6H_5\text{-}C\underline{H_2}\text{-}NH_2^+\text{-}CH_2\text{-}CH_2\text{-}OH$); 7.25-7.69 m ($5H_{Ar}$).

^{13}C NMR spectrum of **15** (D_2O, ppm): 48.4 ($C_6H_5\text{-}CH_2\text{-}NH_2^+\text{-}\underline{C}H_2\text{-}CH_2\text{-}OH$); 50.8 ($C_6H_5\text{-}\underline{C}H_2\text{-}NH_2^+\text{-}CH_2\text{-}CH_2\text{-}OH$); 56.5 ($C_6H_5\text{-}CH_2\text{-}NH_2^+\text{-}CH_2\text{-}\underline{C}H_2\text{-}OH$); 129.2 ($\underline{C}_{Ar}H$); 129.6 ($\underline{C}_{Ar}H$); 129.8 ($\underline{C}_{Ar}H$); 130.5 ($\underline{C}_{Ar}$); 165.5 ($HO_2\underline{C}\text{-}\underline{C}O_2^-$).

15) N-benzylethanolammonium oxalate (16)

^{1}H NMR spectrum of **16** (D_2O, ppm): 3.13-3.26 m (4H, $C_6H_5\text{-}CH_2\text{-}NH_2^+\text{-}C\underline{H_2}\text{-}CH_2\text{-}OH$); 3.75-3.91 m (4H, $C_6H_5\text{-}CH_2\text{-}NH_2^+\text{-}CH_2\text{-}C\underline{H_2}\text{-}OH$); 4.28 s (4H, $C_6H_5\text{-}C\underline{H_2}\text{-}NH_2^+\text{-}CH_2\text{-}CH_2\text{-}OH$); 7.36-7.62 m ($10H_{Ar}$).

^{13}C NMR spectrum of **16** (D_2O, ppm): 48.4 ($C_6H_5\text{-}CH_2\text{-}NH_2^+\text{-}\underline{C}H_2\text{-}CH_2\text{-}OH$); 50.8 ($C_6H_5\text{-}\underline{C}H_2\text{-}NH_2^+\text{-}CH_2\text{-}CH_2\text{-}OH$); 56.5 ($C_6H_5\text{-}CH_2\text{-}NH_2^+\text{-}CH_2\text{-}\underline{C}H_2\text{-}OH$); 129.2 ($\underline{C}_{Ar}H$); 129.6 ($\underline{C}_{Ar}H$); 129.8 ($\underline{C}_{Ar}H$); 130.6 ($\underline{C}_{Ar}$); 173.5 ($^-O_2\underline{C}\text{-}\underline{C}O_2^-$).

Table S1. pH value of reaction mixtures for ^{68}Ga- and Al^{18}F-radiolabeling reactions

BEA Salt	pH of reaction mixture	
	**^{68}Ga-radiolabeling**	**Al^{18}F-radiolabeling**
**1**	4.1	6.1
**2**	5.4	6.3
**3**	3.0	5.5
**4**	5.1	5.4
**5**	4.3	5.8
**6**	3.9	5.7
**7**	2.6	5.4
**8**	2.9	4.3
**9**	1.8	3.6
**10**	2.7	4.7
**11**	3.9	6.0
**12**	4.2	5.8
**13**	2.8	5.5
**14**	5.5	7.5
**15**	1.6	4.6
**16**	4.1	4.3
